# Content and delivery of pre-operative interventions for patients undergoing total knee replacement: a rapid review

**DOI:** 10.1186/s13643-022-02019-x

**Published:** 2022-09-02

**Authors:** Anna M. Anderson, Benjamin T. Drew, Deborah Antcliff, Anthony C. Redmond, Christine Comer, Toby O. Smith, Gretl A. McHugh

**Affiliations:** 1 Leeds Institute of Rheumatic and Musculoskeletal Medicine, University of Leeds, Leeds, UK; 2 NIHR Leeds Biomedical Research Centre, Leeds, UK; 3 School of Healthcare, University of Leeds, Leeds, UK; 4 Physiotherapy Department, Bury Care Organisation, Northern Care Alliance NHS Group, Bury, UK; 5 School of Medicine, Keele University, Keele, UK; 6 Leeds Community Healthcare NHS Trust Musculoskeletal and Rehabilitation Services, Leeds, UK; 7 School of Health Sciences, University of East Anglia, Norwich, UK; 8 Nuffield Department of Orthopaedics, Rheumatology and Musculoskeletal Sciences, University of Oxford, Oxford, UK

**Keywords:** Total knee replacement, Total knee arthroplasty, Pre-operative care, Education, Prehabilitation, Exercise, Rapid review

## Abstract

**Background:**

Total knee replacement (TKR) is a common operation typically performed for end-stage knee osteoarthritis. Patients awaiting TKR often have poor health-related quality of life. Approximately 20% of patients experience persistent pain post-TKR. Pre-operative TKR interventions could improve pre- and post-operative outcomes, but future research is required to inform their design. This review aimed to identify and synthesize recent literature on the content and delivery of pre-operative TKR interventions to help guide future research and clinical practice.

**Methods:**

This rapid review included randomized trials of pre-operative TKR interventions (“outcomes studies”) and primary studies exploring patients’ and/or health professionals’ views of pre-operative TKR interventions (“views studies”). Medline, Embase, PsycINFO, CINAHL and the Cochrane Central Register of Controlled Trials were searched for English language studies published between January 2009 and December 2020. Eligible studies’ reference lists were screened. Studies were appraised using the Mixed Methods Appraisal Tool. The findings were narratively synthesized using a convergent segregated approach.

**Results:**

From 3263 records identified, 52 studies were included (29 outcomes studies, 21 views studies, two outcomes/views studies). The studies’ methodological quality varied but was generally highest in qualitative studies. The outcomes studies investigated education (*n*=5), exercise (*n*=20), psychological (*n*=2), lifestyle (*n*=1), and/or other interventions (*n*=5). The views studies addressed education (*n*=20), exercise (*n*=3), psychological (*n*=1), lifestyle (*n*=4), and/or other interventions (*n*=1). Only three outcomes studies (two randomized controlled trials (RCTs) and a pilot study) compared the effectiveness of intervention components/delivery approaches. The two RCTs’ results suggest that pre-operative TKR exercise interventions are equally effective regardless of whether they include strength or strength plus balance training and whether they are hospital- or home-based. Personal tailoring and using more than one delivery format were associated with improved outcomes and/or perceived as beneficial for multiple intervention types.

**Conclusions:**

Definitive evidence on the optimal design of pre-operative TKR interventions is lacking. Personal tailoring and employing multiple delivery formats appear to be valuable design elements. Preliminary evidence suggests that including balance training and hospital versus home delivery may not be critical design elements for pre-operative TKR exercise interventions.

**Systematic review registration:**

PROSPERO CRD42019143248

**Funder:**

National Institute for Health and Care Research (ICA-CDRF-2018-04-ST2-006).

**Supplementary Information:**

The online version contains supplementary material available at 10.1186/s13643-022-02019-x.

## Background

Total knee replacement (TKR) is a common elective operation typically performed in older people with end-stage knee osteoarthritis (OA) [1]. Internationally, the demand for TKR has risen dramatically over the past two decades due to factors such as ageing populations and rising obesity levels [2–4]. The COVID-19 pandemic has limited the capacity of services to meet this high demand. For example, approximately 97,000 TKR procedures were performed annually in the UK between 2016 and 2019, compared to approximately 45,000 in 2020 [5]. This has created a large backlog of patients awaiting TKR [6]. Correspondingly, estimates suggest TKR waiting times will continue to be at least 6 months longer than before the pandemic unless service provision is increased above pre-pandemic levels [6].

Long waiting times can profoundly affect patients. A cross-sectional study undertaken in 2020 found almost a quarter of patients awaiting TKR were in a health state “worse than death” ([7] p. 673). Furthermore, the study identified a direct correlation between increasing waiting times and deteriorating health-related quality of life [7]. Pre-pandemic studies have also demonstrated that patients awaiting TKR experience high and deteriorating levels of pain and functional limitations [8, 9]. These issues are particularly concerning because worse pre-operative pain and function are associated with poor outcomes following TKR [10, 11]. Poor TKR outcomes are a frequent problem, with estimates suggesting approximately 20% of patients experience persistent pain post-TKR [12].

By addressing modifiable predictors of poor TKR outcomes, pre-operative TKR interventions could help improve patient outcomes both pre- and post-operatively [13]. Pre-operative interventions often focus on prehabilitation—the process of improving patients’ pre-operative health and well-being to help them withstand the stresses of surgery and optimize their post-operative recovery [14, 15]. Prehabilitation programs can include multiple intervention types, such as exercise, psychological interventions, and health promotion [14, 15]. Education is another key type of pre-operative TKR intervention, which facilitates patients’ preparations for surgery and helps ensure that they have realistic outcome expectations [16, 17].

Although pre-operative TKR interventions offer many potential benefits, there are significant limitations in the evidence base supporting them. For example, a recent overview of reviews demonstrated that pre-operative exercise interventions for patients undergoing total joint replacement reduce length of hospital stay [18]. However, it was unable to establish whether the interventions improved any pre-operative outcomes, as none of the included reviews evaluated outcomes immediately post-intervention. Furthermore, most previous reviews of pre-operative TKR interventions have focused on evaluating intervention effectiveness. The few that have focused on intervention content and delivery have been limited to specific intervention types and/or study designs. For example, Louw et al. [19] reviewed the content and delivery of pre-operative education but only included four TKR studies, all of which were RCTs.

Reviewing evidence on intervention effectiveness and stakeholders’ perspectives is valuable for informing intervention development [20]. Correspondingly, a comprehensive review addressing the above gap in existing literature could help inform the development of pre-operative TKR interventions for future research and clinical practice.

This review aimed to identify and synthesize recent literature on the content and delivery of pre-operative TKR interventions. Its objectives were:

1. To identify what pre-operative TKR intervention components and delivery approaches are associated with improved outcomes among patients undergoing TKR.
2. To explore the experiences and perspectives of patients wait-listed for TKR, and their health professionals, on pre-operative TKR intervention components and delivery approaches.

This review formed part of the first phase of a mixed methods project aimed at developing a pre-operative education and prehabilitation digital intervention for patients listed for TKR. A key purpose of the review within the project was to inform an online modified Delphi study aimed at developing recommendations on pre-operative TKR education and prehabilitation [21].

## Methods

The review is reported according to the Preferred Reporting Items for Systematic Reviews and Meta-Analyses (PRISMA) guideline [22] (see Additional file 1 for completed PRISMA checklist). The review was registered with the International Prospective Register of Systematic Reviews (PROSPERO) on 3^rd^ September 2019 (CRD42019143248). The review protocol is available from the corresponding author. A Project Advisory Group, comprising an independent chair, four reviewers (AMA, ACR, CC, GAM), two patient representatives and a key collaborator, oversaw the review.

Rapid review methodology was adopted for the following reasons.

- The purpose of this review was to provide an overview of pre-operative TKR intervention components and delivery approaches, rather than definitive evidence about their effectiveness.
- Rapid reviews are considered acceptable for informing intervention development [20].
- Rapid reviews generally produce similar conclusions to systematic reviews [23].
- The review had to be completed in a defined period of time because its findings were required to inform the online modified Delphi study mentioned above [21].

Preliminary literature searches suggested that studies with varying designs would be relevant to the review’s aim. Furthermore, the review had two complementary objectives that address different aspects of the same phenomenon. A mixed methods convergent segregated design was therefore employed [24]. The review was informed by:

- SelecTing Approaches for Rapid Reviews (STARR) decision tool [25]
- World Health Organization rapid review guidance [26]
- Joanna Briggs Institute (JBI) mixed methods review guidance [24]

### Eligibility criteria

Mixed methods reviews are often described as including quantitative and qualitative components [24]. For the purpose of this review, the terms “outcomes studies” and “views studies” were chosen because studies of various designs can provide valuable information about peoples’ experiences/perspectives [27, 28]. Studies meeting the eligibility criteria specified in Table 1 were included.

**Table 1:** Eligibility criteria

	Outcomes studies	Views studies
Studies	Randomized trial (involve individual or cluster randomized allocation) Published as a full text in English between January 2009 and December 2020	Primary study of any design Published as a full text in English between January 2009 and December 2020
Participants^a^	Adults (aged ≥18 years) listed for primary TKR surgery^b^	Adults (aged ≥18 years) with experience of TKR care as: - a patient who is listed for and/or has undergone primary TKR surgery^b^ - a health professional with experience of any phase of the primary TKR pathway e.g. nurses, physiotherapists etc.
Interventions/comparator/outcomes/phenomena of interest	Include an intervention group that received a non-pharmacological pre-operative TKR intervention^c^ Include at least one comparator group that received no pre-operative TKR interventions, standard care and/or an alternative pre-operative TKR intervention Assess at least one patient outcome (including patient-reported outcomes, objectively measured clinical outcomes, patient healthcare utilization and patient harms)	Explore participants’ experiences and/or perspectives of at least one non-pharmacological pre-operative TKR intervention component or delivery approach^c^ (Studies providing purely descriptive accounts of non-pharmacological pre-operative interventions components and delivery approaches were excluded)
Context	No limitations	No limitations

*TKR* total knee replacement

^a^ Studies with mixed samples were only included if they reported at least one relevant finding separately for participants who met the criteria specified, and/or at least 80% of participants met the criteria specified [29]

^b^ No limitations regarding patients’ indication for TKR were applied

^c^ Pre-operative TKR interventions were defined as interventions delivered solely in the pre-operative phase of the TKR pathway (the period between when a patient is listed for TKR surgery and the day they are admitted to hospital to undergo surgery [30]). Studies investigating an intervention delivered during more than one phase of the TKR pathway were excluded

Only studies published from January 2009 onward were eligible because limiting a review’s scope by date is an accepted streamlining approach for rapid reviews [25, 26]. In addition, the specific start date chosen (2009) helped to ensure that the findings are relevant to current healthcare contexts. This was considered important because TKR enhanced recovery programs have become increasingly widespread since 2009. For example, the UK Department of Health implemented an Enhanced Recovery Partnership Program between 2009 and 2011 [31] and Denmark introduced a national enhanced recovery protocol for hip and knee replacements in 2009 [32]. Enhanced recovery programs affect multiple aspects of TKR pathways and have contributed to dramatic reductions in TKR length of hospital stay [32]. Short hospital stays mean it is particularly important that patients receive adequate pre-operative support to prepare for their discharge in advance [33].

The protocol specified that views studies would be eligible if they explored participants’ experiences and/or perspectives of at least one pre-operative TKR intervention. During the study selection process, it was decided to only include studies exploring participants’ experiences and/or perspectives of at least one pre-operative intervention component or delivery approach to ensure that all the included studies were directly relevant to the study aim.

### Search strategy

The following electronic databases were searched on 11 September 2019: Medline (Ovid), Embase (Ovid), PsycINFO (Ovid), CINAHL (EBSCOhost) and the Cochrane Central Register of Controlled Trials (Cochrane Library). All the searches were subsequently updated to 31 December 2020. The searches were conducted by one reviewer (AMA) using subject headings and text words related to TKR, the pre-operative phase and relevant interventions (see Additional file 2 for full search strategies). All searches were limited to human studies published between January 2009 and December 2020. Searches were also limited to studies published in the English language where possible. Reference lists of all eligible studies were screened. In line with accepted rapid review streamlining approaches, gray literature was not searched [26].

### Study selection

Following removal of duplicates, all records were screened for eligibility based on their title and abstract. Full-text reports of potentially relevant articles were then reviewed to identify studies for final inclusion. One reviewer (AMA) performed both steps. A second reviewer (BTD) verified the study selection for a randomly selected sample of 10% of all full-text reports reviewed. The random selection was made by numbering the reports and using the RANDBETWEEN function of Microsoft Excel 2016. Disagreements were resolved through reconciliation discussions.

### Data extraction and appraisal

One reviewer (AMA) extracted data using two standardized data extraction forms, one for outcomes studies and one for views studies. The data extraction forms covered general study information, study characteristics, participant characteristics, intervention overview and details (outcomes studies only) and study findings (see Additional file 3 for data items included in the data extraction forms). The intervention details data items were based on the Template for Intervention Description and Replication (TIDieR) checklist and guide [34]. The outcome studies’ interventions were classified as one or more of the following intervention types: education, exercise, psychological, lifestyle, and other. Interventions that included a brief educational component within a different intervention type were not classed as education. The term “other” was chosen to provide an inclusive category for any interventions that did not fit the definitions of the specified intervention types. The protocol listed the following examples of other pre-operative TKR interventions: orthotics, nutritional supplements, and acupuncture.

To facilitate the data syntheses, outcomes studies were dichotomized into two categories.

1. Studies in which a statistically significant difference in favor of the intervention group was identified for at least one outcome at one or more follow-up time points (alpha=0.05).
2. Studies in which no statistically significant differences in favor of the intervention group were identified for any outcomes at any follow-up time points (alpha=0.05).

The views studies’ findings were classified using the same intervention types and data items as the outcomes studies (see Additional file 3). Authors were not contacted to obtain missing data, which is a frequently used streamlining approach in rapid reviews [23].

Coding data about the intervention components and delivery approaches involved some subjective judgments due to the differing terminology and level of detail in the included reports. Furthermore, it was not always clear whether participants’ perspectives/experiences reported for views studies related to interventions delivered in the pre-operative phase. In cases of uncertainty, an inclusive approach was adopted to maximize the number of intervention components and delivery approaches identified. The lead reviewer (AMA) completed extensive crosschecking to ensure that the coding was consistent across studies and discussed key uncertainties with other reviewers. In addition, two reviewers (DA, CC) verified the data extraction for a randomly selected sample of 10% of the included studies. The random selection was made by numbering the studies and again using the RANDBETWEEN function of Microsoft Excel 2016. Disagreements were resolved through reconciliation discussions.

No studies were excluded from the review or syntheses based on their methodological quality to maximize the number of intervention components and delivery approaches identified. Appraisal of the included studies was still undertaken to assist with interpretation of their findings. One reviewer (AMA) conducted the appraisals using the Mixed Methods Appraisal Tool (MMAT) version 2018 [35]. The MMAT includes five categories of study designs, each with five methodological quality criteria. All studies were rated using the criteria for the relevant study design(s). Each criterion was rated as “Yes” if it was met, “No” if it was not met, or “Can’t tell” if insufficient information was available to rate the criterion. This meant that each study received between zero and five “Yes” ratings for each applicable MMAT category. Receiving “Yes” ratings only implies a study has high methodological quality. In line with the MMAT guidance, no overall scores were calculated. Second reviewers (DA, CC) verified the data extraction for the same randomly selected 10% of studies verified at the data extraction stage.

### Data syntheses

Narrative syntheses were used to summarize the data extracted for each intervention type. In line with a convergent segregated design, the outcomes studies and views studies were synthesized separately, then the two separate syntheses were integrated [24]. To facilitate the integration, the intervention components and delivery approaches from all studies investigating the same intervention type were juxtaposed in tables.

## Results

**Fig. 1:**
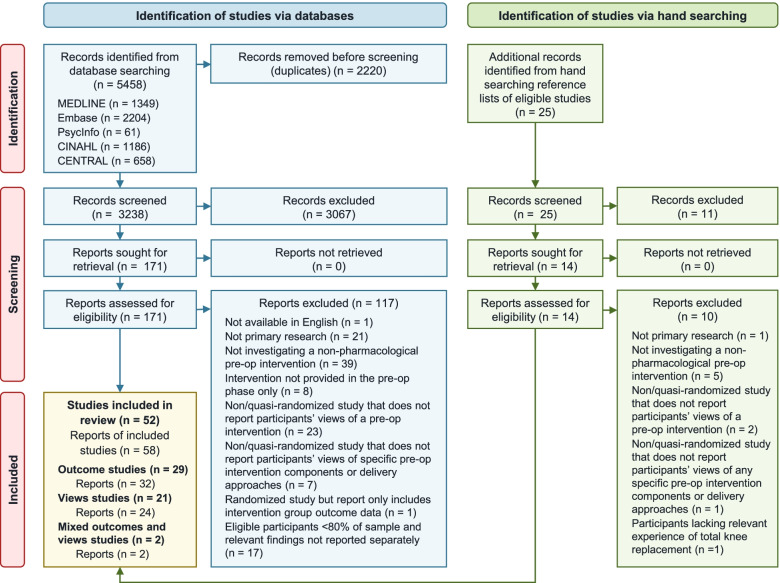
PRISMA flow diagram. *Pre-op* pre-operative

A total of 3238 non-duplicate records were identified from the database searches. A further 25 records were identified from hand searching. Fifty-eight reports, covering 52 studies, met the eligibility criteria (Fig. 1).

Key excluded studies of note were:

- A qualitative study that explored orthopedic surgeons’ and physiotherapists’ perceptions of a “pre-operative” exercise intervention ([36] p. 1). Whilst the intervention was described as “pre-operative”, it was delivered to potential candidates for TKR rather than patients listed for TKR; hence, it did not meet this review’s definition of a pre-operative intervention.
- An RCT that investigated an e-learning tool [37]. Intervention group participants received email invitations to access the tool pre- and post-operatively, so the intervention did not meet the criterion of being delivered solely pre-operatively. This RCT did not include any follow-up outcome assessments in the pre-operative phase; therefore, no data about the pre-operative impact of the tool were available.

### Outcomes study overview and appraisal

Thirty-one studies met the criteria for an outcomes study. Most of these investigated interventions classified as a single intervention type (*n*=29). The most commonly investigated intervention type was exercise (*n*=20). A pilot study involving 20 participants received “Yes” ratings for all the MMAT RCT criteria except outcome assessor blinding [38]. The MMAT RCT ratings of the other outcomes studies varied, but all received three or fewer “Yes” ratings. Table 2 summarizes the included outcomes studies and their MMAT ratings (see Additional file 4 for further details of the outcomes studies’ characteristics).

**Table 2:** Outcomes studies’ summaries and Mixed Methods Appraisal Tool ratings

Study summary			Mixed Methods Appraisal Tool Ratings: Quantitative Randomized Controlled Trials^**b**^				
Citation, country	Intervention type	Design^**a**^	Random allocation appropriately performed	Groups comparable at baseline	Complete outcome data	Outcome assessors blinded	Participants adhered to assigned intervention
Bergin et al., 2014 [39], USA	Other: Incentive spirometry	RCT	?	?	N	N	?
Blasco et al., 2020 [40], Spain	Exercise	Three-arm RCT	Y	Y	?	N	?
Brown et al., 2012 [41], USA	Exercise	Pilot study	?	?	N	N	Y
Brown et al., 2014 [42], USA	Exercise	RCT	Y	Y	N	N	?
Calatayud et al., 2017 [43], Casaña et al., 2019 [44], Spain	Exercise	RCT	Y	Y	Y	N	?
das Nair et al., 2018 [45]^c^, UK	Psychological	Mixed methods feasibility study	Y	Y	N	N	N
Doiron-Cadrin et al., 2020 [46], Canada	Exercise	Three-arm pilot study	Y	?	Y	N	?
Domínguez-Navarro et al., 2021 [47], Spain	Exercise	Three-arm RCT	Y	Y	N	N	Y
Eschalier et al., 2017 [48], France	Education	RCT	?	Y	Y	N	Y
Gränicher et al., 2020 [38], Switzerland	Exercise	Pilot study	Y	Y	Y	N	Y
Gstoettner et al., 2011 [49], Austria	Exercise	RCT	Y	Y	Y	N	?
Huber et al., 2015a [50], Switzerland	Exercise	RCT	Y	Y	N	N	Y
Jahic et al., 2018 [51], Bosnia and Herzegovina	Exercise	RCT	?	Y	?	N	?
Leal-Blanquet et al., 2013 [52], Spain	Education	RCT	?	Y	Y	N	?
Lin et al., 2019 [53], China	Education	RCT	?	Y	Y	N	?
Matassi et al., 2014 [54], Belgium	Exercise	RCT	?	Y	Y	N	Y
McKay et al., 2012 [55], Canada	Exercise	Pilot study	Y	Y	N	N	Y
Medina-Garzón, 2019 [56], Colombia	Psychological	RCT	?	Y	Y	N	?
Rittharomya et al., 2020 [57], Thailand	Exercise Lifestyle	RCT	?	Y	Y	N	?
Skoffer et al., 2016 [58], 2020 [59], Denmark	Exercise	RCT	Y	Y	N	N	Y
Soeters et al., 2018 [60], USA	Education	RCT	?	Y	?	N	Y
Soni et al., 2012 [61], UK	Exercise Other: Acupuncture	RCT	Y	Y	N	N	?
Stone et al., 2020 [62], USA	Other: Dynamic knee extension device	RCT	?	Y	Y	N	?
Swank et al., 2011 [63], USA	Exercise	RCT	?	Y	Y	N	Y
Topp et al., 2009 [64], USA	Exercise	RCT	?	Y	Y	N	?
Tungtrongjit et al., 2012 [65], Thailand	Exercise	RCT	?	Y	?	N	?
Villadsen et al., 2014a [66], 2014b [67], Denmark	Exercise	RCT	Y	Y	?	N	?
Walls et al., 2010 [68], Ireland	Other: NMES	Pilot study	?	?	N	N	Y
Wang et al., 2020 [69], China	Exercise	RCT	?	?	?	N	?
Wilson et al., 2016 [70], Canada	Education	RCT	Y	Y	N	N	?
Zhao et al., 2018 [71], China	Other: Electroacupuncture	RCT	Y	Y	Y	N	?

*N* no, *NMES* neuromuscular electrical stimulation, *RCT* randomized controlled trial, *UK* United Kingdom, *USA* United States of America, *Y* yes, *?* can’t tell

^a^ All studies involved two arms unless otherwise stated

^b^ For studies with mixed populations, ratings were made specifically for participants listed for total knee replacement

^c^ das Nair et al. (2018) was also appraised using the qualitative and mixed methods categories of the Mixed Methods Appraisal Tool (all ratings reported in Table 3)

### Views study overview and appraisal

Twenty-three studies met the criteria for a views study. Most of these addressed a single intervention type (*n*=19). The most frequently addressed intervention type was education (*n*=20). Eleven studies received “Yes” ratings only for the MMAT qualitative category. Across all the other MMAT categories, a single study received “Yes” ratings only [30]. Table 3 summarizes the included views studies and their MMAT ratings (see Additional file 5 for further details of the views studies’ characteristics).

**Table 3:** Views studies’ summaries and Mixed Methods Appraisal Tool ratings

**Study summary**			**Mixed Methods Appraisal Tool Ratings: Qualitative**^**b**^				
**Citation, country**	**Intervention type**	**Design**^**a**^	**Qualitative approach appropriate**	**Data collection methods adequate**	**Findings adequately derived from data**	**Interpretation sufficiently substantiated by data**	**Coherence between data sources, collection, analysis and interpretation**
Aunger et al., 2020 [72], UK	Lifestyle	Mixed methods feasibility study (ratings for qualitative descriptive component with data collection via participants’ sedentary behavior booklets and feasibility questionnaires)	Y	N	?	Y	Y
Bardgett et al., 2016 [73], UK	Education	Qualitative descriptive with data collection via a postal questionnaire	Y	N	Y	Y	Y
Berg et al., 2019 [74], Sweden	Education	Qualitative descriptive with data collection via semi-structured interviews	Y	Y	Y	Y	Y
Bin Sheeha et al., 2020 [75], UK	Education Exercise Other: Acupuncture	Phenomenological with data collection via a single focus group	Y	Y	Y	Y	Y
Causey-Upton and Howell, 2017 [76], USA	Education	Transcendental phenomenological with data collection via semi-structured interviews	Y	Y	Y	Y	Y
Causey-Upton et al., 2020b [77], USA	Education	Explanatory sequential mixed methods (ratings for qualitative descriptive component with data collection via semi-structured interviews)	Y	Y	Y	Y	Y
das Nair et al., 2018 [45], UK	Psychological	Mixed methods feasibility study (ratings for qualitative component with data collection via semi-structured interviews)	Y	Y	Y	Y	Y
Drew et al., 2019 [78], Judge et al., 2020 [79], UK	Education	Ethnography with data collection via observations/job shadowing and semi-structured interviews	Y	Y	Y	?	Y
Goldsmith et al., 2017 [80]^c^, Canada	Education	Qualitative descriptive component of a mixed methods prospective cohort study with data collection via semi-structured interviews	Y	Y	Y	Y	Y
Høvik et al., 2018 [81], Norway	Education	Qualitative descriptive with data collection via focus groups	Y	Y	Y	Y	Y
Lucas et al., 2013a [82] , 2013b [83], UK	Education	Action research study	Y	Y	Y	Y	Y
Sharif et al., 2020 [84], UK	Education Exercise	Qualitative descriptive with data collection via semi-structured interviews	Y	Y	Y	Y	Y
Smith et al., 2018 [85], USA	Education	Qualitative descriptive with data collection via open-ended, structured interviews	Y	N	?	?	N
Snowden et al., 2020 [86], UK	Lifestyle Education (education addressed briefly in the feasibility study qualitative component)	Mixed methods involving a non-randomized feasibility study followed by a pilot study (ratings for qualitative descriptive components of the feasibility study and pilot study with data collection via focus groups and interviews)	Y	Y	Y	Y	Y
Specht et al., 2016 [87], Denmark	Education	Phenomenological-hermeneutic with data collection via observations and semi-structured interviews	Y	Y	Y	Y	Y
**Study summary**			**Mixed Methods Appraisal Tool Ratings: Quantitative Randomized Controlled Trials**^**b**^				
**Citation, country**	**Intervention type**	**Design**^**a**^	**Random allocation appropriately performed**	**Groups comparable at baseline**	**Complete outcome data**	**Outcome assessors blinded**	**Participants adhered to assigned intervention**
Aunger et al., 2020 [72]^d^, UK	Lifestyle	Mixed methods feasibility study (ratings for quantitative component)	Y	?	?	N	?
das Nair et al., 2018 [45], UK	Psychological	Mixed methods feasibility study (ratings for quantitative component)	Y	Y	N	N	N
Eschalier et al., 2017 [48], France	Education	RCT	?	Y	Y	N	Y
Snowden et al., 2020 [86]^d^, UK	Lifestyle Education	Mixed methods involving a non-randomized feasibility study followed by a pilot study (ratings for quantitative component of pilot study)	Y	?	?	N	Y
**Study summary**			**Mixed Methods Appraisal Tool Ratings: Quantitative Non-Randomized Studies**^**b**^				
**Citation, country**	**Intervention type**	**Design**^**a**^	**Participants representative of target population**	**Measurements appropriate**	**Complete outcome data**	**Confounders accounted for**	**Intervention administered as intended**
Snowden et al., 2020 [86], UK	Lifestyle Education	Mixed methods involving a non-randomized feasibility study followed by a pilot study (ratings for quantitative component of feasibility study)	N	Y	Y	Y	Y
**Study summary**			**Mixed Methods Appraisal Tool Ratings: Quantitative Descriptive**^**b**^				
**Citation, country**	**Intervention type**	**Design**^**a**^	**Relevance of sampling strategy**	**Sample representative of target population**	**Measurements appropriate**	**Risk of non-response bias low**	**Statistical analysis appropriate**
Barnes et al., 2018 [88], South Africa	Education	Cross-sectional survey with data collection via structured interviews	Y	?	N	?	Y
Causey-Upton et al., 2018 [89], USA	Education	Cross-sectional online “pilot” survey	N	N	Y	N	Y
Causey-Upton et al., 2020a [90], USA	Education	Explanatory sequential mixed methods (ratings for cross-sectional survey)	Y	?	Y	N	Y
Eschalier et al., 2013 [91], France	Education	Survey embedded within an intervention validation study	?	?	Y	?	Y
Huber et al., 2015b [92], Switzerland	Education	Questionnaire development and psychometric testing embedded within an RCT	?	?	Y	?	Y
Plenge et al., 2018 [93], South Africa	Lifestyle	Delphi study	N	N	Y	?	Y
SooHoo et al., 2011 [94], USA	Education	Modified Delphi study	Y	?	N	?	Y
Snowden et al., 2020 [86], UK	Lifestyle Education	Mixed methods involving a non-randomized feasibility study followed by a pilot study (ratings for the COM-B questionnaire component of the intervention development)	?	?	Y	?	Y
Westby et al., 2018 [30], Canada	Education Exercise Lifestyle	Modified Delphi study	Y	Y	Y	Y	Y
**Study summary**			**Mixed Methods Appraisal Tool Ratings: Mixed Methods**^**b**^				
**Citation, country**	**Intervention type**	**Design**^**a**^	**Adequate rationale for mixed methods design**	**Different study components effectively integrated**	**Outputs of the integration adequately interpreted**	**Divergences and in-consistencies adequately addressed**	**Different components adhered to corresponding quality criteria**
Aunger et al., 2020 [72], UK	Lifestyle	Mixed methods feasibility study (ratings for overall study)	Y	N	N	Y	N
Causey-Upton et al., 2020a [90], 2020b [77], USA	Education	Explanatory sequential mixed methods (ratings for overall study)	N	N	N	Y	N
das Nair et al., 2018 [45], UK	Psychological	Mixed methods feasibility study (ratings for overall study)	?	Y	Y	Y	N
Snowden et al., 2020 [86], UK	Lifestyle Education	Mixed methods involving a non-randomized feasibility study followed by a pilot study (ratings for overall study)	?	Y	Y	Y	N

*COM-B questionnaire* Adapted version of the Capability, Opportunity, Motivation and Behavior model self-evaluation questionnaire*; N* no; *RCT* randomized controlled trial; *UK* United Kingdom; *USA* United States of America; *Y* yes; *?* can’t tell

^a^ All RCTs, pilot and feasibility studies involved two arms unless otherwise stated

^b^ For studies with mixed populations, ratings were made specifically for participants who met the review eligibility criteria

^c^ Reported the qualitative component of a mixed methods study, but the quantitative results are not reported in the same article; therefore, the study was appraised using the qualitative category of the Mixed Methods Appraisal Tool only.

^d^ Quantitative outcome data were not presented separately for participants undergoing knee replacement; therefore, the study does not meet the criteria for an outcomes study

### Education interventions

#### Outcomes studies

Five outcomes studies investigated pre-operative education interventions (Table 4).

**Table 4:** Education intervention components and delivery approaches

Category	Intervention component or delivery approach	Leal-Blanquet et al. [52]^**a**^	Lin et al. [53]^**a**^	Soeters et al. [60]^**a**^	Wilson et al. [70]^**b**^	Eschalier et al. [48]^**a**^	Bardgett et al. [73]	Barnes et al. [88]	Berg et al. [74]	Bin Sheeha et al. [75]	Causey-Upton and Howell [76]	Causey-Upton et al. [89]	Causey-Upton et al. [77, 90]
Component: Education topic	Knee joint anatomy					O						V	V
	Osteoarthritis					O							
	Alternative treatment options to TKR					O							
	Joint replacements in younger people												
	Pre-op exercise/purpose of pre-op rehabilitation					O							V
	Patient active involvement in their own management					O				V			
	Goal setting			O									
	Obtaining/using walking aids and other equipment			O									V
	Making home preparations			O		V						V	V
	Arranging social support					V							
	Transport												V
	What to expect during the hospital stay	O		O		V			V^c^				V
	TKR surgical procedure			O		OV			V				V
	Risks of TKR surgery					V			V^c^				
	Precautions		O	O	O	O							V
	Pain expectations	O											V
	Discharge instructions/what to expect following discharge	O		O		O							
	Recovery expectations			O		O			V			V	V
	Pain management		O		O	O							V
	Asking for antiemetics				O								
	Edema management												V
	Wound healing												V
	Rehabilitation	O		O		O			V			V	V
	Alternative and supplementary rehabilitation options												
	Addressing sleep difficulties												V
	Returning to daily activities	O		O		O		V				V	V
	Returning to sports					O							
	Returning to work					O	V						
	Physical activity					O							
	Nutrition												V
	Comorbidities												V
	Other patients’ experiences of TKR	O											
Component: Activity/ opportunity	Allow participation of a relative or other support										V		V
	Combination of theory and active participation												
	Asking/addressing questions	O		O	O						V		V
	Practicing transfers, stairs, post-op exercises and/or mobilizing with or without aids			O									
	Goal setting			O									
	Documentation of concerns, strategies and questions				O								
Provider	Multidisciplinary team												V
	Researcher(s)				O								
	Orthopaedic surgeon									V		V	
	Physician assistant											V	
	Nurse/nurse practitioner	O	O										V
	Physical therapist/physiotherapist			O									V
	Occupational therapist												V
	Case manager											V	V
	Social worker											V	V
	Previous patients												V
	Pain management provider												V
	Home healthcare provider												V
	Dietetics												V
	Anesthetist												V
	Pharmacist												V
	Diabetes educator												V
	Hospital concierge												V
	Translator												V
Delivery mode: Format	Single format					O							V
	>1 format	O	O	O	O								V
	Individual			O	O								V
	Group												V
	Face-to-face/visit/session	O	O	O	O								
	Verbal												V
	Booklet/other written paper format		O		O	O	V						V
	Video/DVD	O		O								V	V
	Website/mobile health/other electronic format			O									V
	Virtual reality												
	Telecommunication				O								
	PowerPoint presentation												V
	Workbook												V
	Client demonstration or teach back method												V
	Healthcare provider demonstration												V
Delivery mode: Delivery with other patients	Delivered separately from education delivered to patients waiting for other orthopedic surgery											V	V
	Delivered with patients waiting for other orthopedic surgery												V
Setting	Pre-op assessment clinic				O								
Schedule: Number of sessions (contacts with education providers)	0 sessions												V
	1 session	O	O	O									V
	2 sessions				O								V
	3 sessions												V
	≥5 sessions												V
Schedule: Timing of delivery	1 day pre-op		O										
	<1 week pre-op				O								V
	~2 weeks pre-op			O									V
	3 weeks pre-op												V
	~4 weeks pre-op	O											V
	≤4 weeks pre-op				O								
	4–6 weeks pre-op					O							
	6 or ≥8 weeks pre-op												V
	Immediately after listing for surgery												
	Far in advance of surgery												V
	Close to surgery												V
	On the same day as other appointments, e.g., pre-op assessment or physician appointments	O		O	O	O							V
Intensity: Duration of sessions (contacts with education providers)	<15, 15–29 or 30–59 min session												V
	~20–30 min session			O									
	~45 min session, including watching a 10-min DVD twice	O											
	1 to <1.5, 1.5 to <2, 2 to <2.5, 2.5 to <3 or >3 h session												V
Intensity: Quantity of information	12 page booklet				O								
	Large volume of information												
Tailoring	Tailored to patient-specific needs/individualized			O	O		V^c^						V
	Consistent education for all patients												V
	Lateralized (right versus left)			O									
	Tailored to patients undergoing TKR			O									

Category	Intervention component or delivery approach	Drew et al. [78] Judge et al. [79]	Eschalier et al. [91]	Goldsmith et al. [80]	Høvik et al. [81]	Huber et al. [92]	Lucas et al. [82, 83]	Sharif et al. [84]	Smith et al. [85]	Snowden et al. [86]	SooHoo et al. [94]	Specht et al. [87]	Westby et al. [30]
Component: Education topic	Knee joint anatomy		V			V							
	Osteoarthritis		V										
	Alternative treatment options to TKR		V								V		
	Joint replacements in younger people	V^c^											
	Pre-op exercise/purpose of pre-op rehabilitation												
	Patient active involvement in their own management												
	Goal setting												
	Obtaining/using walking aids and other equipment	V					V						V
	Making home preparations	V^c^									V		V
	Arranging social support	V^c^											
	Transport												V
	What to expect during the hospital stay												
	TKR surgical procedure		V										V
	Risks of TKR surgery			V							V		V
	Precautions		V										
	Pain expectations			V					V				
	Discharge instructions/what to expect following discharge		V										V
	Recovery expectations	V^c^		V							V		V
	Pain management			V					V				V
	Asking for antiemetics												
	Edema management												
	Wound healing												
	Rehabilitation	V^c^	V	V		V	V						V
	Alternative and supplementary rehabilitation options			V									
	Addressing sleep difficulties												
	Returning to daily activities		V			V							
	Returning to sports		V										
	Returning to work		V										
	Physical activity												
	Nutrition												
	Comorbidities												
	Other patients’ experiences of TKR			V									
Component: Activity/ opportunity	Allow participation of a relative or other support											V^c^	V
	Combination of theory and active participation					V							
	Asking/addressing questions			V		V		V				V	V
	Practicing transfers, stairs, post-op exercises and/or mobilizing with or without aids						V						
	Goal setting												
	Documentation of concerns, strategies and questions												
Provider	Multidisciplinary team	V											V
	Researcher(s)												
	Orthopaedic surgeon			V							V		
	Physician assistant												
	Nurse/nurse practitioner						V				V		
	Physical therapist/physiotherapist						V						
	Occupational therapist						V						
	Case manager										V		
	Social worker												
	Previous patients						V						
	Pain management provider												
	Home healthcare provider												
	Dietetics												
	Anesthetist												
	Pharmacist												
	Diabetes educator												
	Hospital concierge												
	Translator												
Delivery mode: Format	Single format												
	>1 format										V		
	Individual											V^c^	
	Group	V			V							V	
	Face-to-face/visit/session	V		V								V	V
	Verbal	V^c^							V			V	
	Booklet/other written paper format	V	V		V	V					V	V	V
	Video/DVD							V			V		V
	Website/mobile health/other electronic format							V			V		
	Virtual reality							V					
	Telecommunication							V					V
	PowerPoint presentation					V							
	Workbook												
	Client demonstration or teach back method												
	Healthcare provider demonstration												
Delivery mode: Delivery with other patients	Delivered separately from education delivered to patients waiting for other orthopedic surgery												
	Delivered with patients waiting for other orthopedic surgery			V									
Setting	Pre-op assessment clinic									V			
Schedule: Number of sessions (contacts with education providers)	0 sessions												
	1 session												
	2 sessions												
	3 sessions					V							
	≥5 sessions												
Schedule: Timing of delivery	1 day pre-op												
	<1 week pre-op												
	~2 weeks pre-op												
	3 weeks pre-op												
	~4 weeks pre-op												
	≤4 weeks pre-op												
	4–6 weeks pre-op												
	6 or ≥8 weeks pre-op												
	Immediately after listing for surgery											V	
	Far in advance of surgery	V^c^											
	Close to surgery	V^c^											
	On the same day as other appointments, e.g., pre-op assessment or physician appointments												
Intensity: Duration of sessions (contacts with education providers)	<15, 15–29 or 30–59 min session												
	~20–30 min session												
	~45 min session, including watching a 10-min DVD twice												
	1 to <1.5, 1.5 to <2, 2 to <2.5, 2.5 to <3 or >3 h session												
Intensity: Quantity of information	12 page booklet												
	Large volume of information								V	V		V	
Tailoring	Tailored to patient-specific needs/individualized			V				V					
	Consistent education for all patients												
	Lateralized (right versus left)												
	Tailored to patients undergoing TKR												

*DVD* audiovisual videodisc, *O* intervention component/delivery approach included in an intervention investigated in an outcomes study, *pre-op* pre-operative, *TKR* total knee replacement, *V* participants’ experiences/perspectives of the intervention component/delivery approach reported in a views study

^a^ Outcomes study in which a statistically significant difference in favor of the intervention group was identified for at least one outcome at one or more follow-up time points (alpha=0.05)

^b^ Outcomes study in which no statistically significant differences in favor of the intervention group were identified for any outcomes at any follow-up time points (alpha=0.05)

^c^ Finding is from a study with a mixed population and is not supported with evidence specifically for participants who met the review eligibility criteria

In four studies, superior outcomes in the intervention group were identified for the number of physical therapy visits required and time taken to meet inpatient physical therapy discharge criteria [60], expectations/change in expectations on specific topics [52], knowledge/change in knowledge [48, 53], change in specific beliefs [48] and/or pain [53]. The commonest education topics covered by these studies’ interventions were precautions (e.g. falls prevention), discharge instructions/information, rehabilitation, and returning to daily activities. The commonest overall delivery approach involved using more than one format with a single session delivered by a nurse or physical therapist.

The study by Wilson et al. [70] did not identify any superior outcomes in the intervention group. This study’s intervention focused predominantly on pain management and was delivered using a booklet, individual teaching session and follow-up telephone call by the principal investigator (PI).

#### Views studies

Twenty views studies reported participants’ views of pre-operative education intervention components and/or delivery approaches (Table 4). Key findings included the following.

1. Value of comprehensive pre-operative education
  Patients and health professionals emphasized the value of multiple education topics. The most frequently mentioned were rehabilitation (*n*=9) and recovery expectations (*n*=7). Despite the apparent value of comprehensive education, health professionals highlighted that receiving a large volume of information could be difficult for patients to process [86] or result in “information fatigue” ([85] p. 187).
2. Importance of appropriate pre-operative education delivery
  The approaches used to deliver pre-operative education appeared to influence its value. For example, patients had difficulties remembering information provided straight after deciding to undergo surgery [87]. Positives and negatives were highlighted for specific delivery approaches. For example, both patients and health professionals highlighted benefits of group sessions, including the opportunity to interact with peers [77, 79, 81, 87]. Conversely, hearing peers discussing serious complications could be frightening for patients [81]. Employing multiple delivery formats was suggested to help account for patients’ varying needs [77].
3. Insufficiencies in pre-operative education
  Patients highlighted insufficiencies in certain education topics, such as rehabilitation [74, 80], recovery expectations [74, 79, 80] and return to work [73]. Furthermore, some patients felt that the pre-operative education they received was insufficiently tailored to their individual needs [73, 80].

#### Integration of the outcomes studies and views studies

Table 4 juxtaposes the education intervention components and delivery approaches identified in the outcomes studies and views studies. Of all the intervention components identified, 55% were noted in both study types. Contrastingly, only 29% of the delivery approaches were noted in both study types. The latter is partly attributable to the large number of delivery approaches identified in the views study by Causey-Upton et al. [77, 90].

The integration highlights factors that may have contributed to the lack of intervention benefits identified by Wilson et al. [70]. For example, the intervention covered pain management, asking for antiemetics and preventing dehydration, rather than a comprehensive range of topics. In contrast, three of the four RCTs that identified superior outcomes in the intervention group investigated interventions covering at least six topics [48, 52, 60]. Furthermore, all three of these interventions covered rehabilitation, the most frequently mentioned topic in the views studies.

### Exercise interventions

#### Outcomes studies

Twenty outcomes studies investigated pre-operative exercise interventions (Table 5).

**Table 5:** Exercise intervention components and delivery approaches

Category	Intervention component or delivery approach	Blasco et al. [40]^**a**^	Brown et al. [41]^**a**^	Brown et al. [42]^**b**^	Calatayud et al. [43]^**a**^ Casaña et al. [44]^**a**^	Doiron-Cadrin et al. [46]^**a**^	Domínguez-Navarro et al. [47]^**a**^	Gränicher et al. [38]^**a**^	Gstoettner et al. [49]^**a**^	Huber et al. [50]^**b**^	Jahic et al. [51]^**a**^	Matassi et al. [54]^**a**^	McKay et al. [55]^**bc**^
Component: Exercise type	Single exercise type												
	>1 exercise type	OH OD	O	O	O	OI OT	OS OB	O	O	O	O	O	O
	Warm-up^d^		O	O	O	OI OT	OS OB		O	O			O
	Upper body strength exercises		O	O									
	Lower limb strength/resistance exercises	OH OD	O	O	O	OI OT	OS OB			O	O	O	O
	Upper limb stretches		O	O									
	Trunk stretches		O	O									
	Lower limb stretches/flexibility training		O	O					O			O	
	Lower limb joint mobilization/range of movement exercises					OI OT							
	Lower limb PNF techniques							O					
	Endurance training							O					
	Step training		O	O									
	Proprioceptive/balance exercises	OH OD			O	OI OT	OB		O				
	Postural orientation/functional alignment exercises									O			
	Core stability/postural function exercises									O			
	Functional exercises									O			
	Gait training, including instruction on using walking aids												
	Cool down^f^		O	O	O		OS OB			O			
Component: Adjunct/ activity	Educational component^g^					OI OT		O					
	Individually indicated interventions^h^							O					
	Walking aid adjustments					OI OT							
	Completion of a log book/calendar	OD	O	O		OI OT						O	
	Documentation of physical activity per training week									O			
	Pain self-monitoring during and after training									O			
	Remote monitoring by a professional(s)	OD											
	Goal setting			O									
	Behavioral contracting			O									
	Opportunity to ask questions												
	Feedback/praise/encouragement from professionals			O						O			O
Provider	Physical therapist/physiotherapist(s)	OH OD			O	OI OT	OS OB	O	O	O			
	Nurses												
	Kinesiologist												O
	Researcher(s)		O	O								O	
Delivery mode	Single format	OH			O		OS OB			O			O
	>1 format	OD	O	O		OI OT		O	O			O	
	Supervised sessions	OH	O	O	O	OI	OS OB	O	O	O			O
	Telecommunication-delivered sessions, e.g., via telephone or a web application					OT							
	Optional in-person appointment if pain increased					OT							
	Instruction session (prior to unsupervised sessions)	OD										O	
	Information/ instruction sessions with a poster and DVD (prior to unsupervised sessions)												
	Review session (after 2 weeks of unsupervised sessions)	OD											
	Unsupervised sessions	OD	O	O		OI OT		O	O			O	
	Booklet/other written instructions		O	O					O			O	
	Website/mobile health					OT							
	Remote monitoring e.g. via telephone calls/mobile applications	OD											
Setting^i^	Home and clinical setting		O	O		OI		O					
	Home and unspecified location for supervision												
	Home	OD				OT					O	O	
	Clinical setting	OH					OS OB						
	Research facility												O
Schedule: Session frequency	3 × daily										O		
	Daily					OI OT			O				
	5 × weekly											O	
	3 × weekly	OH OD	O	O	O		OS OB						O
	≥3 × weekly												
	2 × weekly									O			
	5–9 supervised sessions within 3–4 weeks before surgery							O					
	Weekly for 4 weeks, then fortnightly for 4 weeks, then monthly until surgery												
Schedule: Program length	3 weeks												
	4 weeks	OH OD					OS OB						
	4–8 weeks												
	4–12 weeks									O			
	6 weeks								O		O	O	O
	8 weeks		O	O	O								
	Start ≥8 weeks before surgery												
	12 weeks					OI OT							
	Months (rather than days/weeks)												
	Variable depending on time available pre-operatively									O			
Intensity: Session duration	~30 min												O
	30–40 min						OS						
	45min								O				
	~50 min		O	O									
	45–60 min						OB						
	60 min									O			
Intensity: Warm-up duration	5 min (unweighted leg joint movements or walking)		O	O									
	5–10 min (walking)								O				
	10 min (aerobic activities)				O	OI OT	OS OB			O			O
	15 min (total duration)				O								
	Perceived exertion “‘somewhat hard” (leg cycling)									O			
	2 sets of 20 reps (step-ups, calf raises)				O								
	5 reps per “extension”												
Intensity: Strength/ resistance exercises	High intensity				O								
	Low to moderate resistance												
	Moderately fatiguing		O	O									
	“As tolerated”												
	1 set of 10 reps (≥6 s hold per rep)												
	1–2 sets of 10 reps		O	O									
	2 sets of 10–12 reps					OI OT							
	2–3 sets of 10–15 reps									O			
	4 sets of 10 reps	OH OD											
	5–10 s hold × 30 or 10 s hold × 10												
	1 warm-up set of 10 reps with light resistance, then 5 sets of 10 reps at 10 RM				O								
	Maximum 20 reps											O	
	2 sets of 8 reps starting at 60% of 1 RM and increasing by 1–2 kg per week as tolerated (except for calf raises, which were performed with body weight only)												O
	3 sets at 12 RM progressing to 8 RM												
	3 sets of 10 reps at 50% 10 RM with/without progression to 100% RM; or 5 min per exercise						OS OB						
	10–20%, 30–50% or 50–80% of 1 RM							O					
	60–100 daily												
Intensity: Flexibility exercises (stretches)	2 sets of 20 s		O	O									
	3 sets of 20 s								O				
	3 sets of 30 s												
	4 sets of 30 s											O	
Intensity: Endurance training	10–45 min at 40–70% maximum HR without pain provocation							O					
Intensity: Step training	1 set of 8 reps, progressing to 1 set of 20 reps per direction		O	O									
Intensity: Proprioceptive/ balance exercises	30 s per exercise					OI OT							
	3–4 sets of 30–60 s per exercise							O					
	4 sets of 15 or 30 s per exercise				O								
	1 rep, 10–15 reps or 10 s hold per exercise with eyes open then eyes closed if possible								O				
	2–4 min per exercise	OH OD											
	5 min per exercise						OB						
Intensity: Function-focused exercises	2–3 sets of 1–15 reps									O			
Intensity: Cool down	5 min (unweighted leg joint movements, stretches or walking)		O	O	O								
	10 min (total duration)									O			
	2 sets of 30 s (stretches)						OS OB						
	10 min (ice application)						OS OB						
Intensity: Progression	Progressive	OH OD	O	O		OI OT	OS OB		O	O		O	O
Tailoring	Tailored according to needs/ability/individualized	OH OD	O	O	O	OI OT	OS OB	O	O	O			O

Category	Intervention component or delivery approach	Rittharomya et al. [57]^**a**^	Skoffer et al. [58, 59]^**a**^	Soni et al. [61]^**b**^	Swank et al. [63]^**a**^	Topp et al. [64]^**a**^	Tungtrongjit et al. [65]^**a**^	Villadsen et al. [66, 67] ^**a**^	Wang et al. [69]^**a**^	Bin Sheeha et al. [75]	Sharif et al. [84]	Westby et al. [30]
Component: Exercise type	Single exercise type	O					O					
	>1 exercise type		O	O	O	O		O	O			V
	Warm-up^d^		O		O	O		O	O			
	Upper body strength exercises											V
	Lower limb strength/resistance exercises	O^e^	O	O^e^	O	O	O	O	O			V
	Upper limb stretches											
	Trunk stretches											
	Lower limb stretches/flexibility training		O	O^e^								
	Lower limb joint mobilization/range of movement exercises											V
	Lower limb PNF techniques											
	Endurance training											
	Step training			O^e^	O	O						
	Proprioceptive/balance exercises			O^e^					O			
	Postural orientation/functional alignment exercises							O				
	Core stability/postural function exercises			O^e^				O				
	Functional exercises			O^e^				O	O			V
	Gait training, including instruction on using walking aids											V
	Cool down^f^				O	O		O				
Component: Adjunct/ activity	Educational component^g^	O										V
	Individually indicated interventions^h^											
	Walking aid adjustments											
	Completion of a log book/calendar	O			O	O						
	Documentation of physical activity per training week											
	Pain self-monitoring during and after training							O				
	Remote monitoring by a professional(s)	O										
	Goal setting								O			
	Behavioral contracting											
	Opportunity to ask questions	O										
	Feedback/praise/encouragement from professionals	O	O					O				
Provider	Physical therapist/physiotherapist(s)		O	O				O		V		
	Nurses								O			
	Kinesiologist											
	Researcher(s)	O			O	O			O			
Delivery mode	Single format		O	O				O				
	>1 format	O			O	O	O		O			
	Supervised sessions		O	O	O	O		O	O			
	Telecommunication-delivered sessions, e.g., via telephone or a web application						O					
	Optional in-person appointment if pain increased											
	Instruction session (prior to unsupervised sessions)											
	Information/instruction sessions with a poster and DVD (prior to unsupervised sessions)	O										
	Review session (after 2 weeks of unsupervised sessions)											
	Unsupervised sessions	O			O	O	O					
	Booklet/other written instructions				O				O			
	Website/mobile health										V	
	Remote monitoring e.g. via telephone calls/mobile applications	O									V	
Setting^i^	Home and clinical setting				O							
	Home and unspecified location for supervision					O						
	Home	O					O					
	Clinical setting		O	O								
	Research facility											
Schedule: Session frequency	3 × daily						O					
	Daily											
	5 × weekly											
	3 × weekly	O	O						O			
	≥3 × weekly				O	O						
	2 × weekly							O				
	5–9 supervised sessions within 3–4 weeks before surgery											
	Weekly for 4 weeks, then fortnightly for 4 weeks, then monthly until surgery			O								
Schedule: Program length	3 weeks						O					
	4 weeks		O						O			
	4–8 weeks				O							
	4–12 weeks											
	6 weeks											
	8 weeks							O				
	Start ≥8 weeks before surgery											V
	12 weeks	O										
	Months (rather than days/weeks)									V		
	Variable depending on time available pre-operatively					O						
Intensity: Session duration	~30 min											
	30–40 min								O			
	45min											
	~50 min											
	45–60 min											
	60 min		O					O				
Intensity: Warm-up duration	5 min (unweighted leg joint movements or walking)				O	O						
	5–10 min (walking)											
	10 min (aerobic activities)		O					O				
	15 min (total duration)											
	Perceived exertion “‘somewhat hard” (leg cycling)							O				
	2 sets of 20 reps (step-ups, calf raises)											
	5 reps per “extension”								O			
Intensity: Strength/ resistance exercises	High intensity		O									
	Low to moderate resistance				O							
	Moderately fatiguing				O							
	“As tolerated”											V
	1 set of 10 reps (≥6 s hold per rep)						O					
	1–2 sets of 10 reps				O							
	2 sets of 10–12 reps											
	2–3 sets of 10–15 reps							O				
	4 sets of 10 reps											
	5–10 s hold × 30 or 10 s hold × 10								O			
	1 warm-up set of 10 reps with light resistance, then 5 sets of 10 reps at 10 RM											
	Maximum 20 reps											
	2 sets of 8 reps starting at 60% of 1 RM and increasing by 1–2 kg per week as tolerated (except for calf raises, which were performed with body weight only)											
	3 sets at 12 RM progressing to 8 RM		O									
	3 sets of 10 reps at 50% 10 RM with/without progression to 100% RM; or 5 min per exercise											
	10–20%, 30–50% or 50–80% of 1 RM											
	60–100 daily	O										
Intensity: Flexibility exercises (stretches)	2 sets of 20 s											
	3 sets of 20 s											
	3 sets of 30 s		O									
	4 sets of 30 s											
Intensity: Endurance training	10–45 min at 40–70% maximum HR without pain provocation											
Intensity: Step training	1 set of 8 reps, progressing to 1 set of 20 reps per direction				O							
Intensity: Proprioceptive/ balance exercises	30 s per exercise											
	3–4 sets of 30–60 s per exercise											
	4 sets of 15 or 30 s per exercise											
	1 rep, 10–15 reps or 10 s hold per exercise with eyes open then eyes closed if possible											
	2–4 min per exercise											
	5 min per exercise											
Intensity: Function-focused exercises	2–3 sets of 1–15 reps							O				
Intensity: Cool down	5 min (unweighted leg joint movements, stretches or walking)				O	O						
	10 min (total duration)							O				
	2 sets of 30 s (stretches)											
	10 min (ice application)											
Intensity: Progression	Progressive		O		O			O				V
Tailoring	Tailored according to needs/ability/individualized		O					O	O			V

*B* strengthening plus balance/proprioceptive exercise group, *D* domiciliary group, *H* hospital group, *HR* heart rate, *I* in-person prehabilitation group, *O* intervention component/delivery approach included in an intervention investigated in an outcomes study, *PNF* proprioceptive neuromuscular facilitation, *reps* repetitions, *RM* repetition maximum, *S* strengthening group, *T* telerehabilitation prehabilitation group, *V* participants’ experiences/perspectives of the intervention component/delivery approach reported in a views study

^a^ Outcomes study in which a statistically significant difference in favor of the intervention group was identified for at least one outcome at one or more follow-up time points (alpha=0.05)

^b^ Outcomes study in which no statistically significant differences in favor of the intervention group were identified for any outcomes at any follow-up time points (alpha=0.05)

^c^ The control group participated in an upper body strength training program that involved the same warm-up and delivery approaches as the intervention group’s lower limb strength training program

^d^ Warm-up included at least one of the following activities: aerobic activities e.g. cycling or walking; joint movements; and/or dynamic body weight exercises

^e^ Exercises grouped into types by the reviewers (all other exercises grouped according to the primary authors’ terminology)

^f^ Cool down included at least one of the following activities: walking; stretches; ice application; and/or joint movements

^g^ Education covered at least one of the following topics: pain management; coping strategies; self-training at home; pre-operative and post-operative procedures; appropriate movement patterns; knee osteoarthritis progression; the benefits of exercise and diet control; and/or physical activity

^h^ Individually indicated interventions included: strengthening exercises; sensori-motor training (including balance exercises); and/or electromyostimulation training according to the individual’s needs

^i^ Setting where exercises were performed (excluding instruction/information/optional/review sessions)

Sixteen studies identified superior outcomes in the intervention group(s) for at least one of the following: patient-reported outcomes [38, 40, 41, 43, 46, 47, 51, 57, 58, 64–67, 69], performance-based outcomes [40, 43, 44, 47, 49, 54, 57–59, 63–67, 69], and length of hospital stay [43, 54]. Most of these studies employed more than one exercise type (*n*=14). The most commonly employed exercise type was lower limb strengthening/resistance exercises (*n*=14). Commonly employed delivery approaches included using more than one delivery format (*n*=11) and personal tailoring (*n*=10).

Three studies involved a control arm and two intervention arms, allowing different exercise types/delivery approaches to be compared [40, 46, 47]. Blasco et al. [40] conducted an RCT in which the intervention groups participated in a hospital- or home-based strength and balance training intervention. In another RCT performed by the same research group [47], the intervention groups participated in strength training only or strength and balance training. Doiron-Cadrin et al. [46] conducted a pilot study in which the intervention groups participated in a multicomponent exercise program delivered in-person or via an internet-based telecommunication mobile application. All three studies identified superior outcomes in the intervention group for at least one outcome, but did not identify any significant differences between the two intervention groups at any follow-up time point.

The remaining four studies of exercise interventions did not identify any superior outcomes in favor of the intervention group [42, 50, 55, 61]. Two of these (Brown et al. [42] and Huber et al. [50]) investigated interventions that were similar to those investigated in studies that identified a significant between-group difference in favor of the intervention group for at least one outcome (Brown et al. [41] and Villadsen et al. [66, 67] respectively).

#### Views studies

Three views studies reported participants’ views of pre-operative exercise intervention components and/or delivery approaches (Table 5). In a consensus development study by Westby et al. [30], a proposed quality indicator (QI) states that patients undergoing TKR should commence an individually tailored, progressive exercise program at least 8 weeks pre-operatively and lists specific exercise components that should be included. Bin Sheeha et al. [75] conducted a qualitative study in which two participants reported that they valued receiving pre-operative exercise guidance from a physiotherapist. Conversely, three participants did not recommend pre-operative physiotherapy because they did not find it helpful or felt that the same exercises could be obtained online [75]. In a qualitative study by Sharif et al. [84], health professionals identified that web-based written information, mobile health, and remote monitoring technologies could play a role in pre-operative exercise provision, encouragement, and/or monitoring.

#### Integration of the outcomes studies and views studies

Table 5 juxtaposes the exercise intervention components and delivery approaches identified in the outcomes studies and views studies. In line with the exercise QI proposed by Westby et al. [30], 10 outcomes studies employed an individually tailored, progressive exercise program. Seven of these identified superior outcomes in the intervention group. Except for gait training, all the exercise intervention components recommended by Westby et al. [30] were included in the interventions of at least one outcomes study.

A key area of dissonance was the exercise program timing/duration. The QI proposed by Westby et al. [30] states that patients should commence an exercise program at least 8 weeks pre-operatively. In contrast, 13 outcomes studies involved programs that did not last at least 8 weeks, 11 of which identified superior outcomes in the intervention group.

### Psychological interventions

#### Outcomes studies

Two outcomes studies investigated pre-operative psychological interventions (Table 6).

**Table 6:** Psychological intervention components and delivery approaches

Category	Intervention component or delivery approach	Medina-Garzón [56]^**a**^	das Nair et al. [45]^**a**^
Component	Motivational interviewing	O	
	Psychoeducation on mood and pain		OV
	Values-based goal setting		O
	Self-management and behavioral activation		O
	Relaxation and mindful breathing		OV
	Cognitive restructuring		OV
	Post-surgical planning		O
	Signposting to relevant services		V
	Post-op reminders of the session content		V
Provider	Nurse	O	
	Psychologist		OV
Delivery mode	Single format		O
	Face-to-face		O
	Individual		OV
	Group		V
Setting	Hospital or home, according to the patient's preference		O
	Hospital		V
	Home		V
Schedule	3 sessions over a 20-day period	O	
	Up to 10 sessions delivered once or twice weekly		O
Intensity	Session length: ~40 min	O	
	Session length: ~1 h		O
Tailoring	Tailored to each individual’s needs	O	OV

*O* intervention component/delivery approach included in an intervention investigated in an outcomes study, *V* participants’ experiences/perspectives of the intervention component/delivery approach reported in a views study

^a^ Outcomes study in which a statistically significant difference in favor of the intervention group was identified for at least one outcome at one or more follow-up time points (alpha=0.05)

Medina-Garzón [56] conducted an RCT investigating a nursing intervention based on motivational interviewing. The anxiety scores at 4 weeks post-intervention were significantly lower in the intervention group compared to the control group [56]. Das Nair et al. [45] conducted a mixed methods feasibility study investigating a cognitive behavioral therapy (CBT)-based intervention. The only significant between-group difference was better patient-reported function at 6 months post-randomization in the intervention group, which Das Nair et al. [45] suggested was probably a chance finding arising from multiple comparisons.

#### Views studies

The aforementioned study by das Nair et al. [45] was the only views study that focused on a pre-operative psychological intervention (Table 6). Most participants reported finding the intervention beneficial, although some participants did not understand the intervention’s rationale and felt it had limited value. Participants attributed the benefits to various factors including specific intervention techniques and personal tailoring of the intervention. Participants’ views of the optimal setting and delivery format varied, with positives/negatives of hospital- versus home-based and group versus individual sessions being noted [45].

#### Integration of the outcomes studies and views study

Table 6 juxtaposes the psychological intervention components and delivery approaches identified in both studies of psychological interventions. The main area of agreement was that the interventions evaluated by Medina-Garzón [56] and Das Nair et al. [45] were tailored to patients’ individual needs, and participants in the qualitative component of Das Nair et al. [45] reported that they valued the personal tailoring.

### Lifestyle interventions

#### Outcomes studies

Only one outcomes study investigated a pre-operative lifestyle intervention (Table 7).

**Table 7:** Lifestyle intervention components and delivery approaches

Category	Intervention component or delivery approach	Rittharomya et al. [57]^**a**^	Aunger et al. (2020) [72]	Plenge et al. (2018) [93]	Snowden et al. (2020) [86]	Westby et al. (2018) [30]
Component	Diet control	O				
	Information on knee OA progression and the benefits of quadriceps exercise and diet control	O				
	Weight management information					V
	Weight management program					V
	Smoking cessation			V		
	Alcohol cessation			V		
	Alcohol specialist service				V	
	Alcohol consumption advice/behavior change counselling				V	
	Alcohol screening questionnaire completion				V	
	Sedentary behavior reduction		V			
	Goal setting		V			
	Environmental modifications		V^b^			
	Social support		V^b^			
	Opportunity to ask questions	O				
	Positive feedback, encouragement and compliments from a researcher	O				
	Remote monitoring by a researcher	O				
	Self-monitoring with a pedometer/health app		V			
Provider	Researcher	O	V^b^			
	Healthcare professionals in the pre-operative assessment clinic				V	
	Pre-operative assessment nurses				V	
Delivery mode	>1 format	O				
	Information/instruction sessions with a poster and DVD	O				
	Remote monitoring via telephone calls/a mobile application	O				
	Visual aids e.g. infographics or poster	O			V	
	Booklet		V^b^			
Setting	Pre-operative assessment clinic				V	
	Home and unspecified location for information/instructions	O				
Schedule	12-week program	O				
	Additional protected time in pre-operative assessment clinic				V	
	Booster session				V	
Tailoring	Tailored to each individual’s circumstances/needs		V		V	

*BMI* body mass index, *O* intervention component/delivery approach included in an intervention investigated in an outcomes study, *OA* osteoarthritis, *V* participants’ experiences/perspectives of the intervention component/delivery approach reported in a views study

^a^ Outcomes study in which a statistically significant difference in favor of the intervention group was identified for at least one outcome at one or more follow-up time points (alpha=0.05)

^b^ Finding is from a study with a mixed population and is not supported with evidence specifically for participants who met the review eligibility criteria

Rittharomya et al. [57] conducted an RCT investigating an exercise and dietary intervention. Superior outcomes in the intervention group were identified for patient-reported and performance-based outcomes during the 12-week program. Participants were not followed-up beyond the end of the program.

#### Views studies

Four views studies reported participants’ views of pre-operative lifestyle intervention components or delivery approaches (Table 7). A QI proposed by Westby et al. [30] states patients with a body mass index of 27 kg/m^2^ or over should be given weight management information and referred to a weight management program [30]. In a consensus development study by Plenge et al. [93], smoking cessation and alcohol cessation were identified as important elements of pre-operative TKR care.

The remaining two studies were mixed methods pilot and/or feasibility studies that investigated interventions aimed at reducing sedentary behavior [72] or alcohol consumption [86]. The only area of overlap was that both studies reported participants’ views of personal tailoring. Patients in the study by Aunger et al. [72] felt their sedentary behavior reduction goals were well suited to their individual circumstances, but most patients still had difficulties attaining their goals. Health professionals in the study by Snowden et al. [86] highlighted that tailoring the alcohol consumption reduction intervention and associated screening to patients’ individual needs helped keep their interactions positive.

#### Integration of the outcomes study and views studies

Table 7 juxtaposes the lifestyle intervention components and delivery approaches identified in the outcomes study and views studies. The most notable finding was that the intervention investigated by Rittharomya et al. [57] included diet control components, corresponding with the weight management QI proposed by Westby et al. [30].

### Other pre-operative interventions

#### Outcomes studies

Five outcomes studies investigated other pre-operative TKR interventions (Table 8).

**Table 8:** Other pre-operative intervention components and delivery approaches

Category	Intervention component or delivery approach	Bergin et al. [39]^**b**^	Soni et al. [61]^**b**^	Stone et al. [62]^**b**^	Walls et al. [68]^**a**^	Zhao et al. [71]^**ac**^	Bin Sheeha et al. [75]
Component	Incentive spirometry	O					
	Self-monitoring e.g. through completion of a logbook	O			O		
	Acupuncture		O				V
	Electroacupuncture					O	
	NMES				O		
	Dynamic knee extension device			O			
Provider	Physiotherapist		O				
	Acupuncturist					O	
Delivery mode	Single format		O			O	
	>1 format	O			O		
	Single instruction session (prior to unsupervised sessions)	O			O		
	Unsupervised sessions	O					
	Unsupervised sessions with written instructions				O		
	Face-to-face group sessions		O				
	Face-to-face (not specified if group or individual)					O	
Setting	Home	O			O		
	Outpatient gym		O				
Schedule	Every 2 or 6 h while awake for 1 week prior to surgery	O					
	Weekly for 4 weeks, then fortnightly for 4 weeks, then monthly until surgery		O				
	Once daily for 5 consecutive days pre-operatively					O	
	3× daily until surgery			O			
	Alternate days for 2 weeks then 5× weekly for 6 weeks				O		
Intensity	Incentive spirometry device use: 10 times per session	O					
	Acupuncture needles left in situ for 20 min		O				
	Electroacupuncture for 30 min using a device that provides a dilatational wave, 2/100 Hz, 3 mA					O	
	Dynamic knee extension device worn for up to 30 min at once			O			
	NMES session length: 20 min				O		
	NMES intensity as high as the patient can tolerate				O		
Tailoring	Frequency of incentive spirometry device use tailored to baseline incentive spirometry volume	O					

*NMES* neuromuscular electrical stimulation, *O* intervention component/delivery approach included in an intervention investigated in an outcomes study, *V* participants’ experiences/perspectives of the intervention component/delivery approach reported in a views study

^a^ Outcomes study in which a statistically significant difference in favor of the intervention group was identified for at least one outcome at one or more follow-up time points (alpha=0.05)

^b^ Outcomes study in which no statistically significant differences in favor of the intervention group were identified for any outcomes at any follow-up time points (alpha=0.05)

^c^ The control group received “placebo electroacupuncture” that involved the same provider, delivery mode and schedule as the intervention group’s electroacupuncture [71]

Superior outcomes in the intervention group were identified for cognitive function in an RCT investigating electroacupuncture [71] and the chair rise test and stair climb test in a pilot study investigating neuromuscular electrical stimulation (NMES) [68]. No significant between-group differences in favor of the intervention group were reported for RCTs investigating incentive spirometry [39], a dynamic knee extension device [62] and acupuncture plus exercise [61].

#### Views studies

The aforementioned qualitative study by Bin Sheeha et al. [75] was the only views study that addressed other pre-operative TKR interventions (Table 8). Bin Sheeha et al. [75] reported that two participants found acupuncture helpful before their surgery. However, it was unclear whether participants’ views were about acupuncture delivered solely in the pre-operative phase.

#### Integration of the outcomes studies and views study

Table 8 juxtaposes the intervention components and delivery approaches identified in the outcomes studies and the views study. The only finding of note was that one outcomes study did not identify any significant benefits of an acupuncture plus exercise intervention [61], contrasting with the perceived value of acupuncture reported by Bin Sheeha et al. [75].

## Discussion

This rapid review identified and synthesized recent literature on the content and delivery of pre-operative TKR interventions. Most of the 52 included studies focused on education or exercise interventions. Although many of the intervention components and delivery approaches identified were specific to particular intervention types, some similarities across intervention types were identified. Notably, personal tailoring was associated with improved outcomes and/or perceived as beneficial for education, exercise, psychological and lifestyle interventions. This corresponds with the emphasis on person-centered care in health policies [95]. Despite this, person-centered TKR care does not appear to be consistently implemented in clinical practice [73, 80, 96].

Only three included studies compared the effectiveness of different intervention components or delivery approaches [40, 46, 47]. The result of two RCTs suggest pre-operative TKR exercise programs are equally effective regardless of whether they include strength training only or strength plus balance training [47] and whether they are hospital or home-based [40]. A pilot RCT provided preliminary evidence that a pre-operative TKR exercise program has similar effects when it is delivered in-person or via telecommunication software [46]. However, a fully powered RCT is required to confirm this. These findings correspond with a Cochrane systematic review, which identified that the benefits of exercise programs for people with OA are not limited to specific exercise types or delivery modes [97].

The findings of the present review suggest that pre-operative TKR education should cover a comprehensive range of topics. Thirty-two topics were identified, of which rehabilitation and recovery expectations appear particularly important (Table 4). Despite this, some patients perceived education on these topics as insufficient. This review’s findings also demonstrate the importance of optimizing pre-operative education delivery. Both positives and negatives were identified for certain education delivery approaches, such as group classes. Using a combination of delivery formats could help overcome the limitations of individual formats and account for patients’ differing needs [77]. Correspondingly, employing more than one delivery format was associated with improved outcomes for education interventions, exercise interventions, a combined diet and exercise intervention and a NMES intervention.

### Relationship to previous reviews

This review provides a more comprehensive overview of pre-operative TKR education intervention components and delivery approaches than the aforementioned review by Louw et al. [19]. For example, none of the four TKR RCTs included by Louw et al. [19] employed videos, web-based or virtual reality delivery formats, all of which were identified in this review. A review by Buus et al. [16] highlighted patients value receiving pre-operative information before knee replacement and noted inadequacies in its content and delivery. The present review expands on this by also exploring health professionals’ views of pre-operative TKR education. Previous reviews have suggested that definitive evidence on the optimal content and delivery of pre-operative TKR exercise interventions is lacking [98, 99]. The present review supports this and provides information to help guide future research by summarizing the pre-operative TKR exercise intervention components and delivery approaches extracted from 23 studies (Table 5).

### Strengths and limitations

A key strength of this review is its breadth, with all types of non-pharmacological pre-operative TKR interventions being considered. The mixed methods design enabled a more in-depth insight to be gained than would have been achieved through a purely quantitative or qualitative design [100]. Systematic approaches were used during all stages of the review. However, the rapid review methodology involved streamlining various aspects of standard systematic review methods. For example, the searches were limited to electronic databases and reference lists of eligible studies, increasing the likelihood that relevant studies may have been missed [101].

Outcomes studies were dichotomized based on whether they identified a statistically significant difference in favor of the intervention group for at least one outcome. This was considered appropriate given that the review aimed to provide an overview of intervention components and delivery approaches rather than definitive evidence about their effectiveness. However, it involved relying on an arbitrary threshold (alpha=0.05) and statistically significant improvements are not necessarily clinically relevant [102]. This is an important limitation because previous research has suggested that the effects of pre-operative TKR interventions may not be large enough to be clinically important [103].

No primary study authors were contacted despite the intervention reporting of some studies being poor. Consequently, relevant information about intervention components and delivery approaches may have been missed. Where possible, the primary study authors’ terminology was used to describe intervention components and delivery approaches. This led to some inconsistency in the coding. For example, stretches were considered part of the cool down in some studies but listed separately in others. This review’s findings also need to be interpreted in light of the limitations of the included studies. The MMAT ratings suggested that most of the included qualitative studies are high quality, whereas all the other included studies present at least some quality issues.

### Implications for clinical practice and future research

A key implication of this review for clinical practice and future research is that personal tailoring and employing more than one delivery format appear to be valuable design elements for most pre-operative TKR intervention types. In addition, this review identified preliminary evidence that including balance training and hospital versus home delivery are not essential design elements for pre-operative TKR exercise interventions. The latter is particularly relevant due to the lower costs associated with home-based programs. Furthermore, the COVID-19 pandemic has highlighted the need for remote models of care [104, 105]. Using digital tools to deliver TKR care remotely offers multiple potential benefits, such as improved service efficiency and greater patient engagement [84, 104]. Conversely, this review identified few studies that investigated the effectiveness of digital tools. This review also identified a paucity of studies focused on pre-operative psychological or lifestyle interventions, despite the negative impact of psychological distress and unhealthy lifestyle behaviors on TKR outcomes [106, 107]. This highlights the need for future research investigating pre-operative TKR interventions that incorporate digital tools, provide psychological support and/or address lifestyle behaviors.

Another clinically relevant finding is that some patients perceive pre-operative TKR education as insufficient. Potential strategies for addressing this include covering a comprehensive range of topics and ensuring that rehabilitation and recovery expectations are adequately addressed. The detailed tables of intervention components and delivery approaches developed in this review provide a resource for informing the design of pre-operative TKR interventions for clinical practice and future research (Tables 4, 5, 6, 7, and 8). In particular, the intervention components and delivery approaches identified in multiple supposedly effective interventions warrant further investigation [108]. Other important aspects to address are the areas of dissonance between the outcomes studies and views studies, such as the exercise program duration.

## Conclusions

This review comprehensively synthesized literature on the content and delivery of pre-operative TKR interventions. The findings demonstrate that definitive evidence to guide the design of pre-operative TKR interventions is lacking. Personal tailoring and employing more than one delivery format appear to be valuable design elements for most pre-operative TKR intervention types. Preliminary evidence was identified that suggests including balance training and hospital versus home delivery are not critical design elements for pre-operative TKR exercise interventions. Another key finding was that covering a comprehensive range of education topics, including rehabilitation and recovery expectations, could help address the insufficiencies in pre-operative TKR education perceived by some patients.

## Supplementary Information

**Additional file 1.** PRISMA checklist. Completed PRISMA checklist (Supplementary Table 1).

**Additional file 2.** Database search strategies. Search strategies employed for all electronic databases searched.

**Additional file 3.** Data items. Data items extracted for outcomes studies (Supplementary Table 2) and views studies (Supplementary Table 3).

**Additional file 4.** Outcomes studies’ characteristics and results. Characteristics and results of the included outcomes studies (Supplementary Table 4).

**Additional file 5.** Views studies’ characteristics and findings. Characteristics and findings of the included views studies (Supplementary Table 5).

## Data Availability

The datasets used and/or analyzed during the current study are available from the corresponding author on reasonable request.

## Abbreviations

MMAT: Mixed Methods Appraisal Tool
NMES: Neuromuscular electrical stimulation
OA: Osteoarthritis
PI: Principal investigator
QI: Quality indicator
THR: Total hip replacement
TKR: Total knee replacement
