# Supplementary material for: Content and delivery of pre-operative interventions for patients undergoing total knee replacement: a rapid review
Source: Syst Rev. 2022 Sep 2;11:184. doi: 10.1186/s13643-022-02019-x (PMC9436722; doi:10.1186/s13643-022-02019-x)
Supplement: Supplementary file 2 — Additional file 2. Database search strategies. Search strategies employed for all electronic databases searched. [file 13643_2022_2019_MOESM2_ESM.docx]

**Content and delivery of pre-operative interventions for patients**

**undergoing total knee replacement: a rapid review**

**Additional File 2: Database search strategies**

The search strategies presented below are for the initial database searches conducted on 11^th^ September 2019. All the searches were subsequently updated to 31st December 2020.

# Medline (Ovid)

1 Arthroplasty, Replacement, Knee/ (22063)

2 Knee Prosthesis/ (11206)

3 (TKA or TKR).tw,kw. (11739)

4 1 or 2 or 3 (30266)

5 Knee/ (13711)

6 Knee Joint/ (52242)

7 Osteoarthritis, Knee/ (18298)

8 knee?.tw,kw. (139011)

9 5 or 6 or 7 or 8 (154624)

10 Arthroplasty, Replacement/ (5872)

11 (arthroplast* or replace*).tw,kw. (425753)

12 10 or 11 (427000)

13 9 and 12 (34513)

14 4 or 13 (39999)

15 Preoperative Care/ (60281)

16 Preoperative Period/ (6405)

17 (preop* or pre-op* or presurg* or pre-surg* or ?prehab* or ?pre-hab* or teleprehab* or tele-prehab* or prepar*).tw,kw. (1184603)

18 15 or 16 or 17 (1211482)

19 Patient Education as Topic/ (82785)

20 Health Education/ (59265)

21 exp Consumer Health Information/ (8221)

22 Teach-Back Communication/ (28)

23 exp Educational Technology/ (107154)

24 Patient Education Handout/ (5007)

25 ((health* or educat* or inform* or knowledge or teach*) adj3 (class* or group? or program* or school? or booklet? or leaflet? or DVD? or YouTube or video? or website? or "web platform" or "web platforms" or "web page" or "web pages" or web-page? or microsite? or app? or application? or multimedia)).tw,kw. (255384)

26 ((patient? adj2 educat*) or psychoeducat* or psycho-educat*).tw,kw. (33748)

27 exp Exercise/ (182510)

28 exp Exercise Therapy/ (47249)

29 exp Exercise Movement Techniques/ (7683)

30 Rehabilitation/ (17926)

31 Hospitals, Rehabilitation/ (34)

32 Rehabilitation Centers/ (8017)

33 Rehabilitation Nursing/ (1397)

34 Rehabilitation Research/ (129)

35 Recreation Therapy/ (114)

36 Telerehabilitation/ (277)

37 "Physical and Rehabilitation Medicine"/ (3122)

38 Physical Therapy Modalities/ (35459)

39 Occupational Therapy/ (12741)

40 Hydrotherapy/ (2511)

41 ((joint? or knee? or motor or physical* or cardio* or strength* or propriocept* or balance or neuromuscular or aerobic or weight or stretch* or resistance or endurance or aqua*) adj2 (school* or train* or activit* or fit* or program* or class* or therap*)).tw,kw. (243474)

42 (?rehab* or ?prehab* or ?pre-hab* or telerehab* or tele-rehab* or teleprehab* or tele-prehab or ?exercis* or ?physiotherap* or hydrotherap* or "occupational therapy" or swim* or cycl* or bik* or self-management or "self management").tw,kw. (1572703)

43 exp Psychotherapy/ (189129)

44 exp Mind-Body Therapies/ (48979)

45 Counseling/ (34516)

46 Distance Counseling/ (36)

47 exp Directive Counseling/ (3807)

48 (psychotherap* or "guided imagery" or CBT or relax* or hypnosis or "motivational interviewing" or mindfulness or counsel* or "pain coping skills training").tw,kw. (328178)

49 ((psychologic* or behavio?r* or cognitive or emotion* or mind) adj2 (intervention* or technique* or therap* or treat* or prepar* or restructur* or reframe* or distract*)).tw,kw. (73859)

50 exp Health Promotion/ (73339)

51 exp Diet Therapy/ (52287)

52 exp Life Style/ (88041)

53 Alcohol Abstinence/ (557)

54 Smoking Cessation/ (27171)

55 Smoking Reduction/ (28)

56 "Tobacco Use Cessation"/ (1094)

57 ("physical activity" or "weight loss" or "weight reduction" or diet*).tw,kw. (688013)

58 ((behavio?r* or lifestyle* or health*) adj2 (chang* or modif* or motivat* or promot* or educat* or inform* or teach*)).tw,kw. (232005)

59 ((smoking or tobacco or alcohol) adj2 (cessat* or reduc* or stop* or quit*)).tw,kw. (42910)

60 Nutrition Therapy/ (2086)

61 exp Dietary Supplements/ (70536)

62 Functional Food/ (1668)

63 exp Micronutrients/ (636727)

64 exp Minerals/ (159185)

65 ((nutrition* adj2 supplement*) or probiotic* or prebiotic* or synbiotic* or "functional food" or nutraceutical* or nutrient* or glucosamine or chondroitin or curcumin or "fish oil" or "fish oils" or "omega 3" or vitamin* or mineral* or "trace element" or "trace elements" or flavonoid* or (hydroly* adj2 collagen)).tw,kw. (623531)

66 Transcutaneous Electric Nerve Stimulation/ (4486)

67 (electrotherap* or "transcutaneous electrical nerve stimulation" or TENS).tw,kw. (17392)

68 exp Therapy, Soft Tissue/ (6707)

69 Trigger Points/ (474)

70 (massag* or "soft tissue therapy" or "trigger point" or "trigger points").tw,kw. (12297)

71 exp Orthotic Devices/ (12585)

72 (orthotic* or orthos* or insole* or "arch support" or (knee adj2 brace*)).tw,kw. (24743)

73 Acupuncture/ (1626)

74 exp Acupuncture Therapy/ (23292)

75 (acupuncture or acupressure or "dry needling").tw,kw. (21980)

76 Rehabilitation, Vocational/ (9329)

77 ((occupation* or vocation*) adj2 rehab*).tw,kw. (3749)

78 19 or 20 or 21 or 22 or 23 or 24 or 25 or 26 or 27 or 28 or 29 or 30 or 31 or 32 or 33 or 34 or 35 or 36 or 37 or 38 or 39 or 40 or 41 or 42 or 43 or 44 or 45 or 46 or 47 or 48 or 49 or 50 or 51 or 52 or 53 or 54 or 55 or 56 or 57 or 58 or 59 or 60 or 61 or 62 or 63 or 64 or 65 or 66 or 67 or 68 or 69 or 70 or 71 or 72 or 73 or 74 or 75 or 76 or 77 (4522618)

79 14 and 18 and 78 (1629)

80 exp Animals/ (22584094)

81 Humans/ (17966945)

82 80 not 81 (4617149)

83 79 not 82 (1616)

84 limit 83 to (english language and yr="2009 -Current") (1092)

# Embase (Ovid)

1 exp knee arthroplasty/ (25198)

2 exp knee prosthesis/ (8393)

3 (TKA or TKR).tw,kw. (15077)

4 1 or 2 or 3 (36587)

5 knee/ (49542)

6 knee arthritis/ (3154)

7 knee osteoarthritis/ (28779)

8 knee pain/ (15736)

9 knee?.tw,kw. (156466)

10 5 or 6 or 7 or 8 or 9 (167696)

11 arthroplasty/ (15518)

12 replacement arthroplasty/ (1152)

13 total arthroplasty/ (754)

14 (arthroplast* or replace*).tw,kw. (458936)

15 11 or 12 or 13 or 14 (463474)

16 10 and 15 (42423)

17 4 or 16 (51128)

18 preoperative period/ (49610)

19 preoperative education/ (501)

20 preoperative care/ (28649)

21 preoperative treatment/ (10396)

22 (preop* or pre-op* or presurg* or pre-surg* or ?prehab* or ?pre-hab* or teleprehab* or tele-prehab* or prepar*).tw,kw. (1150599)

23 18 or 19 or 20 or 21 or 22 (1177889)

24 health education/ (67431)

25 patient education/ (92268)

26 preoperative education/ (501)

27 psychoeducation/ (7367)

28 educational technology/ (2969)

29 health literacy/ (9852)

30 ((health* or educat* or inform* or knowledge or teach*) adj3 (class* or group? or program* or school? or booklet? or leaflet? or DVD? or YouTube or video? or website? or "web platform" or "web platforms" or "web page" or "web pages" or web-page? or microsite? or app? or application? or multimedia)).tw,kw. (291538)

31 ((patient? adj2 educat*) or psychoeducat* or psycho-educat*).tw,kw. (51288)

32 exp exercise/ (271748)

33 exp kinesiotherapy/ (64422)

34 rehabilitation/ (57520)

35 community based rehabilitation/ (729)

36 geriatric rehabilitation/ (868)

37 home rehabilitation/ (631)

38 rehabilitation care/ (15503)

39 rehabilitation center/ (11090)

40 functional training/ (1121)

41 recreational therapy/ (511)

42 telerehabilitation/ (626)

43 rehabilitation medicine/ (8725)

44 rehabilitation nursing/ (1370)

45 rehabilitation patient/ (1134)

46 rehabilitation research/ (917)

47 physiotherapy/ (70574)

48 occupational therapy/ (16351)

49 hydrotherapy/ (2470)

50 ((joint? or knee? or motor or physical* or cardio* or strength* or propriocept* or balance or neuromuscular or aerobic or weight or stretch* or resistance or endurance or aqua*) adj2 (school* or train* or activit* or fit* or program* or class* or therap*)).tw,kw. (302632)

51 (?rehab* or ?prehab* or ?pre-hab* or telerehab* or tele-rehab* or teleprehab* or tele-prehab or ?exercis* or ?physiotherap* or hydrotherap* or "occupational therapy" or swim* or cycl* or bik* or self-management or "self management").tw,kw. (1656013)

52 exp psychotherapy/ (180922)

53 counseling/ (52184)

54 patient guidance/ (1215)

55 directive counseling/ (836)

56 e-counseling/ (161)

57 motivational interviewing/ (4415)

58 patient counseling/ (39786)

59 peer counseling/ (553)

60 (psychotherap* or "guided imagery" or CBT or relax* or hypnosis or "motivational interviewing" or mindfulness or counsel* or "pain coping skills training").tw,kw. (329822)

61 ((psychologic* or behavio?r* or cognitive or emotion* or mind) adj2 (intervention* or technique* or therap* or treat* or prepar* or restructur* or reframe* or distract*)).tw,kw. (94898)

62 health promotion/ (82448)

63 exp lifestyle/ (114190)

64 diet therapy/ (42225)

65 diet restriction/ (85270)

66 exp low calorie diet/ (471)

67 low fat diet/ (9722)

68 caloric restriction/ (11571)

69 nutritional counseling/ (2469)

70 alcohol abstinence/ (5985)

71 smoking cessation/ (52928)

72 smoking reduction/ (149)

73 ("physical activity" or "weight loss" or "weight reduction" or diet*).tw,kw. (742277)

74 ((behavio?r* or lifestyle* or health*) adj2 (chang* or modif* or motivat* or promot* or educat* or inform* or teach*)).tw,kw. (286920)

75 ((smoking or tobacco or alcohol) adj2 (cessat* or reduc* or stop* or quit*)).tw,kw. (51680)

76 diet supplementation/ (78109)

77 functional food/ (3950)

78 mineral supplementation/ (1509)

79 vitamin supplementation/ (31009)

80 ((nutrition* adj2 supplement*) or probiotic* or prebiotic* or synbiotic* or "functional food" or nutraceutical* or nutrient* or glucosamine or chondroitin or curcumin or "fish oil" or "fish oils" or "omega 3" or vitamin* or mineral* or "trace element" or "trace elements" or flavonoid* or (hydroly* adj2 collagen)).tw,kw. (656277)

81 transcutaneous electrical nerve stimulation/ (1510)

82 (electrotherap* or "transcutaneous electrical nerve stimulation" or TENS).tw,kw. (15658)

83 soft tissue therapy/ (120)

84 massage/ (12085)

85 trigger point/ (2205)

86 (massag* or "soft tissue therapy" or "trigger point" or "trigger points").tw,kw. (13922)

87 orthotics/ (2671)

88 knee brace/ (568)

89 (orthotic* or orthos* or insole* or "arch support" or (knee adj2 brace*)).tw,kw. (28153)

90 exp acupuncture/ (36848)

91 (acupuncture or acupressure or "dry needling").tw,kw. (25792)

92 vocational rehabilitation/ (5042)

93 ((occupation* or vocation*) adj2 rehab*).tw,kw. (3392)

94 24 or 25 or 26 or 27 or 28 or 29 or 30 or 31 or 32 or 33 or 34 or 35 or 36 or 37 or 38 or 39 or 40 or 41 or 42 or 43 or 44 or 45 or 46 or 47 or 48 or 49 or 50 or 51 or 52 or 53 or 54 or 55 or 56 or 57 or 58 or 59 or 60 or 61 or 62 or 63 or 64 or 65 or 66 or 67 or 68 or 69 or 70 or 71 or 72 or 73 or 74 or 75 or 76 or 77 or 78 or 79 or 80 or 81 or 82 or 83 or 84 or 85 or 86 or 87 or 88 or 89 or 90 or 91 or 92 or 93 (4193496)

95 17 and 23 and 94 (2418)

96 exp animal/ (18670046)

97 exp human/ (15771110)

98 96 not 97 (2898936)

99 95 not 98 (2404)

100 limit 99 to (english language and yr="2009 -Current") (1850)

# PsycINFO (Ovid)

1 (TKA or TKR).tw. (170)

2 knee/ (939)

3 knee?.tw. (3452)

4 2 or 3 (3471)

5 (arthroplast* or replace*).tw. (19930)

6 4 and 5 (473)

7 1 or 6 (483)

8 (preop* or pre-op* or presurg* or pre-surg* or ?prehab* or ?pre-hab* or teleprehab* or tele-prehab* or prepar*).tw. (66823)

9 health education/ (8501)

10 client education/ (2167)

11 health information/ (2062)

12 digital information/ (76)

13 psychoeducation/ (3321)

14 health literacy/ (2682)

15 ((health* or educat* or inform* or knowledge or teach*) adj3 (class* or group? or program* or school? or booklet? or leaflet? or DVD? or YouTube or video? or website? or "web platform" or "web platforms" or "web page" or "web pages" or web-page? or microsite? or app? or application? or multimedia)).tw. (129848)

16 ((patient? adj2 educat*) or psychoeducat* or psycho-educat*).tw. (12512)

17 exp exercise/ (19525)

18 rehabilitation/ (12631)

19 rehabilitation centers/ (270)

20 recreation therapy/ (231)

21 telerehabilitation/ (131)

22 movement therapy/ (958)

23 physical therapy/ (2124)

24 occupational therapy/ (3691)

25 hydrotherapy/ (25)

26 ((joint? or knee? or motor or physical* or cardio* or strength* or propriocept* or balance or neuromuscular or aerobic or weight or stretch* or resistance or endurance or aqua*) adj2 (school* or train* or activit* or fit* or program* or class* or therap*)).tw. (51813)

27 (?rehab* or ?prehab* or ?pre-hab* or telerehab* or tele-rehab* or teleprehab* or tele-prehab or ?exercis* or ?physiotherap* or hydrotherap* or "occupational therapy" or swim* or cycl* or bik* or "self-management" or "self management").tw. (142318)

28 exp psychotherapy/ (102554)

29 mind body therapy/ (177)

30 exp cognitive techniques/ (6634)

31 exp cognitive behavior therapy/ (19861)

32 counseling/ (12303)

33 group counseling/ (1457)

34 peer counseling/ (412)

35 exp hypnosis/ (2836)

36 online therapy/ (2675)

37 anxiety management/ (403)

38 stress management/ (2764)

39 exp relaxation therapy/ (552)

40 muscle relaxation/ (231)

41 (psychotherap* or "guided imagery" or CBT or relax* or hypnosis or "motivational interviewing" or mindfulness or counsel* or "pain coping skills training").tw. (142948)

42 ((psychologic* or behavio?r* or cognitive or emotion* or mind) adj2 (intervention* or technique* or therap* or treat* or prepar* or restructur* or reframe* or distract*)).tw. (69442)

43 health promotion/ (20809)

44 exp behavior modification/ (15811)

45 exp lifestyle/ (8394)

46 physical activity/ (17517)

47 weight control/ (3239)

48 weight loss/ (2834)

49 diets/ (8791)

50 dietary restraint/ (1244)

51 exp alcohol treatment/ (3919)

52 sobriety/ (976)

53 smoking cessation/ (10299)

54 ("physical activity" or "weight loss" or "weight reduction" or diet*).tw. (61027)

55 ((behavio?r* or lifestyle* or health*) adj2 (chang* or modif* or motivat* or promot* or educat* or inform* or teach*)).tw. (92052)

56 ((smoking or tobacco or alcohol) adj2 (cessat* or reduc* or stop* or quit*)).tw. (16603)

57 dietary supplements/ (1754)

58 exp vitamins/ (3363)

59 ((nutrition* adj2 supplement*) or probiotic* or prebiotic* or synbiotic* or "functional food" or nutraceutical* or nutrient* or glucosamine or chondroitin or curcumin or "fish oil" or "fish oils" or "omega 3" or vitamin* or mineral* or "trace element" or "trace elements" or flavonoid* or (hydroly* adj2 collagen)).tw. (13584)

60 (electrotherap* or "transcutaneous electrical nerve stimulation" or TENS).tw. (1459)

61 massage/ (441)

62 (massag* or "soft tissue therapy" or "trigger point" or "trigger points").tw. (1443)

63 (orthotic* or orthos* or insole* or "arch support" or (knee adj2 brace*)).tw. (1574)

64 acupuncture/ (1022)

65 (acupuncture or acupressure or "dry needling").tw. (1504)

66 exp vocational rehabilitation/ (3276)

67 occupational guidance/ (3149)

68 ((occupation* or vocation*) adj2 rehab*).tw. (2288)

69 9 or 10 or 11 or 12 or 13 or 14 or 15 or 16 or 17 or 18 or 19 or 20 or 21 or 22 or 23 or 24 or 25 or 26 or 27 or 28 or 29 or 30 or 31 or 32 or 33 or 34 or 35 or 36 or 37 or 38 or 39 or 40 or 41 or 42 or 43 or 44 or 45 or 46 or 47 or 48 or 49 or 50 or 51 or 52 or 53 or 54 or 55 or 56 or 57 or 58 or 59 or 60 or 61 or 62 or 63 or 64 or 65 or 66 or 67 or 68 (637241)

70 7 and 8 and 69 (69)

71 limit 70 to (human and english language and yr="2009 -Current") (50)

# CINAHL (EBSCOhost)

| **#** | **Query** | **Limiters/Expanders** | **Results** |
| --- | --- | --- | --- |
| S97 | S96 | Limiters - English Language; Published Date: 20090101-20190931 Search modes - Boolean/Phrase | 1,014 |
| S96 | S92 NOT S95 | Search modes - Boolean/Phrase | 1,281 |
| S95 | S93 NOT S94 | Search modes - Boolean/Phrase | 75,189 |
| S94 | (MH "Human") | Search modes - Boolean/Phrase | 1,968,486 |
| S93 | (MH "Animals+") | Search modes - Boolean/Phrase | 83,568 |
| S92 | S13 AND S17 AND S91 | Search modes - Boolean/Phrase | 1,285 |
| S91 | S18 OR S19 OR S20 OR S21 OR S22 OR S23 OR S24 OR S25 OR S26 OR S27 OR S28 OR S29 OR S30 OR S31 OR S32 OR S33 OR S34 OR S35 OR S36 OR S37 OR S38 OR S39 OR S40 OR S41 OR S42 OR S43 OR S44 OR S45 OR S46 OR S47 OR S48 OR S49 OR S50 OR S51 OR S52 OR S53 OR S54 OR S55 OR S56 OR S57 OR S58 OR S59 OR S60 OR S61 OR S62 OR S63 OR S64 OR S65 OR S66 OR S67 OR S68 OR S69 OR S70 OR S71 OR S72 OR S73 OR S74 OR S75 OR S76 OR S77 OR S78 OR S79 OR S80 OR S81 OR S82 OR S83 OR S84 OR S85 OR S86 OR S87 OR S88 OR S89 OR S90 | Search modes - Boolean/Phrase | 1,437,068 |
| S90 | TI ( (occupation* or vocation*) n1 rehab* ) OR AB ( (occupation* or vocation*) n1 rehab* ) | Search modes - Boolean/Phrase | 2,531 |
| S89 | (MH "Rehabilitation, Vocational") | Search modes - Boolean/Phrase | 5,421 |
| S88 | TI ( acupuncture or acupressure or "dry needling" ) OR AB ( acupuncture or acupressure or "dry needling" ) | Search modes - Boolean/Phrase | 11,497 |
| S87 | (MH "Dry Needling") | Search modes - Boolean/Phrase | 189 |
| S86 | (MH "Acupuncture+") | Search modes - Boolean/Phrase | 14,315 |
| S85 | TI ( orthotic* or orthos* or insole* or "arch support" or (knee n1 brace*) ) OR AB ( orthotic* or orthos* or insole* or "arch support" or (knee n1 brace*) ) | Search modes - Boolean/Phrase | 8,041 |
| S84 | (MH "Orthoses+") | Search modes - Boolean/Phrase | 9,122 |
| S83 | TI ( massag* or "soft tissue therapy" or "trigger point" or "trigger points" ) OR AB ( massag* or "soft tissue therapy" or "trigger point" or "trigger points" ) | Search modes - Boolean/Phrase | 10,659 |
| S82 | (MH "Trigger Point") | Search modes - Boolean/Phrase | 1,237 |
| S81 | (MH "Massage+") | Search modes - Boolean/Phrase | 13,936 |
| S80 | TI ( electrotherap* or "transcutaneous electrical nerve stimulation" or TENS ) OR AB ( electrotherap* or "transcutaneous electrical nerve stimulation" or TENS ) | Search modes - Boolean/Phrase | 238,919 |
| S79 | (MH "Transcutaneous Electric Nerve Stimulation") | Search modes - Boolean/Phrase | 2,005 |
| S78 | TI ( (nutrition* n1 supplement*) or probiotic* or prebiotic* or synbiotic* or "functional food" or nutraceutical* or nutrient* or glucosamine or chondroitin or curcumin or "fish oil" or "fish oils" or "omega 3" or vitamin* or mineral* or "trace element" or "trace elements" or flavonoid* or (hydroly* n1 collagen) ) OR AB ( (nutrition* n1 supplement*) or probiotic* or prebiotic* or synbiotic* or "functional food" or nutraceutical* or nutrient* or glucosamine or chondroitin or curcumin or "fish oil" or "fish oils" or "omega 3" or vitamin* or mineral* or "trace element" or "trace elements" or flavonoid* or (hydroly* n1 collagen) ) | Search modes - Boolean/Phrase | 82,749 |
| S77 | (MH "Minerals+") | Search modes - Boolean/Phrase | 9,781 |
| S76 | (MH "Vitamins+") | Search modes - Boolean/Phrase | 46,693 |
| S75 | (MH "Functional Food") | Search modes - Boolean/Phrase | 2,514 |
| S74 | (MH "Dietary Supplements+") | Search modes - Boolean/Phrase | 25,640 |
| S73 | TI ( (smoking or tobacco or alcohol) n1 (cessat* or reduc* or stop* or quit*) ) OR AB ( (smoking or tobacco or alcohol) n1 (cessat* or reduc* or stop* or quit*) ) | Search modes - Boolean/Phrase | 20,423 |
| S72 | TI ( (behavio#r* or lifestyle* or health*) n1 (chang* or modif* or motivat* or promot* or educat* or inform* or teach*) ) OR AB ( (behavio#r* or lifestyle* or health*) n1 (chang* or modif* or motivat* or promot* or educat* or inform* or teach*) ) | Search modes - Boolean/Phrase | 113,484 |
| S71 | TI ( "physical activity" or "weight loss" or "weight reduction" or diet* ) OR AB ( "physical activity" or "weight loss" or "weight reduction" or diet* ) | Search modes - Boolean/Phrase | 162,092 |
| S70 | (MH "Smoking Cessation Programs") | Search modes - Boolean/Phrase | 2,115 |
| S69 | (MH "Smoking Cessation") | Search modes - Boolean/Phrase | 18,502 |
| S68 | (MH "Alcohol Rehabilitation Programs") | Search modes - Boolean/Phrase | 1,763 |
| S67 | (MH "Nutritional Counseling") | Search modes - Boolean/Phrase | 2,088 |
| S66 | (MH "Restricted Diet") | Search modes - Boolean/Phrase | 2,277 |
| S65 | (MH "Diet, Low Carbohydrate") | Search modes - Boolean/Phrase | 950 |
| S64 | (MH "Diet, Fat-Restricted") | Search modes - Boolean/Phrase | 2,192 |
| S63 | (MH "Diet, Reducing") | Search modes - Boolean/Phrase | 3,924 |
| S62 | (MH "Life Style+") | Search modes - Boolean/Phrase | 196,359 |
| S61 | (MH "Health Promotion") | Search modes - Boolean/Phrase | 57,641 |
| S60 | TI ( (psychologic* or behavio#r* or cognitive or emotion* or mind) n1 (intervention* or technique* or therap* or treat* or prepar* or restructur* or reframe* or distract*) ) OR AB ( (psychologic* or behavio#r* or cognitive or emotion* or mind) n1 (intervention* or technique* or therap* or treat* or prepar* or restructur* or reframe* or distract*) ) | Search modes - Boolean/Phrase | 33,357 |
| S59 | TI ( psychotherap* or "guided imagery" or CBT or relax* or hypnosis or "motivational interviewing" or mindfulness or counsel* or "pain coping skills training") OR AB ( psychotherap* or "guided imagery" or CBT or relax* or hypnosis or "motivational interviewing" or mindfulness or counsel* or "pain coping skills training") | Search modes - Boolean/Phrase | 85,656 |
| S58 | (MH "Motivational Interviewing") | Search modes - Boolean/Phrase | 2,886 |
| S57 | (MH "Peer Counseling") | Search modes - Boolean/Phrase | 946 |
| S56 | (MH "Counseling") | Search modes - Boolean/Phrase | 25,608 |
| S55 | (MH "Hypnosis+") | Search modes - Boolean/Phrase | 2,665 |
| S54 | (MH "Mind Body Techniques+") | Search modes - Boolean/Phrase | 36,676 |
| S53 | (MH "Relaxation Techniques+") | Search modes - Boolean/Phrase | 10,907 |
| S52 | (MH "Psychotherapy, Group") | Search modes - Boolean/Phrase | 4,641 |
| S51 | (MH "Cognitive Therapy+") | Search modes - Boolean/Phrase | 20,938 |
| S50 | (MH "Behavior Therapy") | Search modes - Boolean/Phrase | 9,786 |
| S49 | (MH "Psychotherapy+") | Search modes - Boolean/Phrase | 166,983 |
| S48 | TI ( (rehab* or prehab* or pre-hab* or telerehab* or tele-rehab* or teleprehab* or tele-prehab or exercis* or physiotherap* or hydrotherap* or "occupational therapy" or swim* or cycl* or bik* or "self-management" or "self management") ) OR AB ( (rehab* or prehab* or pre-hab* or telerehab* or tele-rehab* or teleprehab* or tele-prehab or exercis* or physiotherap* or hydrotherap* or "occupational therapy" or swim* or cycl* or bik* or self-management or "self management") ) | Search modes - Boolean/Phrase | 284,913 |
| S47 | TI ( (joint# or knee# or motor or physical* or cardio* or strength* or propriocept* or balance or neuromuscular or aerobic or weight or stretch* or resistance or endurance or aqua*) n1 (school* or train* or activit* or fit* or program* or class* or therap*) ) OR AB ( (joint# or knee# or motor or physical* or cardio* or strength* or propriocept* or balance or neuromuscular or aerobic or weight or stretch* or resistance or endurance or aqua*) n1 (school* or train* or activit* or fit* or program* or class* or therap*) ) | Search modes - Boolean/Phrase | 106,503 |
| S46 | (MH "Hydrotherapy+") | Search modes - Boolean/Phrase | 5,861 |
| S45 | (MH "Occupational Therapy") | Search modes - Boolean/Phrase | 18,446 |
| S44 | (MH "Physical Therapy") | Search modes - Boolean/Phrase | 31,370 |
| S43 | (MH "Telerehabilitation") | Search modes - Boolean/Phrase | 120 |
| S42 | (MH "Recreational Therapy") | Search modes - Boolean/Phrase | 1,349 |
| S41 | (MH "Research, Rehabilitation") | Search modes - Boolean/Phrase | 1,050 |
| S40 | (MH "Rehabilitation Nursing") | Search modes - Boolean/Phrase | 1,498 |
| S39 | (MH "Functional Training") | Search modes - Boolean/Phrase | 965 |
| S38 | (MH "Rehabilitation Patients") | Search modes - Boolean/Phrase | 3,007 |
| S37 | (MH "Rehabilitation, Geriatric") | Search modes - Boolean/Phrase | 2,799 |
| S36 | (MH "Rehabilitation, Community-Based") | Search modes - Boolean/Phrase | 909 |
| S35 | (MH "Home Rehabilitation+") | Search modes - Boolean/Phrase | 1,893 |
| S34 | (MH "Rehabilitation Centers") | Search modes - Boolean/Phrase | 7,038 |
| S33 | (MH "Rehabilitation") | Search modes - Boolean/Phrase | 14,759 |
| S32 | (MH "Walking+") | Search modes - Boolean/Phrase | 27,951 |
| S31 | (MH "Upper Extremity Exercises+") | Search modes - Boolean/Phrase | 344 |
| S30 | (MH "Muscle Strengthening+") | Search modes - Boolean/Phrase | 20,125 |
| S29 | (MH "Therapeutic Exercise+") | Search modes - Boolean/Phrase | 49,447 |
| S28 | (MH "Aerobic Exercises+") | Search modes - Boolean/Phrase | 38,423 |
| S27 | (MH "Prehabilitation") | Search modes - Boolean/Phrase | 90 |
| S26 | (MH "Exercise+") | Search modes - Boolean/Phrase | 100,946 |
| S25 | TI ( (patient# n1 educat*) or psychoeducat* or psycho-educat* ) OR AB ( (patient# n1 educat*) or psychoeducat* or psycho-educat* ) | Search modes - Boolean/Phrase | 19,363 |
| S24 | TI ( (health* or educat* or inform* or knowledge or teach*) n2 (class* or group# or program* or school# or booklet# or leaflet# or DVD# or YouTube or video# or website# or "web platform" or "web platforms" or "web page" or "web pages" or web-page# or microsite# or app# or application# or multimedia) ) OR AB ( (health* or educat* or inform* or knowledge or teach*) n2 (class* or group# or program* or school# or booklet# or leaflet# or DVD# or YouTube or video# or website# or "web platform" or "web platforms" or "web page" or "web pages" or web-page# or microsite# or app# or application# or multimedia) ) | Search modes - Boolean/Phrase | 113,365 |
| S23 | (MH "Health Literacy") | Search modes - Boolean/Phrase | 3,538 |
| S22 | (MH "Educational Technology") | Search modes - Boolean/Phrase | 1,804 |
| S21 | (MH "Psychoeducation") | Search modes - Boolean/Phrase | 2,821 |
| S20 | (MH "Preoperative Education") | Search modes - Boolean/Phrase | 1,421 |
| S19 | (MH "Health Education") | Search modes - Boolean/Phrase | 23,086 |
| S18 | (MH "Patient Education") | Search modes - Boolean/Phrase | 59,103 |
| S17 | S14 OR S15 OR S16 | Search modes - Boolean/Phrase | 150,619 |
| S16 | TI ( preop* or pre-op* or presurg* or pre-surg* or prehab* or pre-hab* or teleprehab* or tele-prehab* or prepar*) OR AB (preop* or pre-op* or presurg* or pre-surg* or prehab* or pre-hab* or teleprehab* or tele-prehab* or prepar*) | Search modes - Boolean/Phrase | 138,468 |
| S15 | (MH "Preoperative Care+") | Search modes - Boolean/Phrase | 19,525 |
| S14 | (MH "Preoperative Period") | Search modes - Boolean/Phrase | 4,842 |
| S13 | S3 OR S12 | Search modes - Boolean/Phrase | 19,081 |
| S12 | S8 AND S11 | Search modes - Boolean/Phrase | 15,797 |
| S11 | S9 OR S10 | Search modes - Boolean/Phrase | 75,409 |
| S10 | TI (arthroplast* OR replace*) OR AB (arthroplast* OR replace*) | Search modes - Boolean/Phrase | 74,320 |
| S9 | (MH "Arthroplasty, Replacement") | Search modes - Boolean/Phrase | 3,613 |
| S8 | S4 OR S5 OR S6 OR S7 | Search modes - Boolean/Phrase | 61,090 |
| S7 | TI knee# OR AB knee# | Search modes - Boolean/Phrase | 55,099 |
| S6 | (MH "Osteoarthritis, Knee") | Search modes - Boolean/Phrase | 10,366 |
| S5 | (MH "Knee Joint") | Search modes - Boolean/Phrase | 15,719 |
| S4 | (MH "Knee") | Search modes - Boolean/Phrase | 8,494 |
| S3 | S1 OR S2 | Search modes - Boolean/Phrase | 15,499 |
| S2 | TI (TKA OR TKR) OR AB (TKA OR TKR) | Search modes - Boolean/Phrase | 5,729 |
| S1 | MH "Arthroplasty, Replacement, Knee" | Search modes - Boolean/Phrase | 14,527 |

# Cochrane Central Register of Controlled Trials (Cochrane Library)

ID Search Hits

#1 MeSH descriptor: [Arthroplasty, Replacement, Knee] this term only 2327

#2 MeSH descriptor: [Knee Prosthesis] this term only 680

#3 (TKA OR TKR):ti,ab,kw 2926

#4 #1 OR #2 OR #3 4359

#5 MeSH descriptor: [Knee] this term only 754

#6 MeSH descriptor: [Knee Joint] this term only 3011

#7 MeSH descriptor: [Osteoarthritis, Knee] this term only 3631

#8 knee*:ti,ab,kw 26320

#9 #5 OR #6 OR #7 OR #8 26320

#10 MeSH descriptor: [Arthroplasty, Replacement] this term only 151

#11 (arthroplast* or replace*):ti,ab,kw 39106

#12 #10 OR #11 39106

#13 #9 AND #12 7670

#14 #4 OR #13 7876

#15 MeSH descriptor: [Preoperative Care] this term only 4062

#16 MeSH descriptor: [Preoperative Period] this term only 254

#17 (preop* or presurg* or *prehab* or teleprehab* or prepar*):ti,ab,kw 80705

#18 #15 or #16 or #17 80705

#19 MeSH descriptor: [Patient Education as Topic] this term only 8337

#20 MeSH descriptor: [Health Education] this term only 3700

#21 MeSH descriptor: [Consumer Health Information] explode all trees 445

#22 MeSH descriptor: [Teach-Back Communication] this term only 7

#23 MeSH descriptor: [Educational Technology] explode all trees 3641

#24 MeSH descriptor: [Patient Education Handout] this term only 0

#25 ((health* or educat* or inform* or knowledge or teach*) NEAR/2 (class* or group* or program* or school* or booklet* or leaflet* or DVD* or YouTube or video* or website* or "web platform" or "web platforms" or "web page" or "web pages" or web-page* or microsite* or app or application* or multimedia)):ti,ab,kw 34544

#26 ((patient* NEAR/1 educat*) or psychoeducat*):ti,ab,kw 16450

#27 MeSH descriptor: [Exercise] explode all trees 22364

#28 MeSH descriptor: [Exercise Therapy] explode all trees 12291

#29 MeSH descriptor: [Exercise Movement Techniques] explode all trees 1903

#30 MeSH descriptor: [Rehabilitation] this term only 305

#31 MeSH descriptor: [Hospitals, Rehabilitation] this term only 1

#32 MeSH descriptor: [Rehabilitation Centers] this term only 305

#33 MeSH descriptor: [Rehabilitation Nursing] this term only 54

#34 MeSH descriptor: [Rehabilitation Research] this term only 3

#35 MeSH descriptor: [Recreation Therapy] this term only 18

#36 MeSH descriptor: [Telerehabilitation] this term only 82

#37 MeSH descriptor: [Physical and Rehabilitation Medicine] this term only 18

#38 MeSH descriptor: [Physical Therapy Modalities] this term only 3438

#39 MeSH descriptor: [Occupational Therapy] this term only 708

#40 MeSH descriptor: [Hydrotherapy] this term only 186

#41 ((joint* or knee* or motor or physical* or cardio* or strength* or propriocept* or balance or neuromuscular or aerobic or weight or stretch* or resistance or endurance or aqua*) NEAR/1 (school* or train* or activit* or fit* or program* or class* or therap*)):ti,ab,kw 59864

#42 (*rehab* or *prehab* or telerehab* or teleprehab* or *exercis* or *physiotherap* or hydrotherap* or "occupational therapy" or swim* or cycl* or bik* or "self management" or self-management):ti,ab,kw 201739

#43 MeSH descriptor: [Psychotherapy] explode all trees 22131

#44 MeSH descriptor: [Mind-Body Therapies] explode all trees 5852

#45 MeSH descriptor: [Counseling] this term only 3897

#46 MeSH descriptor: [Distance Counseling] this term only 10

#47 MeSH descriptor: [Directive Counseling] explode all trees 1086

#48 (psychotherap* or "guided imagery" or CBT or relax* or hypnosis or "motivational interviewing" or mindfulness or counsel* or "pain coping skills training"):ti,ab,kw 56223

#49 ((psychologic* or behavio* or cognitive or emotion* or mind) NEAR/1 (intervention* or technique* or therap* or treat* or prepar* or restructur* or reframe* or distract*)):ti,ab,kw 34075

#50 MeSH descriptor: [Health Promotion] explode all trees 5902

#51 MeSH descriptor: [Diet Therapy] explode all trees 5514

#52 MeSH descriptor: [Life Style] explode all trees 4886

#53 MeSH descriptor: [Alcohol Abstinence] this term only 62

#54 MeSH descriptor: [Smoking Cessation] this term only 3779

#55 MeSH descriptor: [Smoking Reduction] this term only 9

#56 MeSH descriptor: [Tobacco Use Cessation] this term only 94

#57 ("physical activity" or "weight loss" or "weight reduction" or diet*):ti,ab,kw 108647

#58 ((behavio* or lifestyle* or health*) NEAR/1 (chang* or modif* or motivat* or promot* or educat* or inform* or teach*)):ti,ab,kw 32720

#59 ((smoking or tobacco or alcohol) NEAR/1 (cessat* or reduc* or stop* or quit*)):ti,ab,kw 11986

#60 MeSH descriptor: [Nutrition Therapy] this term only 122

#61 MeSH descriptor: [Dietary Supplements] explode all trees 11356

#62 MeSH descriptor: [Functional Food] this term only 105

#63 MeSH descriptor: [Micronutrients] explode all trees 3348

#64 MeSH descriptor: [Minerals] explode all trees 3650

#65 ((nutrition* NEAR/1 supplement*) or probiotic* or prebiotic* or synbiotic* or "functional food" or nutraceutical* or nutrient* or glucosamine or chondroitin or curcumin or "fish oil" or "fish oils" or "omega 3" or vitamin* or mineral* or "trace element" or "trace elements" or flavonoid* or (hydroly* NEAR/1 collagen)):ti,ab,kw 61835

#66 MeSH descriptor: [Transcutaneous Electric Nerve Stimulation] this term only 1035

#67 (electrotherap* or "transcutaneous electrical nerve stimulation" or TENS):ti,ab,kw 2901

#68 MeSH descriptor: [Therapy, Soft Tissue] explode all trees 1365

#69 MeSH descriptor: [Trigger Points] this term only 101

#70 (massag* or "soft tissue therapy" or "trigger point" or "trigger points"):ti,ab,kw 5674

#71 MeSH descriptor: [Orthotic Devices] explode all trees 1307

#72 (orthotic* or orthos* or insole* or "arch support" or (knee NEAR/1 brace*)):ti,ab,kw 5345

#73 MeSH descriptor: [Acupuncture] this term only 141

#74 MeSH descriptor: [Acupuncture Therapy] explode all trees 4361

#75 (acupuncture or acupressure or "dry needling"):ti,ab,kw 14658

#76 MeSH descriptor: [Rehabilitation, Vocational] explode all trees 428

#77 ((occupation* or vocation*) NEAR/1 rehab*):ti,ab,kw 733

#78 #19 OR #20 OR #21 OR #22 OR #23 OR #24 OR #25 OR #26 OR #27 OR #28 OR #29 OR #30 OR #31 OR #32 OR #33 OR #34 OR #35 OR #36 OR #37 OR #38 OR #39 OR #40 OR #41 OR #42 OR #43 OR #44 OR #45 OR #46 OR #47 OR #48 OR #49 OR #50 OR #51 OR #52 OR #53 OR #54 OR #55 OR #56 OR #57 OR #58 OR #59 OR #60 OR #61 OR #62 OR #63 OR #64 OR #65 OR #66 OR #67 OR #68 OR #69 OR #70 OR #71 OR #72 OR #73 OR #74 OR #75 OR #76 OR #77 448388

#79 #14 and #18 and #78 651

#80 MeSH descriptor: [Animals] explode all trees 15483

#81 MeSH descriptor: [Humans] explode all trees 8286

#82 #80 NOT #81 7197

#83 #79 NOT #82 651

#84 #79 NOT #82 with Publication Year from 2009 to 2019, in Trials 530
