# Supplementary material for: Content and delivery of pre-operative interventions for patients undergoing total knee replacement: a rapid review
Source: Syst Rev. 2022 Sep 2;11:184. doi: 10.1186/s13643-022-02019-x (PMC9436722; doi:10.1186/s13643-022-02019-x)
Supplement: Supplementary file 3 — Additional file 3. Data items. Data items extracted for outcomes studies (Supplementary Table 2) and views studies (Supplementary Table 3). [file 13643_2022_2019_MOESM3_ESM.docx]

**Content and delivery of pre-operative interventions for patients**

**undergoing total knee replacement: a rapid review**

**Additional File 3: Data items**

**Supplementary Table 2: Outcomes study data items**

| **Data area** | **Data item** | **Definition** |
| --- | --- | --- |
| General study information | Number | Study number (allocated by reviewer) |
|  | Title | Study title |
|  | First author | First author of study |
|  | Year | Year of publication |
|  | Country | Country of origin |
| Study characteristics | Aim | Study aim and/or objectives |
|  | Design | Study design |
|  | Methods overview | Brief summary of study methods, including details of any comparator group(s) and co-interventions |
|  | Sample size calculation | Sample size calculation if reported |
|  | Outcomes | All outcomes reported for patients undergoing TKR that are directly relevant to patients and the assessment time points (this includes patient-reported outcomes, objectively measured patient outcomes, patient healthcare utilisation and patient harms, but does **not** include costs, healthcare workload or blood test results) |
| Participant characteristics | Eligibility criteria | Eligibility criteria e.g. type of surgery, time pre- or post-surgery, age threshold etc. |
|  | Total number randomized | Total number of participants randomized |
|  | Completion number | Number of participants who completed outcomes at the final assessment time point |
|  | Number per subgroup | Number of participants in specific subgroups if applicable e.g. THR versus TKR etc. |
|  | Number per intervention/ control group | Number of participants in the intervention and control groups |
|  | Age | Mean ± SD for all participants undergoing TKR if provided, otherwise any details about participant age |
|  | Gender/sex | % female for all participants undergoing TKR if provided, otherwise any details about participant gender/sex |
|  | BMI | Mean ± SD for all participants undergoing TKR if provided, otherwise any details about participant BMI |
| Intervention overview | Type | Type of intervention based on the following categories: education, exercise, psychological, lifestyle, other. A single intervention may be classified as more than one type if appropriate |
|  | Summary | Brief overview of the intervention e.g. high-intensity strength training programme, educational website, CBT-based intervention etc. |
|  | Rationale^a^ | Details about the rationale, theory or goals of the intervention. These may refer to the intervention overall or to specific intervention components e.g. the aim of the educational component of the intervention was to augment patients’ knowledge and engagement in exercises, the intervention was based on the principles of self-efficacy etc. |
| Intervention components | Component(s)^a^ | Any types of exercise, educational topics or other activities included in the intervention or used to support engagement with the intervention e.g. strengthening exercises, information on pain management, goal setting etc. (for informational materials such as a booklet, only the information topics should be listed here, with the type of delivery format being listed in the 'delivery mode(s)' column) |
| Intervention delivery approaches | Provider(s)^a^ | The person/people who provided the intervention, including their disciplinary background e.g. nurses, specially trained physiotherapists, expert patients etc. |
|  | Delivery mode(s)^a^ | How the intervention was delivered, including whether it was provided to individuals or a group e.g. face-to-face supervised sessions in groups of three, booklet etc. |
|  | Setting(s)^a^ | The types of location where the intervention was delivered e.g. patient’s home, hospital, outpatient clinic etc. |
|  | Schedule^a^ | When the intervention was delivered, the number of times the intervention was delivered and/or the time period over which the intervention was delivered e.g. 3 sessions per week delivered for 8 weeks prior to surgery, up to 10 sessions delivered between being listed for surgery and undergoing surgery etc. |
|  | Intensity^a^ | The duration, intensity and dose of individual sessions of an intervention e.g. 20 minute educational sessions, 60 minute exercise sessions with a training intensity starting at 14 repetitions maximum and progressing to 10 repetitions maximum. Progression is only included if the authors state that the exercises were progressed/increased (or equivalent) |
|  | Tailoring^a^ | Whether the intervention was personalised according to participants’ individual needs e.g. lifting weights was based on repetitions maximum, the cognitive behavioural therapy intervention was tailored to each patient etc. Providing an opportunity to ask questions alone is not sufficient to class the intervention as tailored |
| Study findings | Summary | Brief summary of the overall study findings, including reasons for dropouts and adherence to the intervention if reported. Only record TKR-specific findings for studies with multiple subgroups where appropriate. |
|  | Outcomes improved | Patient outcomes for which there were statistically significant between group differences (alpha = 0.05) in favour of the intervention group, including p-values (but not effect sizes) |
|  | Outcomes not improved | Patient outcomes for which there were **not** statistically significant between group differences (alpha = 0.05) in favour of the intervention group, including p-values (but not effect sizes) |
|  | Harms | Details of any harms associated with the intervention |

*BMI* body mass index, *THR* total hip replacement, *TKR* total knee replacement, *SD* standard deviation

^a^ The intervention data items were based on the Template for Intervention Description and Replication (TIDieR) checklist and guide (1).

**Supplementary Table 3: Views study data items**

| **Data area** | **Data item** | **Definition** |
| --- | --- | --- |
| General study information | Number | Study number (allocated by reviewer) |
|  | Title | Study title |
|  | First author | First author of study |
|  | Year | Year of publication |
|  | Country | Country of origin |
| Study characteristics | Aim | Study aim and/or objectives |
|  | Design | Study design |
|  | Methods overview | Brief summary of study methods, including the phenomenon of interest, context, data collection methods, data analysis methods and any theories used to interpret the results as appropriate |
| Participant characteristics | Eligibility criteria | Eligibility criteria e.g. type of surgery, time pre- or post-surgery, BMI threshold etc. |
|  | Total number enrolled | Total number of participants in the study |
|  | Completion number | Number of participants who completed the study |
|  | Number per subgroup | Number of participants in specific subgroups if applicable e.g. THR versus TKR etc. |
|  | Age | Mean ± SD for all participants who met the review eligibility criteria, otherwise any details about participant age |
|  | Gender/sex | % female for all participants who met the review eligibility criteria, otherwise any details about participant gender/sex |
|  | BMI | Mean ± SD for all participants who met the review eligibility criteria, otherwise any details about participant BMI |
| Study findings | Summary | Brief summary of the study findings. Only record TKR-specific findings for studies with multiple subgroups where appropriate. |
|  | Intervention type(s)^a^ | Any intervention types for which intervention component(s) or delivery approach(es) are described |
|  | Component(s)^a^ | Details of participants' experiences and perspectives of specific intervention components, linked to a specific type of intervention where possible |
|  | Provider(s)^a^ | Details of participants' experiences and perspectives of specific intervention provider(s), linked to a specific type of intervention where possible |
|  | Delivery mode(s)^a^ | Details of participants' experiences and perspectives of specific intervention delivery modes, linked to a specific type of intervention where possible |
|  | Setting(s)^a^ | Details of participants' experiences and perspectives of specific intervention settings, linked to a specific type of intervention where possible |
|  | Schedule^a^ | Details of participants' experiences and perspectives of specific intervention schedules, linked to a specific type of intervention where possible |
|  | Intensity^a^ | Details of participants' experiences and perspectives of specific intervention intensities, linked to a specific type of intervention where possible |
|  | Tailoring^a^ | Details of participants' experiences and perspectives of intervention tailoring, linked to a specific type of intervention where possible |

*BMI* body mass index, *TKR* total knee replacement, *THR* total hip replacement, *SD* standard deviation

^a^ The study findings data items were based on the Template for Intervention Description and Replication (TIDieR) checklist and guide (1)

**Reference**

1. Hoffmann TC, Glasziou PP, Boutron I, Milne R, Perera R, Moher D, et al. Better reporting of interventions: template for intervention description and replication (TIDieR) checklist and guide. BMJ. 2014;348:g1687.
