# Supplementary material for: Content and delivery of pre-operative interventions for patients undergoing total knee replacement: a rapid review
Source: Syst Rev. 2022 Sep 2;11:184. doi: 10.1186/s13643-022-02019-x (PMC9436722; doi:10.1186/s13643-022-02019-x)
Supplement: Supplementary file 4 — Additional file 4. Outcomes studies’ characteristics and results. Characteristics and results of the included outcomes studies (Supplementary Table 4). [file 13643_2022_2019_MOESM4_ESM.docx]

**Content and delivery of pre-operative interventions for patients**

**undergoing total knee replacement: a rapid review**

**Additional File 4: Outcomes studies’ characteristics and results**

**Supplementary Table 4: Characteristics and results of the included outcomes studies**

| **Citation, country** | **Primary aim** | **Design** | **Participants^ab^** | **Patient outcomes^a^** | **Key findings^ac^** |
| --- | --- | --- | --- | --- | --- |
| Bergin et al., 2014 (1), USA | To investigate the effects of pre-op incentive spirometry education on post-op outcomes amongst patients undergoing TKR or THR | **Quantitative: Two-arm RCT**  Participants were recruited from a single community not-for-profit hospital.  **Control group:**  Attended a pre-op education class involving provision of an incentive spirometry device and informal education about its use.  Were asked to complete daily study diaries on incentive spirometry use for up to 1 week post-op.  **Intervention group:**  Attended the same pre-op education class as the control group.  Following the main education class, also received formal education about incentive spirometry and were instructed to use the incentive spirometry device for 1 week pre-op.  Were asked to complete daily study diaries on incentive spirometry use for 1 week pre-op and up to 1 week post-op. | **Control group:**  N=27  **Intervention group**  N=21  (N is the number of participants included in the analysis for the outcomes listed. Participants who had not achieved their baseline incentive spirometry volume by the time of discharge were excluded) | Average hours for incentive spirometry volume to return to baseline post-op  Pain at return to baseline incentive spirometry volume | Average hours for incentive spirometry volume to return to baseline was significantly greater in the intervention group compared to the control group.  Pain at return to baseline incentive spirometry volume did not differ significantly between groups. |
| Blasco et al., 2020 (2), Spain | To investigate the effects of pre-op balance training on early post-op balance and functional outcomes amongst patients undergoing TKR and assess whether hospital and domiciliary training are equally effective | **Quantitative: Three-arm RCT**  Participants were recruited from one hospital.  **Control group:**  Did not receive a pre-op intervention and were instructed to continue their usual activities.  **Hospital (H) group:**  Participated in a 12-session strength and balance/ proprioceptive training program for 4 weeks pre-op. The program included 4-5 balance and proprioceptive exercises per session. The sessions were all supervised by physiotherapists in a hospital setting.  **Domiciliary (D) group:**  Participated in a 12-session strength and balance/ proprioceptive training program for 4 weeks pre-op. The program involved similar exercise types and volume to the H group program. The program was delivered via one instruction session, followed by unsupervised sessions, a review session after two weeks and weekly telephone calls to check compliance. | **Control group:**  N=29  Age: 70.9±9.5  Sex: 58%  BMI: 31.2±4.6  **H group:**  N=28  Age: 70.2±7.2  Sex: 76%  BMI: 32.5±4.9  **D group:**  N=29  Age: 72.3±4.5  Sex: 73%  BMI: 30.8±5.7  (Demographic details are for the 26 control group, 25 H group and 26 D group participants who were included in the analysis) | BBS, KOOS, TUG, functional reach test, isometric knee extensor strength and active knee flexion and extension ROM at baseline (5-8 weeks pre-op), 1 week pre-op, 2 weeks post-op and 6 weeks post-op  One-leg standing test at baseline, 1 week pre-op and 6 weeks post-op  EQ-5D at baseline and 6 weeks post-op | No adverse events related to the pre-op interventions occurred.  The functional reach test scores differed significantly between groups at baseline.  Significant between-group differences in favor of the H and D groups compared to the control group were identified for:   - change from baseline to 1 week pre-op in all outcomes except for knee flexion ROM - change from baseline to 6 weeks post-op in the BBS   No other significant between-group differences were identified. |
| Brown et al., 2012 (3), USA | To investigate whether patients with knee OA who participate in an exercise program before TKR surgery report higher quality of life at 3 months post-op compared to patients who do not participate in a pre-op exercise program | **Quantitative: Pilot two-arm RCT** Participants were recruited from an individual doctor's office.  **Control group:**  Received usual care only (usual pre-op care not described).  **Intervention group:**  Participated in an exercise program three times weekly (one supervised session, two unsupervised sessions) for 8 weeks pre-op. The exercise program included a warm-up, eight resistance exercises, six flexibility exercises, three step exercises and a cool-down. | **Control group:**  N=15  BMI: 34.6±7.6  **Intervention group:**  N=17  BMI: 38.8±8.8 | SF-36 at 3 months post-op | Amongst the intervention group participants, the adherence rate was 89% and the average length of the pre-op exercise program was 6.3±1.5 weeks.  The SF-36 physical function scores were significantly higher in the intervention group than the control group at 3 months post-op.  No other significant between-group differences were identified. |
| Brown et al., 2014 (4), USA | To investigate whether patients with knee OA who participate in an exercise program before TKR surgery report higher self-efficacy to exercise and outcome expectations for exercise compared to patients who do not participate in a pre-op exercise program | **Quantitative: Two-arm RCT**  Participants were recruited from a single orthopaedic surgery clinic.  **Control group:**  Received usual care only (2-3 hour educational program provided ~2 weeks pre-op).  **Intervention group:**  Participated in a pre-op exercise program three times weekly (one supervised session, two unsupervised sessions) for 8 weeks pre-op. The exercise program was based on the constructs of the Social Cognitive Theory and included a warm-up, eight resistance exercises, six flexibility exercises, three step exercises and a cool-down. | **Control group:**  N=18  Age: 67±9.5  Gender/sex: 56%  BMI: 34.6±7.6  **Intervention group:**  N=19  Age: 60±8.3  Gender/sex: 45%  BMI: 38.8±8.8  (Demographic details are for the 15 control and 16 intervention group participants who were retained at the final data collection point) | Self-efficacy to exercise scale and outcome expectations for exercise scale at 8 weeks pre-op, 1 week pre-op, 1 week post-op and 2 weeks post-op | No significant between-group differences were identified for any outcome at any time point. |
| Calatayud et al., 2017 (5) Casaña et al., 2019 (6), Spain | To investigate the effects of a pre-op high intensity resistance exercise program, with secondary balance component, on outcomes and length of hospital stay amongst patients undergoing TKR | **Quantitative: Two-arm RCT**  Participants were recruited from a single hospital.  **Control group:**  Did not receive any supervised exercise training but were advised to perform two isometric knee extension strengthening exercises and one isometric hip flexion strengthening exercise every day.  **Intervention group:**  Participated in a supervised pre-op high-intensity exercise program three times weekly for 8 weeks pre-op. The exercise program included a warm-up, four lower limb strength exercises, two balance exercises and a cool-down. | **Control group:**  N=25  Age: 66.7±3.1  Gender: 86.4%  BMI: 31±3.8  **Intervention group:**  N=25  Age: 66.8±4.8  Gender: 81.8%  BMI: 32±4.2  (Demographic details are for the 22 control group and 22 intervention group included in the analysis) | WOMAC; SF-36 (physical functioning scale only); 10cm VAS pain scale; isometric knee flexion, knee extension and hip abduction strength; active knee flexion and extension ROM; TUG, stair ascent-descent test and center of pressure during the Romberg test with eyes open and eyes closed at 8 weeks pre-op, after 8 weeks of training (pre-op), 1 month post-op and 3 months post-op  Length of hospital stay | Significant between-group differences in favor of the intervention group were identified for:   - WOMAC; SF-36; VAS pain scores; isometric knee flexion and hip abduction strength; knee flexion and extension ROM; TUG; stair ascent-descent test; and the center of pressure area during both Romberg tests at all follow-up time points - most of the additional center of pressure measurements at one or more follow-up time points. - isometric knee extension strength at all follow-up time points other than 1 month post-op. - length of hospital stay |
| das Nair et al., 2018 (7), UK | To investigate the feasibility of conducting an RCT to evaluate the clinical and cost-effectiveness of a CBT-based pre-op psychological intervention for patients undergoing TKR due to knee OA | **Mixed methods: Two-arm feasibility RCT**  Participants were recruited from two NHS hospital knee surgery pathways. Only individuals with anxiety or depression (defined as a score of >7 on the anxiety or depression subscale of the HADS) were eligible to participate.  **Control group:**  Received usual care (no psychologist input or focus on the participant’s psychological state).  **Intervention group:**  Received usual care plus up to 10 sessions of a CBT-based pre-op psychological intervention. The psychological intervention was delivered by a psychologist in the participant’s home or at a hospital and included psychoeducation on mood and pain, values-based goal setting, self-management and behavioral activation, relaxation and mindful breathing, cognitive restructuring and post-op planning. | **Control group:**  N=25  Age: 66.7±9.9  Gender: 36%  **Intervention group:**  N=25  Age: 65.7±8.6  Gender: 56%  (One additional participant was randomized but did not meet the inclusion criteria so their data were excluded from the analyses) | WOMAC, Intermittent and Constant Osteoarthritis Pain Scale, Beck Depression Inventory, Beck Anxiety Inventory and EQ-5D-5L at baseline, 4 months post-randomization and 6 months post-randomization  Service use questionnaire | Six intervention group participants completed the intervention as planned.  The number of intervention sessions received was 2-8 (mode=3).  WOMAC function scores were significantly lower (indicating better function) in the intervention group than the control group at 6 months post-randomization, but the authors suggested that this was probably a chance finding due to multiple comparisons.  No other significant between group differences in outcomes were identified.  The authors concluded that a definitive RCT is feasible with changes to the intervention and study procedures. |
| Doiron-Cadrin et al., 2020 (8), Canada | To investigate the feasibility and potential effects on pain and disability of a telerehabilitation prehabilitation programme in comparison to in-person prehabilitation or usual care amongst patients listed for TKR or THR | **Quantitative: Pilot three-arm RCT**  Participants were recruited from one tertiary care hospital and one community hospital. Only individuals with access to a high-speed internet connection were eligible.  **Control group:**  Did not receive any prehabilitation but did receive the hospital's usual pre-op care (single home visit from a community physiotherapist involving provision of a booklet on the surgery, medication and rehabilitation).  **In-person prehabilitation (I) group:**  Participated in a 12-week pre-op exercise program involving two in-person supervised sessions and five unsupervised sessions per week. The program included education, walking aid adjustment, a warm-up, hip and knee strengthening exercises, hip and knee mobility exercises, proprioceptive exercises and completion of a log book.  **Telerehabilitation prehabilitation (T) group:**  Participated in a 12-week pre-op exercise program involving two sessions supervised via an internet-based telecommunication mobile application per week and five unsupervised sessions per week. If the participant’s pain increased during the program, they could have an in-person appointment. The exercise program involved the same components at the I group exercise program. | **Control group:**  N=6  **I group:**  N=5  **T group:**  N=6 | French-Canadian versions of the LEFS, WOMAC and SF-36; self-paced walk test, TUG and timed stair test at baseline and 12 weeks later  French-Canadian version of the GRC scale at the 12 week follow-up (success was defined as a GRC score of +2 or higher, failure was defined as a GRC score of +1 or lower)  Participants in the T group completed a satisfaction questionnaire but the results are not reported separately for patients listed for TKR | Of the 12 participants in the T group (six TKR, six THR), one withdrew due to shoulder pain and four requested in-person appointments due to increased pain/other musculoskeletal problems. Issues with the planned videoconferencing application meant two additional video conferencing applications and telephone calls were used to deliver some of the T group supervised sessions.  The proportions of success based on the GRC scale scores were significantly higher in the I and T groups compared to the control group.  No other significant between-group differences were identified.  The authors concluded that the telerehabilitation prehabilitation program appears safe, feasible and satisfactory for patients and a fully-powered RCT of the program is warranted. |
| Domínguez‑Navarro et al., 2021 (9), Spain | To investigate the effects of pre-op combined strength and balance training on balance and functional outcomes amongst patients undergoing TKR, and compare the combined training to strength training only and no intervention | **Quantitative: Three-arm RCT**  Participants were recruited from one hospital.  **Control group:**  Did not receive any pre-op experimental interventions.  **Strengthening (S) group:**  Participated in a 12-session strength-training program for 4 weeks pre-op. The program included a warm-up, six lower limb strengthening exercises and a cool-down.  **Strengthening plus balance and proprioceptive exercise (B) group:**  Participated in a 12-session strength and balance/ proprioceptive training program for 4 weeks pre-op. The program included all elements of the ST group program plus approximately 4-5 balance and proprioceptive exercises per session. | **Control group:**  N=28  Age: 70.2±5.6  Gender/sex: 66.7%  **S group:**  N=28  Age: 70.8±5.4  Gender/sex: 58.3%  **B group:**  N=26  Age: 70.4±6.4  Gender/sex: 65.0%  (Demographic details are for the 21 control, 24 S and 20 B participants who completed the 6-week post-op assessments) | BBS, KOOS, TUG, functional reach test, isometric knee extensor strength and active knee flexion and extension ROM at baseline (5-8 weeks pre-op), 1 week pre-op (end of pre-op intervention), 2 weeks post-op, 6 weeks post-op and 1 year post-op  Single leg stance and EQ-5D at baseline, 1 week pre-op, 6 weeks post-op and 1 year post-op | No adverse events directly related to the pre-op interventions occurred.  Overall adherence to the pre-op interventions was 11.2 sessions (SD 0.7, adherence for each group not reported).  Significant between-group differences in favor of the S and B groups compared to the control group were identified for:   - change from baseline to 1 week pre-op in BBS, KOOS-ADL, KOOS-symptoms, KOOS-pain, knee extensor strength and TUG - change from baseline to 6 weeks pre-op in knee extensor muscle strength - single-leg stance test at 1 year post-op   No other significant between-group differences were identified. |
| Eschalier et al., 2017 (10), France | To investigate the effects of an information booklet on TKR-focused knowledge amongst patients undergoing TKR | **Quantitative: Two-arm RCT**  Participants were recruited from a single teaching hospital.  **Control group:**  Received standard oral pre-op information from their surgeon.  **Intervention group:**  Received standard oral pre-op information from their surgeon.  At the end of their pre-anesthesia appointment, also received an information booklet and were asked to read it multiple times. The booklet included 10 chapters covering numerous topics related to TKR. The booklet was developed and validated through a previous research study^[[1]](#footnote-1)^ | **Control group:**  N=20  Age: 66.8±5.8  Sex: 50%  BMI: 31.6±5.4  **Intervention group:**  N=22  Age: 68.1±4.7  Sex: 45%  BMI: 31.2±5.1 | TKR knowledge questionnaire and patient beliefs questionnaire assessed at baseline (4-6 weeks pre-op), day 1 pre-op and 3-6 weeks post-op  Satisfaction with the information received at 3-6 weeks post-op  Surgery-ward length of stay  Proportion of patients discharged home | Mean total knowledge score did not differ significantly between groups at any time point.  Improvements in the knowledge score from baseline to day 1 pre-op and from baseline to 3-6 weeks post-op were significantly greater in the intervention group than the control group.  From baseline to day 1 pre-op, the proportion of expected responses for the beliefs questionnaire significantly increased for two items and significantly decreased for one item in the intervention group, and these changes were significantly greater than in the control group.  No significant between-group differences were identified for any other outcomes. |
| Gränicher et al., 2020 (11), Switzerland | To investigate the effects of pre-op physiotherapy on post-op functional, subjective and socio-economic outcomes amongst patients undergoing TKR | **Quantitative: Pilot two-arm RCT**  Participants were recruited from one University hospital.  **Control group:**  Were asked to maintain the same activity level as before their baseline assessment and not start any new therapy/training in the pre-op phase.  **Intervention group:**  Participated in 5-9 sessions of individualised physiotherapy within 3-4 weeks pre-op. The physiotherapy sessions included endurance training, education (including on self-training at home), hamstring and quadriceps PNF contract-relax-antagonist-contract techniques and individually indicated interventions (lower limb strengthening, sensorimotor training and/or electromyostimulation training) | **Control group:**  N=10  Age: 68.1±7.7  Gender/sex: 50%  **Intervention group:**  N=10  Age: 66.6±7.5  Gender/sex: 30% | Stair climbing test, active knee flexion ROM and German versions of the Lysholm Scale and Tegner Activity Scale at baseline (3-4 weeks pre-op), immediately pre-op and 3 months post-op  PGIC at 3 months post-op (clinically significant improvement was defined as a score of 1 or 2)  Length of stay at inpatient rehabilitation  Daily pre-op NRS pain scores  Pre-op MET based on participant-recorded daily activities (excluding intervention-related activities)  Participant-reported duration of post-op medication consumption | No adverse events occurred.  80% of intervention group participants completed nine physiotherapy sessions as planned.  Significant group by time interactions were identified for the Lysholm Scale pain score and Tegner Activity Scale score, with higher scores in the intervention group compared to the control group at both follow-up time points. However, the significance of between-group differences at the follow-up time points is not reported.  Pre-op METs were significantly higher in the intervention group compared to the control group.  No other significant between-group differences were identified. |
| Gstoettner et al., 2011 (12), Austria | To investigate the effects of a pre-op proprioceptive exercise program on post-op balance and function amongst patients undergoing TKR | **Quantitative: Two-arm RCT**  Participants were recruited from an orthopaedic department.  **Control group:**  No details reported.  **Intervention group:**  Participated in a proprioceptive exercise program daily (one supervised session, six unsupervised sessions) for 6 weeks pre-op. The exercise program included a warm-up, five lower limb stretches and four proprioceptive/balance exercises. | **Control group:**  N=20  Age: 66.9  Gender/sex: 70%  BMI: 28.2  **Intervention group:**  N=18  Age: 72.8  Gender/sex: 88.9%  BMI: 27.4  (SD not reported) | Standing balance test (antero-posterior stability index, medio-lateral stability index and overall stability index), timed 60m walk, stair ascent and descent test, WOMAC and KSS at 6 weeks pre-op, after the exercise training program (pre-op, intervention group only) and 6 weeks post-op | At baseline, the WOMAC function scores were significantly lower (indicating better function) in the intervention group compared to the control group.  At 6 weeks post-op, the antero-posterior stability index was significantly lower (indicating greater stability) in the intervention group compared to the control group.  No other significant between-group differences were identified. |
| Huber et al., 2015a (13), Switzerland | To investigate the effects of a pre-op neuromuscular exercise program combined with a pre-op education program compared to the pre-op education program alone on pain and function amongst patients undergoing TKR due to knee OA | **Quantitative: Two-arm RCT**  Participants were recruited by orthopaedic surgeons from two hospitals.  **Control group:**  Attended a pre-op knee school that started ~4 weeks pre-op and consisted of three individual or group sessions (one per week) delivered by a specially-trained physiotherapist. The knee school focused on educating patients about knee OA, the preparation phase for TKR and the acute rehabilitation phase following TKR.  **Intervention group:**  Attended the same pre-op knee school as the control group participants and participated in a supervised group-based neuromuscular exercise program twice weekly for 4-12 weeks pre-op. The exercise program included a warm-up, circuit program with four exercise circles (core stability/postural function, postural orientation/functional alignment, lower limb muscle strength and functional exercises) and a cool-down. | **Control group:**  N=23  Age: 71.9±8.1  Gender: 43.5%  BMI: 29.9±5.5  **Intervention group:**  N=22  Age: 68.8±8.0  Gender: 50%  BMI: 30.8±4.9 | CST, KOOS, isometric knee flexor and extensor strength bilaterally, maximal number of knee-bending in 30 sec, knee flexion and extension ROM bilaterally, timed 20m walk test, TUG, physical activity using the SenseWear armband, adapted NHANES III METs, SF-36 and EQ-5D at baseline, 1 week pre-op, 6 weeks post-op (self-report questionnaires only), 3 months post-op and 12 months post-op (self-report questionnaires only) | 63.6% of intervention group participants reported increased pain 24 hours after exercising. One intervention group participant missed two exercise sessions due to increased pain.  76.2% of the intervention group participants met the pre-defined adherence goal of attending ≥8 training sessions.  No significant between group differences were identified for any outcomes at any time point. |
| Jahic et al., 2018 (14), Bosnia and Herzegovina | To investigate the effects of a pre-op exercise program on post-op outcomes amongst patients undergoing TKR compared to a control group | **Quantitative: Two-arm RCT**  Recruitment location not explicitly stated but all participants underwent TKR surgery at the same clinic.  **Control group:**  Received detailed information about surgery and were advised not to gain weight pre- and post-op and to take care with their diet.  **Intervention group:**  Received the same information and advice as the control group and participated in a pre-op unsupervised exercise program three times daily for 6 weeks pre-op. The exercise program involved quadriceps strengthening, flexibility exercises and resistance training. | **Control group:**  N=10  Gender/sex: 70%  BMI: 27.2±1.7  **Intervention group:**  N=10  Gender/sex: 70%  BMI: 27.1±2.1 | KSS and BMI at 6 weeks pre-op, immediately pre-op, ‘post-op’ (specific time point post-op not stated), 3 months post-op, 6 months post-op and 12 months post-op | Significant between group differences in favor of the intervention group were identified for:   - KSS function score immediately pre-op - KSS knee score immediately pre-op, ‘post-op’, 3 months post-op and 6 months post-op.   No other significant between group differences were identified. |
| Leal-Blanquet et al., 2013 (15), Spain | To investigate whether an educational DVD influences pre-op expectations about post-op recovery amongst patients undergoing TKR (secondary aim was to find a biophysical profile of patients for whom the DVD could be most effective) | **Quantitative:** **Two-arm RCT**  Participants were recruited from a single center.  **Control group:**  Received verbal information about TKR during their initial appointment with the orthopaedic surgeon; a specialist nurse appointment immediately after their initial surgeon appointment; and a second nurse appointment 4 weeks later.  **Intervention group:**  Received the same verbal information about TKR as the control group.  Also watched an educational DVD during their second nurse appointment. The DVD covered the hospital stay, outpatient care, pain, function and rehabilitation. Participants watched the DVD twice (once with no interruptions, then again section by section with the opportunity to ask questions). | **Control group:**  N=50  Age: 73.4±6.5  Sex: 78%  BMI: 31.7±6.1  **Intervention group:**  N=42  Age: 72.1±7.4  Sex: 74%  BMI: 30.5±4.7 | KRES at the first nurse appointment (pre-intervention) and second nurse appointment (post-intervention) | For the KRES total score, the mean pre- and post-intervention expectations and the change in mean expectations from pre- to post-intervention did not differ significantly between groups.  For the individual KRES items, the only significant between-group differences identified were:   - lower post-intervention expectations for going up the stairs in the intervention group - greater change in mean expectations from pre- to post-intervention for going up the stairs and going down the stairs in the intervention group - greater change in mean expectations from pre- to post-intervention for knee ROM in the control group   A biophysical profile of patients for whom the DVD could be most effective was not identified. |
| Lin et al., 2019 (16), China | To investigate whether pre-op CFNB education improves post-op analgesic efficacy amongst patients undergoing TKR (and whether the education reduces nurse PCA-related workload) | **Quantitative: Two-arm RCT**  Participants were recruited from a single hospital.  **Control group:**  Received PCA pump operation training before returning to the ward and bedside PCA education on the ward.  **Intervention group:**  Received the same PCA pump training and education as the control group.  Also attended a nurse-led educational session the day before their TKR. The educational session focused on an educational pamphlet focused on PCA (including falls prevention). | **Control group:**  N=30  Age: 66.6±6.5  Gender: 73%  BMI: 26.4±4.1  **Intervention group:**  N=30  Age: 66.5±8.1  Gender: 90%  BMI: 26.7±4.0 | Knee flexion ROM, 10 item questionnaire assessing participants' knowledge of CFNB and PCA and VAS pain scores at rest and during movement at days 1 and 2 post-op | No adverse events occurred.  Significant between-group differences in favor of the intervention group were identified for:   - knowledge questionnaire scores at day 1 post-op - VAS pain scores at rest and during movement at days 1 and 2 post-op.   No other significant between-group differences were identified. |
| Matassi et al., 2014 (17), Belgium | To investigate the effects of a pre-op home exercise program on pre-op ROM and post-op ROM and function amongst patients undergoing TKR | **Quantitative: Two-arm RCT**  Participants were recruited from a single hospital.  **Control group:**  Continued their usual activities pre-op.  **Intervention group:**  Participated in a pre-op home exercise program five times weekly for 6 weeks pre-op. The exercise program consisted of quadriceps stretches, hamstring stretches and four lower limb strengthening exercises. | **Control group:**  N=61  Age: 67±7.7  Gender: 42.6%  BMI: 28±3.7  **Intervention group:**  N=61  Age: 66±7.2  Gender: 54.1%  BMI: 29±4.3 | Exercise adherence  Knee extension ROM, active and passive knee flexion ROM and KSS at 6 weeks pre-op, immediately pre-op, 6 weeks post-op, 6 months post-op and 12 months post-op  Duration in days before reaching 90° knee flexion post-op  Length of hospital stay | Two participants experienced exercise-related problems (increasing knee pain that resulted in the participant stopping the exercises, ipsilateral adductor tendinitis).  Amongst the intervention group participants, adherence to the exercise program was 79.4%±23%.  Significant between-group differences in favor of the intervention group were identified for:   - duration in days before reaching 90° knee flexion post-op - length of hospital stay   No other significant between group differences were identified.  There was a significant relationship between exercise adherence and the change in passive knee flexion ROM and the KSS knee score, but no other outcomes. |
| McKay et al., 2012 (18), Canada | To investigate the effects of a *‘simple and easy-to-implement’* pre-op lower limb strengthening exercise program on quadriceps strength amongst patients undergoing TKR | **Quantitative: Pilot two-arm RCT**  Participants were recruited from a single orthopaedic surgeon's outpatient clinic.  **Control group:**  Participated in a supervised upper body strengthening program three times weekly for 6 weeks pre-op.  **Intervention group:**  Participated in a supervised lower limb strengthening exercise program three times weekly for 6 weeks pre-op. The exercise program involved four lower limb strengthening exercises.  The exercise programs of the control and intervention groups involved the same aerobic warm-up, intensity and progression. | **Control group:**  N=12  Age: 60.6±8.1  Gender: 66.7%  BMI: 33.8±7.1  **Intervention group:**  N=10  Age: 63.5±4.9  Gender: 50%  BMI: 35.0±6.1 | Isometric quadriceps strength, flat surface walking test, stair ascent-descent test, WOMAC, SF-36 and ASES at 6 weeks pre-op, immediately post-intervention (pre-op), 6 weeks post-op and 12 weeks post-op | Attendance at the training sessions 93% in the control group and 98% in the intervention group.  No significant between-group differences were identified for any outcomes at any time point.  (A significant time-by-group interaction was identified for the SF-36 mental component scores but no significant between-group differences were identified at any individual time points). |
| Medina-Garzon, 2019 (19), Colombia | To investigate the effects of a pre-op nursing intervention based on motivational interviewing on pre-op anxiety amongst patients undergoing TKR | **Quantitative: Two-arm RCT**  Participants were recruited from a single specialized clinic.  **Control group:**  Received standard care only, which included a one-to-one pre-op education session with a nurse covering *‘the surgical preparation and the procedure’.*  **Intervention group:**  Received standard care and three sessions of a pre-op motivational interviewing intervention delivered by a nurse over a 20-day period. The intervention sessions were ‘mainly based on participants establishing their own goals to slowly change their lifestyles.’ | **Control group:**  N=28  Age: 73.7±16.6  Sex: 44.4%  **Intervention group:**  N=28  Age: 76.3±16.1  Sex: 50%  (Demographic details are for the 27 control group and 28 intervention group participants who were included in the analysis) | Amsterdam Preoperative Anxiety and Information Scale at baseline and 4 weeks after completion of the intervention | The Amsterdam Preoperative Anxiety and Information Scale scores were significantly lower (indicating lower anxiety) in the intervention group compared to the control group post-intervention. |
| Rittharomya et al., 2020 (20), Thailand | To investigate the effects of a *'Preoperative Quadriceps Exercise and Diet Control Program'* on self-efficacy for quadriceps exercise and diet control behaviour, BMI, pain, quadriceps strength, mobility and HRQOL amongst older adults listed for TKR surgery | **Quantitative: Two-arm RCT**  Participants were recruited from a single University hospital.  **Control group:**  Received standard care only, which involved provision of pre-op advice via a leaflet.  **Intervention group:**  Received standard care and a 12-week *'Preoperative Quadriceps Exercise and Diet Control Program'*. The intervention was based on Bandura’s self-efficacy theory and involved quadriceps exercises and diet control. The intervention was delivered by a researcher in two group sessions in week 1, with monitoring via telephone or LINE application in weeks 2-12. | **Control group:**  N=48  Age: 52.3% aged 60-69; 47.7% aged 70-79  Gender: 88.6%  65.9% with BMI ≥25  **Intervention group:**  N=48  Age: 54.2% aged 60-69; 45.8% aged 70-79  Gender: 89.6%  BMI: 79.2% with BMI ≥25 (Demographic details are for the 44 control group and 48 intervention group participants who were included in the analysis) | Self-Efficacy Expectation Questionnaire with quadriceps exercise and diet control sections at baseline, 2 weeks, 8 weeks and 12 weeks  NRS for knee pain intensity over the past 24 hours, quadriceps strength bilaterally, knee flexion ROM, TUG and adapted Thai version of the Mini-OAKHQOL at baseline, week 8 and week 12 | Significant between-group differences in favor of the intervention group were identified for:   - Self-Efficacy Expectation Questionnaire quadriceps exercise and diet control sections at weeks 2, 8 and 12 - right and left quadriceps strength, left knee flexion ROM, NRS pain score, TUG and adapted Thai version of the Mini-OAKHQOL at weeks 8 and 12 - right knee flexion ROM at week 12   Right knee flexion ROM at week 8 and BMI at weeks 8 and 12 did not differ significantly between groups. |
| Skoffer et al., 2016, 2020 (21, 22), Denmark | To investigate the effects of 4 weeks of pre-op and 4 weeks of post-op progressive resistance training compared to 4 week of post-op progressive resistance training only on function, strength and patient-reported outcomes amongst patients undergoing TKR | **Quantitative: Two-arm RCT**  Participants were recruited from the orthopaedic departments of one University hospital and one regional hospital.  **Control group:**  Were instructed to live as usual in the 4 weeks pre-op. Participated in a supervised progressive resistance training program three times weekly for 4 weeks post-op.  **Intervention group:**  Participated in a supervised progressive resistance training program for three times weekly for 4 weeks pre-op and 4 weeks post-op.  The pre-op and post-op training programs were the same and involved a warm-up, six lower limb resistance exercises and three lower limb stretches. | **Control group:**  N=29  Age: 70.1±6.4  Gender/sex: 58.6%  BMI: 31.8 (range 24.3-42.2)  **Intervention group:**  N=30  Age: 70.7±7.3  Gender/sex: 63.3%  BMI: 30.0 (range 22.6-42.5) | Dropout rate  Adverse events  CST, TUG, 10m walk test, 6 min walk test, isometric knee flexion and extension strength bilaterally, isokinetic affected knee flexion and extension strength, active and passive affected knee flexion and extension ROM, affected knee joint circumference, KOOS, 11 point NRS for knee pain (current pain, worst pain in past 14 days and average pain during past 14 days), prescribed and non-prescribed medication and HRQOL rating scale from 0 – 100 at 6 weeks pre-op, 1 week pre-op, 1 week post-op (strength testing of the affected leg not reported for this time point), 6 weeks post-op, 12 weeks post-op and 12 months post-op (isokinetic strength and medication not reported at this time point)  Limb symmetry indices for normalized knee extension and knee flexion strength were calculated at 12 months post-op | No participants missed exercise sessions or withdrew from the study due to intervention-related adverse events.  Amongst the intervention group participants, adherence to the pre-op training program was 94.0±8.4%.  Significant between group differences in favor of the intervention group were identified for the change from baseline in:   - affected knee flexion and extension isometric muscle strength at 1 week pre-op, 6 weeks post-op, 12 weeks post-op and 12 months post-op - CST at 1 week pre-op, 6 weeks post-op and 12 weeks post-op - TUG at 1 week pre-op, 1 week post-op and 6 weeks post-op (p = 0.050 at 12 weeks post-op) - affected knee flexion and extension isokinetic muscle strength at 6 weeks post-op and 12 weeks post-op - isometric knee extension strength of the non-affected leg and KOOS sports/recreation scores at 6 weeks post-op - NRS pains scores at 1 week pre-op   The change from baseline in knee circumference was significantly higher (indicating greater effusion) amongst participants in the intervention group compared to the control group at 1 week pre-op but no other time points.  No other significant between group differences were identified. |
| Soeters et al., 2018 (23), USA | To investigate whether a pre-op physical therapy session and access to a web-based microsite influences readiness to discharge from physical therapy, length of stay and WOMAC at 4-6 weeks post-op amongst patients undergoing TKR or THR | **Quantitative: Two-arm RCT**  Participants were recruited from a single *‘specialized orthopaedic institution’*.  **Control group:**  Received standard pre-op education (group pre-op education class and information booklet)  **Intervention group:**  Received the same standard pre-op education as the control group.  Also received a single one-to-one pre-op physical therapy session and access to a web-based microsite. The physical therapy session covered multiple TKR-related topics and provided an opportunity to practice tasks, ask questions and set goals. The microsite was focused on rehabilitation and reinforced the information provided at the physical therapy session. | **Control group:**  N=31  **Intervention group:**  N=32 | Number of post-op physical therapy visits required and length of time taken to meet inpatient physical therapy discharge criteria  Length of hospital stay  (WOMAC scores not reported separately for participants listed for TKR) | 96% of all the intervention group participants (including 31 participants undergoing THR) reported using the microsite pre-op.  Significant between-group differences in favor of the intervention group were identified for:   - number of post-op physical therapy visits required to meet inpatient physical therapy discharge criteria - length of time taken to meet inpatient physical therapy discharge criteria   Length of hospital stay did not differ significantly between groups. |
| Soni et al., 2012 (24), UK | To investigate the effects of a pre-op combined exercise and acupuncture intervention on pre- and post-op pain and function amongst patients undergoing TKR | **Quantitative: Two-arm RCT**  Recruitment location not explicitly stated but all authors were from a single NHS hospital.  **Control group:**  Received an exercise and advice leaflet.  **Intervention group:**  Received a pre-op combined exercise and Western medical style acupuncture intervention delivered by a physiotherapist once weekly for 4 weeks, then fortnightly for 4 weeks and then monthly until their surgery. The exercise program involved a circuit of 10 exercises. | **Control group:**  N=28  Age: 69.9±7.9  Sex: 46.4%  BMI: 31.1±4.9  **Intervention group:**  N=28  Age: 66.9±9.8  Sex: 53.6%  BMI: 31.4±4.2 | OKS, 10cm pain VAS, HADS, 50m timed walk, BMI and analgesic use at baseline (pre-intervention), 6 weeks post-intervention, 12 weeks post-intervention and 3 months post-op | No adverse events occurred.  VAS pain scores were significantly higher in the intervention group than the control group at baseline.  No other significant between-group differences were identified for any outcomes at any time point.  Seven participants decided not have surgery due to improved symptoms (six intervention group participants, one control group participant, odds ratio 7.64, 95% confidence interval 0.86 to 68.20, p=0.101). After 2 years, the six intervention group participants had still not undergone knee surgery but the control group participant had undergone bilateral TKR. |
| Stone et al., 2020 (25), USA | To assess the maximum change in extension from study enrolment to pre-op and two weeks post-op amongst patients using a dynamic knee extension device. The secondary objective was to investigate between group differences in patient-reported outcomes. | **Quantitative: Two-arm RCT**  Participants were recruited from one institution.  **Control group:**  Received standard care only, which included standardized physical therapy and home exercise programs for at least 4 weeks pre-op.  **Intervention group:**  Received standard care and wore a dynamic knee extension device (KneeMD) for up to 30 min three times daily until their surgery. The device was patient controlled, aimed to improve knee extension ROM and could be used during active and passive stretching. | **Control group:**  N=59  Age: 69.0±8.0  Gender: 61.8%  BMI: 32.7±5.9  **Intervention group:**  N=56  Age: 68.4±8.9  Gender: 65.2%  BMI: 33.1±7.4  (Demographic details are for the 55 control group and 46 intervention group participants who were included in the analysis) | Knee joint extension ROM (flexion contracture), KOOS and KSS function and knee scores at enrolment, the pre-op appointment and 2 weeks post-op | No adverse events related to the device occurred.  At enrolment, the mean flexion contracture was significantly greater (worse) in the intervention group compared to the control group.  At the pre-op appointment, the mean flexion contracture was lower (better) in the intervention group compared to the control group and the between-group difference approached significance (p=0.059).  At 2 weeks post-op, there was no significant between-group difference in the percentage of participants with a flexion contracture of <5°.  The authors do not report whether there were any statistically significant between-group differences in the KOOS and KSS, but the figures indicate the intervention group and control group confidence intervals for these outcomes overlap. |
| Swank et al., 2011 (26), USA | To investigate the effects of a 4-8 week pre-op TKR exercise program on lower limb strength and function amongst patients with severe knee OA | **Quantitative: Two-arm RCT**  Participants were recruited from a single orthopaedic surgeon's office.  **Control group:**  Were advised to continue their normal activities prior to their TKR.  **Intervention group:**  Participated in an exercise program at least three times weekly (one supervised session, at least two unsupervised sessions) for 4-8 weeks pre-op. The exercise program involved a warm-up, nine lower limb resistance exercises, forwards and lateral step training and a cool-down. | **Control group:**  N=35  Age: 62.6±7.6  Gender: 62.9%  BMI: 32.9±5.7  **Intervention group:**  N=36  Age: 63.1±7.3  Gender: 66.7%  BMI: 35.9±8.5 | 6 min walk test, 30 sec sit-to-stand test, stair ascent and descent test, isokinetic knee flexion and extension strength bilaterally and VAS 1-10 pain scale after completing each of the above tests at 4-8 weeks pre-op (pre-randomization) and during the week prior to the participant's TKR | Amongst the intervention group participants, average compliance with the exercise sessions was 90%.  Significant group by time interactions in favor of the intervention group were identified for:   - sit-to-stand test - stair ascent time - peak extension torque of the affected leg   No other significant group by time interactions were identified. |
| Topp et al. 2009 (27), USA | To investigate the effects of a pre-op TKR exercise program on pre- and post-op knee pain, function and quadriceps strength amongst patients with knee OA | **Quantitative: Two-arm RCT**  Participants were recruited from a single orthopaedic surgeon's office.  **Control group:**  Were advised to continue their normal activities prior to their TKR.  **Intervention group:**  Participated in an exercise program at least three times weekly (one supervised session, at least two unsupervised sessions) for at least 4 weeks pre-op. The exercise program involved a warm-up, nine lower limb resistance exercises, forwards and lateral step training and a cool-down. | **Control group:**  N=28  Age: 63.5±6.7  Gender: 36%  BMI: 32.0±6.1  **Intervention group:**  N=26  Age: 64.1±7.1  Gender: 27%  BMI: 32.2±5.9  (Gender recorded as reported in Table 1 but the text suggests the reported percentages are for males rather than females) | 6 min walk test, 30 sec sit-to-stand test, stair ascent and descent test, isokinetic knee extension strength bilaterally, isokinetic knee extension strength asymmetry and 10cm VAS for pain in the affected knee immediately after completion of each of the functional tasks at baseline (pre- randomization, minimum of 4 weeks pre-op), 1 week pre-op, 1 month post-op and 3 months post-op | Amongst intervention group participants, the average number of exercise sessions completed was 13.04±7.5.  Significant between group differences in favor of the intervention group were identified at 1 week pre-op for:   - sit-to-stand test - VAS pain scores after the 6 min walk test and the stair ascent test   No other significant between group differences were identified.  (Significance of between group-differences calculated by the review authors due to lack of reporting by the primary study authors). |
| Tungtrongjit et al., 2012 (28), Thailand | To compare post-op pain, ROM, quadriceps strength and quality of life between patients who participate in a pre-op quadriceps exercise program and patients who receive usual care only | **Quantitative: Two-arm RCT**  Recruitment location not explicitly stated but all authors were from a single hospital.  **Control group:**  Were advised to continue their normal activities prior to their TKR.  **Intervention group:**  Participated in a home-based quadriceps strengthening exercise program three times daily for 3 weeks pre-op. Participants received weekly instructions about the exercise program via telephone. The exercise program involved seated knee extensions only. | **Control group:**  N=30  Age: 65.9±7.2  Sex: 80.0%  BMI: 25.3±3.8  **Intervention group:**  N=30  Age: 63.0±7.6  Sex: 86.7%  BMI: 24.3±2.4 | 10cm VAS knee pain scale, quadriceps strength, knee flexion, extension and total ROM and modified WOMAC score (Thai version) at baseline (3 weeks pre-op), 1 month post-op, 3 months post-op and 6 months post-op | Significant between group differences in favor of the intervention group were identified for:   - VAS pain scores, all the modified WOMAC scores and quadriceps strength at 1 and 3 months post-op - modified WOMAC pain subscale scores at 6 months post-op   No other significant between group differences were identified. |
| Villadsen et al., 2014a, 2014b (29, 30), Denmark | To investigate the effects of a pre-op neuromuscular exercise program on immediate and 3 month post-op outcomes amongst patients undergoing TKR or THR | **Quantitative: Two-arm RCT**  Participants were recruited from a single hospital.  **Control group:**  Received standard pre-op education consisting of written information, an exercise leaflet and a 3-hour information session delivered in clinic by health professionals at 1 week pre-op.  **Intervention group:**  Received standard pre-op education and attended a supervised group-based neuromuscular exercise program twice weekly for 8 weeks pre-op. The exercise program included a warm-up, circuit program with four exercise circles (core stability/postural function, postural orientation, lower limb muscle strength and functional exercises) and a cool-down.  Intervention group participants accepted an additional wait of up to 5 weeks for their TKR (the Danish Health Care System guarantees patients will receive TKR within 1 month of being listed for TKR). | **Control group:**  N=40  Age: 65.1±9.0  Sex: 60.0%  BMI: 33.4±5.8  **Intervention group:**  N=41  Age: 67.1±8.8  Sex: 61.0%  BMI: 30.8±4.9 | KOOS; EQ-5D; 20m walk; five timed repeated chair stands; maximal knee bends in 30 sec; and dynamic power of knee extension, hip extension, hip abduction and multi-joint leg extension at baseline, post-intervention (pre-op), 6 weeks post-op (self-report questionnaires only) and 3 months post-op | One participant with hip OA stopped the exercise program due to increased pain, but no adverse events occurred amongst the participants undergoing TKR.  Significant between group differences in favor of the intervention group were identified for the mean change from baseline in:   - KOOS quality of life subscale, chair stands and knee bends of the operated leg at the post-intervention time point - KOOS activities of daily living subscale, KOOS pain subscale and EQ-5D VAS at 6 weeks post-op - hip abduction power bilaterally at 3 months post-op   No other significant between-group differences were identified. |
| Walls et al., 2010 (31), Ireland | To assess compliance with a pre-op home-based NMES training program amongst patients undergoing TKR | **Quantitative: Pilot two-arm RCT**  Participants were recruited from the pre-op assessment clinic of an elective orthopaedic unit.  **Control group:**  Received individual guidance from a physiotherapist on knee ROM and quadriceps strengthening exercises (recommended exercise frequency was twice daily).  **Intervention group:**  Participated in a home-based unsupervised NMES training program for 8 weeks pre-op. NMES was applied unilaterally to the quadriceps femoris muscle of the affected limb on alternate days for 2 weeks and then five times weekly for 6 weeks. | **Control group:**  N=5  Age: 63.2±11.4  Gender/sex: 80.0%  BMI: 32.8±6.3  **Intervention group:**  N=9  Age: 64.4±8.0  Gender/sex: 66.7%  BMI: 30.7±3.0  (N and demographic details are for the participants who completed the study only) | Isometric quadriceps strength, chair rise test, 25m timed walk, stair climb test, WOMAC and SF-36 at baseline, week 8 pre-op, week 6 post-op and week 12 post-op  Length of post-op hospitalization  (Quadriceps femoris cross-sectional area was also assessed but not classed as a patient outcome in this review) | Compliance with the NMES training program was 99.4% and 90.0% according to patient and stimulator reports respectively.  Significant between group differences in favor of the intervention group were identified for:   - chair rise test at week 8 pre-op and week 12 post-op - stair climb test at week 12 post-op.   No other significant between group differences were identified. |
| Wang et al., 2020 (32), China | To investigate the effectiveness of a pre-op Otago exercise program on lowering post-op limb swelling and falls occurrence and improving knee function and modified falls efficacy scale scores amongst patients undergoing TKR | **Quantitative:** **Two-arm RCT**  Participants were recruited from one hospital.  **Control group:**  Received usual orthopaedic nursing care, including health education, perioperative functional exercise and regular follow-up telephone calls.  **Intervention group:**  Received usual orthopaedic nursing care and participated in an individualised Otago exercise program three times weekly for 4 weeks pre-op. The exercise program included a warm-up, four knee strengthening exercises and balance training. | **Control group:**  N=110  Gender: 44%  **Intervention group:**  N=110  Gender: 46%  (Gender is for the 100 control and 100 intervention group participants who were included in the analysis) | Active knee flexion ROM at pre-op (exact time point not reported) and the 1^st^, 3^rd^, 7^th^ and 14^th^ days post-op  Barthel index for ADLs, HSS knee score and WOMAC at pre-op and the 14^th^ day post-op  MFES at pre-op and 1 month post-op | Significant between-group differences in favor of the intervention group were identified for:   - active knee flexion ROM at the 7^th^ and 14^th^ days post-op - HSS knee scores and Barthel index scores at the 14^th^ day post-op - MFES scores at 1 month post-op   No other significant between-group differences were identified. |
| Wilson et al., 2016 (33), Canada | To investigate the effects of a pre-op individualised education intervention on post-op pain-related interference with usual activities, pain and nausea amongst patients undergoing TKR | **Quantitative: Two-arm RCT**  Participants were recruited from a single orthopaedic preadmission clinic.  **Control group:**  Received standard care, which included an educational session delivered by a physiotherapist, a video about TKR and post-op routines and education about PCA delivered by nurses.  **Intervention group:**  Received the same standard care as the control group.  Also received a pre-op individualised education intervention, which included a booklet, an individualised education session and a follow-up telephone call. The intervention focused primarily on pain management. | **Control group:**  N=70  Age: 66±8  Sex: 61%  **Intervention group:**  N=73  Age: 67±8  Sex: 63% | Modified Brief Pain Inventory Interference sub-scale at day 3 post-op  Short Form McGill Pain Questionnaire, Overall Nausea Index and opioid and anti-emetic administration at days 1, 2 and 3 post-op | No significant between-group differences were identified for any outcomes at any time point (however the significance of between-group differences in anti-emetic administration is not specifically stated). |
| Zhao et al., 2018 (34), China | To investigate the effectiveness of electro-acupuncture preconditioning for reducing post-op cognitive impairments and post-op cognitive dysfunction amongst elderly patients undergoing TKR, and assess the safety of electro-acupuncture amongst elderly patients | **Quantitative: Two-arm RCT**  Participants were recruited through hospital-based advertisements in the outpatient clinics and wards of the orthopaedic departments of two hospitals.  **Control group:**  Received placebo electroacupuncture once daily for five consecutive days pre-op.  **Intervention group:**  Received real electroacupuncture preconditioning once daily for five consecutive days pre-op.  The same acupoints were used in both groups. | **Control group:**  N=30  Age: 66.7±3.8  Gender: 53.3%  **Intervention group:**  N=30  Age: 65.2±4.0  Gender: 60% | MMSE at 24 hours pre-op, 24 hours post-op and 72 hours post-op.  Post-op cognitive dysfunction (defined as post-op MMSE score at least 2 points lower than pre-op MMSE score) at 24 hours post-op and 72 hours post-op  Adverse events  (Serum concentrations of inflammatory cytokines were also assessed but not classed as patient outcomes in this review) | No adverse events occurred.  The MMSE global scores were significantly lower in both groups at 24 hours and 72 hours post-op compared to baseline. The decline in MMSE global scores at 72 hours post-op was significantly greater in the control group compared to the intervention group.  No other significant between group differences in MMSE scores or post-op cognitive dysfunction were identified. |

*ADLs* activities of daily living, *ASES* Arthritis Self-Efficacy Scale, *B* Strengthening plus balance and proprioceptive exercise (group), *BBS* Berg Balance Scale, *BMI* body mass index, *CBT* cognitive behavioral therapy *CFNB* continuous femoral nerve block, *CST* Chair Stand Test, *D* domiciliary (group), *DVD* audiovisual videodisc, *EQ-5D* EuroQol 5 Dimension Health Questionnaire, *GRC* Global Rating of Change, *H* hospital (group), *HADS* Hospital Anxiety and Depression Scale, *HRQOL* health-related quality of life, *HSS* Hospital for Special Surgery, *I* in-person prehabilitation (group), *KOOS* Knee Injury and Osteoarthritis Outcome Score, *KRES* Hospital for Special Surgery Knee Replacement Expectations Survey, *KSS* Knee Society Score, *LEFS* Lower Extremity Functional Scale, *MET* Metabolic equivalent of task, *MFES* Modified falls efficacy scale, *Mini-OAKHQOL* Mini-Osteoarthritis of Knee and Hip Quality of Life, *MMSE* Mini-Mental State Examination, *NHANES* National Health and Nutrition Examination Survey, *NHS* National Health Service, *NMES* neuromuscular electrical stimulation, *NRS* numeric rating scale, *OA* osteoarthritis, *OKS* Oxford Knee Score, *PCA* patient-controlled analgesia, *PGIC* Patient Global Impression of Change questionnaire, *PNF* proprioceptive neuromuscular facilitation, *post-op* post-operative, *pre-op* pre-operative, *RCT* randomized controlled trial, *ROM* range of motion, *S* strengthening (group), *SD* standard deviation, *SF-36* Standard Form-36 Health Survey, *T* tele-rehabilitation prehabilitation (group), *THR* total hip replacement, *TKR* total knee replacement, *TUG* Timed Up and Go test, *UK* United Kingdom, *USA* United States of America, *VAS* Visual Analogue Scale, *WOMAC* Western Ontario and McMaster Universities Osteoarthritis Index.

^a^ For studies with mixed populations, details of the participants, outcomes and findings are only provided if the primary source reported them separately for participants listed for total knee replacement unless otherwise stated.

^b^ N indicates the number of participants randomized unless otherwise stated. When reported in the primary source, age and BMI are presented as mean ± standard deviation in years and kg/m² respectively and gender/sex is presented as the percentage of females. Gender/sex is specified as either gender or sex if clearly reported in the primary source.

^c^ Significance refers to a statistically significant difference between the intervention group and the control group with alpha = 0.05.

**References**

1. Bergin C, Speroni KG, Travis T, Bergin J, Sheridan MJ, Kelly K, et al. Effect of preoperative incentive spirometry patient education on patient outcomes in the knee and hip joint replacement population. J Perianesth Nurs. 2014;29(1):20-7.

2. Blasco JM, Acosta-Ballester Y, Martinez-Garrido I, Garcia-Molina P, Igual-Camacho C, Roig-Casasus S. The effects of preoperative balance training on balance and functional outcome after total knee replacement: a randomized controlled trial. Clin Rehabil. 2020;34(2):182-93.

3. Brown K, Topp R, Brosky JA, Lajoie AS. Prehabilitation and quality of life three months after total knee arthroplasty: a pilot study. Percept Mot Skills. 2012;115(3):765-74.

4. Brown K, Loprinzi PD, Brosky JA, Topp R. Prehabilitation influences exercise-related psychological constructs such as self-efficacy and outcome expectations to exercise. J Strength Cond Res. 2014;28(1):201-9.

5. Calatayud J, Casana J, Ezzatvar Y, Jakobsen MD, Sundstrup E, Andersen LL. High-intensity preoperative training improves physical and functional recovery in the early post-operative periods after total knee arthroplasty: a randomized controlled trial. Knee Surg Sports Traumatol Arthrosc. 2017;25(9):2864-72.

6. Casaña J, Calatayud J, Ezzatvar Y, Vinstrup J, Benitez J, Andersen LL. Preoperative high-intensity strength training improves postural control after TKA: randomized-controlled trial. Knee Surg Sports Traumatol Arthrosc. 2019;27(4):1057-66.

7. das Nair R, Mhizha-Murira JR, Anderson P, Carpenter H, Clarke S, Groves S, et al. Home-based pre-surgical psychological intervention for knee osteoarthritis (HAPPiKNEES): a feasibility randomized controlled trial. Clin Rehabil. 2018;32(6):777-89.

8. Doiron-Cadrin P, Kairy D, Vendittoli PA, Lowry V, Poitras S, Desmeules F. Feasibility and preliminary effects of a tele-prehabilitation program and an in-person prehablitation program compared to usual care for total hip or knee arthroplasty candidates: a pilot randomized controlled trial. Disabil Rehabil. 2020;42(7):989-98.

9. Domínguez-Navarro F, Silvestre-Muñoz A, Igual-Camacho C, Díaz-Díaz B, Torrella JV, Rodrigo J, et al. A randomized controlled trial assessing the effects of preoperative strengthening plus balance training on balance and functional outcome up to 1 year following total knee replacement. Knee Surg Sports Traumatol Arthrosc. 2021;29(3):838-48.

10. Eschalier B, Descamps S, Pereira B, Vaillant-Roussel H, Girard G, Boisgard S, et al. Randomized blinded trial of standardized written patient information before total knee arthroplasty. PLoS One. 2017;12(7):e0178358.

11. Gränicher P, Stöggl T, Fucentese SF, Adelsberger R, Swanenburg J. Preoperative exercise in patients undergoing total knee arthroplasty: a pilot randomized controlled trial. Arch Physiother. 2020;10(1):13.

12. Gstoettner M, Raschner C, Dirnberger E, Leimser H, Krismer M. Preoperative proprioceptive training in patients with total knee arthroplasty. Knee. 2011;18(4):265-70.

13. Huber EO, Roos EM, Meichtry A, de Bie RA, Bischoff-Ferrari HA. Effect of preoperative neuromuscular training (NEMEX-TJR) on functional outcome after total knee replacement: an assessor-blinded randomized controlled trial. BMC Musculoskeletal Disord. 2015;16:101.

14. Jahic D, Omerovic D, Tanovic AT, Dzankovic F, Campara MT. The Effect of Prehabilitation on Postoperative Outcome in Patients Following Primary Total Knee Arthroplasty. Med Arch. 2018;72(6):439-43.

15. Leal-Blanquet J, Alentorn-Geli E, Gines-Cespedosa A, Martinez-Diaz S, Caceres E, Puig L. Effects of an educational audiovisual videodisc on patients' pre-operative expectations with total knee arthroplasty: a prospective randomized comparative study. Knee Surg Sports Traumatol Arthrosc. 2013;21(11):2595-602.

16. Lin X, Zhou Y, Zheng H, Zhang J, Wang X, Liu K, et al. Enhanced preoperative education about continuous femoral nerve block with patient-controlled analgesia improves the analgesic effect for patients undergoing total knee arthroplasty and reduces the workload for ward nurses. BMC Anesthesiol. 2019;19(1):150.

17. Matassi F, Duerinckx J, Vandenneucker H, Bellemans J. Range of motion after total knee arthroplasty: the effect of a preoperative home exercise program. Knee Surg Sports Traumatol Arthrosc. 2014;22(3):703-9.

18. McKay C, Prapavessis H, Doherty T. The effect of a prehabilitation exercise program on quadriceps strength for patients undergoing total knee arthroplasty: a randomized controlled pilot study. PM R. 2012;4(9):647-56.

19. Medina-Garzón M. Effectiveness of a Nursing Intervention to Diminish Preoperative Anxiety in Patients Programmed for Knee Replacement Surgery: Preventive Controlled and Randomized Clinical Trial. Invest Edu Enferm. 2019;37(2).

20. Rittharomya J, Aree-ue S, Malathum P, Orathai P, Belza B, Kawinwonggowit V. The Effectiveness of Preoperative Quadriceps Exercise and Diet Control Program for Older Adults Waiting for Total Knee Arthroplasty: A Randomized Controlled Trial. PRIJNR 2020;24(4):485-501.

21. Skoffer B, Maribo T, Mechlenburg I, Hansen PM, Søballe K, Dalgas U, et al. Efficacy of Preoperative Progressive Resistance Training on Postoperative Outcomes in Patients Undergoing Total Knee Arthroplasty. Arthritis Care Res (Hoboken). 2016;68(9):1239-51.

22. Skoffer B, Maribo T, Mechlenburg I, Korsgaard CG, Søballe K, Dalgas U. Efficacy of preoperative progressive resistance training in patients undergoing total knee arthroplasty: 12-month follow-up data from a randomized controlled trial. Clin Rehabil. 2020;34(1):82-90.

23. Soeters R, White PB, Murray-Weir M, Koltsov JCB, Alexiades MM, Ranawat AS, et al. Preoperative Physical Therapy Education Reduces Time to Meet Functional Milestones After Total Joint Arthroplasty. Clin Orthop Relat Res. 2018;476(1):40-8.

24. Soni A, Joshi A, Mudge N, Wyatt M, Williamson L. Supervised exercise plus acupuncture for moderate to severe knee osteoarthritis: a small randomised controlled trial. Acupunct Med. 2012;30(3):176-81.

25. Stone A, Turcotte J, Fowler M, MacDonald J, Brassard M, King P. A dynamic knee extension device improves flexion contracture before total knee arthroplasty: a randomized controlled trial. Current Orthopaedic Practice. 2020;31(4):347–51.

26. Swank AM, Kachelman JB, Bibeau W, Quesada PM, Nyland J, Malkani A, et al. Prehabilitation before total knee arthroplasty increases strength and function in older adults with severe osteoarthritis. J Strength Cond Res. 2011;25(2):318-25.

27. Topp R, Swank AM, Quesada PM, Nyland J, Malkani A. The effect of prehabilitation exercise on strength and functioning after total knee arthroplasty. PM R. 2009;1(8):729-35.

28. Tungtrongjit Y, Weingkum P, Saunkool P. The effect of preoperative quadriceps exercise on functional outcome after total knee arthroplasty. J Med Assoc Thai. 2012;95 (Suppl 10):S58-66.

29. Villadsen A, Overgaard S, Holsgaard-Larsen A, Christensen R, Roos EM. Immediate efficacy of neuromuscular exercise in patients with severe osteoarthritis of the hip or knee: a secondary analysis from a randomized controlled trial. J Rheumatol. 2014a;41(7):1385-94.

30. Villadsen A, Overgaard S, Holsgaard-Larsen A, Christensen R, Roos EM. Postoperative effects of neuromuscular exercise prior to hip or knee arthroplasty: a randomised controlled trial. Ann Rheum Dis. 2014b;73(6):1130-7.

31. Walls RJ, McHugh G, O'Gorman DJ, Moyna NM, O'Byrne JM. Effects of preoperative neuromuscular electrical stimulation on quadriceps strength and functional recovery in total knee arthroplasty. A pilot study. BMC Musculoskeletal Disord. 2010;11:119.

32. Wang Q, Ma J, Yan M, Yan Y, Wang Y, Bian D. Effects of preoperative Otago exercise program on rehabilitation in total knee arthroplasty patients. Int J Clin Exp Med. 2020;13(8):5914-22.

33. Wilson RA, Watt-Watson J, Hodnett E, Tranmer J. A Randomized Controlled Trial of an Individualized Preoperative Education Intervention for Symptom Management After Total Knee Arthroplasty. Orthop Nursing. 2016;35(1):20-9.

34. Zhao FY, Zhang ZY, Zhao YX, Yan HX, Hong YF, Xia XJ, et al. The effect of electroacupuncture preconditioning on cognitive impairments following knee replacement among elderly: A randomized controlled trial. World J Acupunct Moxibustion. 2018;28(4):231-6.

1. Eschalier B, Descamps S, Boisgard S, Pereira B, Lefevre-Colau MM, Claus D, et al. Validation of an educational booklet targeted to patients candidate for total knee arthroplasty. Orthop Traumatol Surg Res. 2013;99(3):313-9. [↑](#footnote-ref-1)
