# Supplementary material for: Content and delivery of pre-operative interventions for patients undergoing total knee replacement: a rapid review
Source: Syst Rev. 2022 Sep 2;11:184. doi: 10.1186/s13643-022-02019-x (PMC9436722; doi:10.1186/s13643-022-02019-x)
Supplement: Supplementary file 5 — Additional file 5. Views studies’ characteristics and findings. Characteristics and findings of the included views studies (Supplementary Table 5). [file 13643_2022_2019_MOESM5_ESM.docx]

**Content and delivery of pre-operative interventions for patients**

**undergoing total knee replacement: a rapid review**

**Additional File 5: Views studies’ characteristics and findings**

**Supplementary Table 5: Characteristics and findings of the included views studies**

| **Citation, country** | **Primary aim** | **Design** | **Participants^ab^** | **Key findings^a^** |
| --- | --- | --- | --- | --- |
| Aunger et al., 2020 (1), UK | To assess the feasibility of conducting an RCT investigating a novel intervention aimed at reducing sedentary behaviour in adults aged ≥60 years old listed for hip or knee replacement | **Mixed methods: Two-arm feasibility RCT**  Participants were recruited from one NHS hospital and randomised with 2:1 allocation to a control group or an intervention group.  **Control group:**  Received usual orthopaedic care (did not involve any pre-op ‘training’). Control group participants were contacted by telephone biweekly to check the status of their ongoing care, but the calls were not pre-planned and did not include behaviour change techniques.  **Intervention group:**  Received a behaviour change intervention aimed at reducing sedentary behaviour. The intervention was based on Self-Determination Theory and included education, motivational interviewing, individualised feedback, individualised incremental goal setting, environmental modification, self-monitoring with a pedometer and social support. The intervention was delivered pre-op by a researcher in two home visits, which could be combined to a single visit, and three biweekly phone calls.  Data were collected through study statistics; participant questionnaires, objective assessments and blood tests; participants’ sedentary behaviour booklets; an interview with the primary study research nurse and intervention fidelity assessments. The participant questionnaires included a ‘feasibility questionnaire’ with closed and open-ended questions.  Quantitative data from the feasibility questionnaire were analysed descriptively. Qualitative data from the feasibility questionnaires and the sedentary behaviour booklets were analysed using thematic analysis.  Exploratory analyses of outcome data were performed. The quantitative results were not reported separately for participants undergoing knee replacement.  Five criteria for progression to an RCT were pre-specified. | **Patients ≥60 years old listed for elective knee replacement**  **Control group:**  N=5  (Patients ≥60 years old listed for elective hip replacement: N=6)  **Intervention group:**  N=13  (Patients ≥60 years old listed for elective hip replacement: N=11)  Of all the intervention group participants (knee and hip), 21 completed the feasibility questionnaire in the week prior to surgery and 16 wrote comments in the sedentary behaviour booklet. | Key findings from the feasibility questionnaires and participants’ comments in the sedentary behaviour booklets included:   - Participants reported physical and mental benefits from engaging with the intervention. - All participants felt that participating in the study had at least a ‘positive impact’ on their post-operative recovery and did not expose them to risk of physical harm. - Three participants reported finding chair rises difficult, but other participants reported enjoying them^c^. - Some participants reported exercising in the evening was difficult due to issues such as tiredness, pain and wanting to relax. - Most participants reported having at least ‘some problems’ attaining their goals but no problems achieving environmental modifications^c^. - Barriers to goal attainment included physical, social and environmental difficulties, with the most commonly reported barriers being pain and weather. - Participants felt their goals suited their individual circumstances. - Some participants reported that they enjoyed using the pedometer but some participants highlighted issues with it, including that it was difficult to open and was poor at tracking the steps of people with mobility difficulties. Some participants overcame this using alternatives e.g. a health app. - Some participants reported benefitting from the social support they gained from participating in the study, including from ‘chatting to the researcher’^c^. - Most participants felt the sedentary behaviour booklet was useful but a few felt the ‘worksheets’ section was confusing and more writing space was needed^c^.   Four of the five criteria for progressing to an RCT were met. The remaining criterion was not met due to unpredictable surgery scheduling. Exploratory analyses suggested the intervention may have benefits, including reducing sedentary time. The authors concluded that an RCT investigating the intervention is feasible with some modifications. |
| Bardgett et al., 2016 (2), UK | To explore patients' views of factors that affect return to work following TKR or THR | **Qualitative: Qualitative descriptive**  Participants were recruited from the Freeman Joint Registry (clinical audit at a hospital in the UK).  Data were collected using a cross-sectional survey administered via post. The survey explored patients’ views of potential barriers and facilitators to return to work following TKR or THR. The questionnaire included closed-ended and open-ended questions, but the focus of the study was on the open-ended questions. Free text data were analysed using thematic analysis by one researcher, with verification by a second researcher. The themes and data were also discussed during team meetings. | **Patients between 6 months and 3 years post-TKR who were aged under 60 years old at the time of their TKR**  N=50  (Patients post-THR: N=52) | The following three themes were identified:   1. Most participants experienced physical and psychological improvements post-op that enabled them to improve their performance at work. 2. Many participants reported not receiving any information about return to work in the pre- or post-op phase. The information they received, for example in a booklet, tended to be targeted at older retired individuals. Participants who had received return to work advice felt it was inconsistent and not individualised to their specific needs^c^. 3. Adaptations offered by employers e.g. phased return, workspace modifications etc. largely assisted participants’ return to work. |
| Barnes et al., 2018 (3), South Africa | To investigate the breadth of pre-op education provided to, and the educational needs of, patients undergoing TKR or THR in the private sector in Bloemfontein | **Quantitative descriptive: Cross-sectional survey**  Participants were recruited from three private hospitals using convenience sampling.  Data were collected through structured interviews using a questionnaire developed by the research team.  The questionnaire was based on the Patient Learning Needs Scale^[[1]](#footnote-1)^ and the Canadian Clinical Checklist^[[2]](#footnote-2)^. It included questions about participants' demographics; rationale for undergoing surgery; pre-op education received; pre-op educational needs; and pre-op educational delivery approaches.  Participants were given the opportunity to review their answers following the interview to confirm their accuracy. Categorical data were analysed using descriptive statistics. | **Patients 2-4 days post-primary TKR**  N=36  (Patients post-THR: N=14) | The majority of the findings describe service provision rather than participants' views or experiences of the services. However, it is reported that 19% and 22% of participants post-TKR felt that they received insufficient 'information on post-operative information' (no additional details specified) and activities of daily living respectively.  The descriptions of service provision for participants post-TKR revealed 98% of participants received pre-op education from their orthopaedic surgeon, with 25% receiving pre-op education from a physiotherapist. 89% of participants received education via a pamphlet, while 39% received verbal education. The education was mainly received months pre-op (57%) or weeks pre-op (39%). Seventy-four percent of participants did not receive any education about pre-op exercises. Additionally, only 39% of participants received pre-op information on pain relief. |
| Berg et al., 2019 (4), Sweden | To explore patients’ views and experiences of the fast-track TKR and THR pathway | **Qualitative: Qualitative descriptive**  Participants were selected as a ‘strategic sample’ from one University hospital and two district hospitals.  Data were collected using semi-structured interviews. The interviews focused on participants' experiences and views of all phases of the care pathway from the decision to undergo surgery until 3 months post-op. Data were analysed using inductive content analysis involving three team members. | **Patients 3 months post-TKR**  N=11  Age: 63.6±8.0  Sex: 27%  (Patients 3 months post-THR, N=13) | Three chronological phases of the care pathway were identified: preparation, hospital stay and recovery. The findings emphasized the importance of adopting a person-centered approach during all care phases.  In the preparation phase, most participants felt the information provided about the surgery was adequate, but the amount of information participants wanted about the surgery and its risks varied widely ^c^.  Participants felt the information provided about post-op rehabilitation and recovery was insufficient.  Receiving information about the planned length of stay in the pre-op phase helped ensure most participants were willing to be discharged at day 1 post-op^c^.  Participants also received information from other sources, such as family, friends and the Internet. In some cases, this information was inaccurate. |
| Bin Sheeha et al., 2020 (5), UK | To understand patients' experiences, outcome expectations and satisfaction following TKR | **Qualitative: Phenomenology**  Participants were purposively selected from one NHS hospital.  Data were collected during a single focus group discussion using open-ended questions. The focus group explored participants’ experiences and perspectives of their TKR surgery, outcomes and health service quality.  Data were analysed using thematic analysis by two independent researchers. | **Patients one year post-primary unilateral TKR**  N=7  Age: 76.7±4.4  Gender: 71%  BMI: 33.9±6.1 | The following four themes were identified:   1. Recovery experience 2. Experience before TKR 3. ADL changes one-year post-TKR 4. Outcome expectations following TKR   Key findings related to pre-op interventions included:   - Participants' surgeons played an important role in modifying their expectations and all participants reported having a positive experience with their surgeon. One participant specifically commented on his ‘doctor’ being ‘very realistic’. - Participants valued the pre-op education class and one participant specifically linked this to being told 'you have to work yourself as well'. - Participants agreed that pre-op and post-op exercise is important to increase muscle strength. One participant recommended exercising for months (rather than days or weeks) pre-op. - Participants' views of pre-op physiotherapy and the exercises they provided varied. Two participants recommended pre-op physiotherapy/praised their physiotherapist, whilst three did not recommend it because they did not find it helpful or felt the exercises provided by physiotherapist could be obtained online. Two participants had not received any pre-op physiotherapy.   Participants also commented on alternative treatments that they used before surgery, including: insoles, which two patients found helpful but one did not; a knee brace, which one patient found helpful; and acupuncture, which two patients found helpful. Participants’ comments about insoles and a knee brace appeared to reflect their views about these treatments in general, rather than focusing specifically on their use in the pre-op phase. It was unclear whether participants’ comments about acupuncture referred to acupuncture delivered solely in the pre-operative phase. |
| Causey-Upton and Howell 2017 (6), USA | To describe patients' experiences as they prepare for discharge home post-elective TKR | **Qualitative: Transcendental phenomenology**  Participants were purposively selected from one hospital.  Data were collected through semi-structured interviews conducted 1-2 days prior to discharge. The interviews explored participants' experiences of preparing for discharge home and factors influencing their discharge readiness.  Data were analysed thematically using Moustakas' process of horizonalization^[[3]](#footnote-3)^. Various steps were taken to help ensure trustworthiness, including participants' confirming the emerging themes and peer review of the research findings and decisions between the two authors. | **Patients who had undergone elective TKR surgery and were receiving inpatient physical and occupational therapy**  N=4  Gender: 75% | Three themes were identified:   1. Being supported for discharge home 2. Having confidence in self, family and healthcare staff 3. Persevering: overcoming obstacles   The second theme reported participants’ perspectives of the pre-op education class, including:   - Participants felt the class improved their confidence by enabling them to prepare for the future. Participants also reported that the class helped them understand what to expect regarding their recovery. - Participants identified that getting their questions addressed was a significant part of their pre-op education. - Participants reported that having family present increased their confidence because it meant their family member was also aware of the recommendations provided and post-op expectations. |
| Causey-Upton et al., 2018 (7), USA | To describe existing pre-op TKR education content, providers and delivery formats in the USA | **Quantitative descriptive: Cross-sectional survey**  Participants were recruited from seven hospitals using convenience sampling (40 hospitals were contacted initially).  Data were collected using an online survey administered via email. The survey was developed by the study team based on pre-op education literature and a review of three existing pre-op TKR education programmes. The authors described the survey as a ‘pilot survey.’ The survey included 12 closed-ended and four open-ended questions. The questions covered participants’ demographic details, current pre-op TKR education programmes, ideas about optimal pre-op TKR education programmes and perceptions of the survey itself.  Data for closed-ended items were analysed using descriptive statistics. Data for open-ended items were collated together. | **Nurses**  N=2  **Physical therapist**  N=1  **Occupational therapists**  N=4 | The majority of the findings describe pre-op education service provision, rather than participants’ views or experiences of the services. However, participants highlighted various changes to their current pre-op education program design that they felt would be beneficial, including (numbers in brackets are the percentage of participants who selected the response):   - Additional education topics: functional mobility (14.3%), instrumental ADLs (28.6%), home modifications (14.3%), caregiver training (28.6%, not included in the summary tables because the training is not delivered to patients), anatomy of the knee joint (14.3%) and expected functional outcomes (28.6%). - Additional education providers: case management (42.9%), social work (14.3%) and physician assistant and/or surgeon (14.3%). - Additional changes: involve patients from all physicians at the facility (14.3%), run separate classes for patients undergoing TKR and THR (14.3%) and provide a video of a TKR patient carrying out exercises (14.3%). |
| Causey-Upton et al., 2020a, 2020b (8, 9), USA | ***Quantitative component***  To describe current pre-op TKR education design across the USA  ***Qualitative component***  To explore providers' perceptions regarding current pre-op TKR education programmes and the efficacy of different aspects of the programmes | **Mixed methods: Explanatory sequential**  ***Quantitative component: Cross-sectional survey***  Participants were recruited from the National Association of Orthopaedic Nurses (3,955 members were sent the recruitment email).  Data were collected using an online cross-sectional survey administered via email. The survey was a refined version of the pilot survey used in Causey-Upton et al. (2018) (7). The survey included 23 closed-ended questions and one open-ended question. The questions covered participants’ demographic details, current pre-op TKR education program design and perceptions of the ideal pre-op TKR education program design.  Data for closed-ended items were analysed using descriptive statistics. Data for open-ended items were collated together.  ***Qualitative component: Qualitative descriptive informed by phenomenology***  At the end of the above survey, participants were asked to give their contact details if they were interested in participating in a telephone interview. Potential participants were divided into groups by national region and years of experience providing pre-op education. A random number generator was then used to select participants from the groups who completed the survey at varying time points.  Data were collected via semi-structured interviews. During the interviews, participants were asked to describe the pre-op TKR education program at their facility and share their views about their current and the ideal pre-op TKR program design.  Data were analysed thematically using Moustakas' process of horizonalization^[[4]](#footnote-4)^. Steps taken to help ensure trustworthiness included ‘expert peer review’ and triangulating the findings with survey data. | **Orthopaedic nurses**  ***Quantitative component***  N=469  ***Qualitative component***  N=10  Sex: 90% | ***Quantitative component***  Many of the findings describe current pre-op education program design, rather than participants’ views or experiences of the programmes. Key findings regarding participants’ perceptions of the ideal pre-op TKR program design include (numbers in brackets are the percentage of participants who selected the response):   - The most frequently selected topics participants felt it would be beneficial to add to the pre-op education program, were nutrition (20.3%), caregiver training (14.5%, not included in the summary tables because the training is not delivered to patients), edema management (12.4%), instrumental ADLs (11.3%) and expected functional outcomes (11.1%). - The most frequently selected providers participants felt it would be beneficial to add to the pre-op education team were case management (29.4%), pain management (22.4%), home healthcare (16.4%), social work (15.8%) and dietetics (15.6%). - The most frequently selected delivery methods participants felt it would be beneficial to add to the pre-op educational program were online (47.3%), video (29.2%), client demonstration or the teach back method (22.2%) and workbook (10.2%). - 47.3% of participants felt it is best to educate patients awaiting TKR together with patients with other orthopaedic diagnoses, whilst 49.7% of participants felt it is better to educate patients awaiting TKR separately. - The most frequently preferred education delivery formats were combined group and individual (53.9%), group only (38.0%) and individual only (6.4%). - The most commonly preferred education delivery timings were 2 weeks pre-op (40.9%), 4 weeks pre-op (24.1%) and 3 weeks pre-op (19.0%). - The most commonly preferred number of pre-op education sessions were one (79.3%) and two (17.5%). - The most commonly preferred length of pre-op education sessions were 1 to <1.5 hours (40.3%), 1.5 to < 2 hours (25.8%) and 30-59 min (17.9%).   **Qualitative component**  Four themes were identified:   1. Knowledge is power for patients and providers: participants perceived pre-op TKR education has multiple benefits, such as patients being better prepared for the hospital stay and losing weight pre-op. Participants reported informal training, formal training and clinical orthopaedic experience helped them prepare for providing pre-op education and highlighted the importance of the 'right individuals' providing education. 2. Education should be consistent, individualised and evidence-based: participants felt that providing consistent information to all patients, and ensuring consistency across delivery modes, providers and time points, increased patient confidence and understanding. Participants also highlighted the importance of tailoring information to each individual's needs based on various factors e.g. learning style, previous experience, language needs and comorbidities. Participants reported seeking to continually improve their programmes and ensure that they are evidence-based. 3. Inter-professional practice is important but is limited by barriers: participants felt that interdisciplinary provision of pre-op education offers multiple benefits, including improved patient outcomes, but also presents multiple challenges, such as location and timing issues. 4. The structure of pre-op education is guided by pragmatics: participants highlighted that all aspects of pre-op education are affected by pragmatic and contextual factors. For example, participants felt that providing education in a group format offers multiple benefits, such as improved efficiency, but also highlighted that patients may be more reluctant to ask personal questions in a group setting. Participants’ also highlighted numerous considerations related to the timing of pre-op education sessions, number of sessions and duration of sessions. |
| das Nair et al., 2018 (10), UK | To investigate the feasibility of conducting an RCT to evaluate the clinical and cost-effectiveness of a CBT-based pre-op psychological intervention for patients undergoing TKR due to knee OA | **Mixed methods: Two-arm feasibility RCT**  Participants were recruited from two NHS hospital knee surgery pathways and randomised with 1:1 allocation to a control group or intervention group. Only patients with anxiety or depression (defined as a score of >7 on either of the HADS subscales) were eligible.  **Control group:**  Received usual care (no psychologist input or focus on the participant’s psychological state).  **Intervention group:**  Received usual care plus up to 10 sessions of a CBT-based pre-op psychological intervention. The psychological intervention was delivered by a psychologist in the participant’s home or at a hospital and included psychoeducation on mood and pain, values-based goal setting, self-management and behavioural activation, relaxation and mindful breathing, cognitive restructuring and post-op planning.  Data were collected through self-report questionnaires. A purposively selected subsample of participants were invited to participate in semi-structured interviews. The interviews explored the acceptability, barriers, and facilitators of the intervention and the study procedures. The interview data were analysed using a framework approach. | **Patients with knee OA and anxiety or depression listed for TKR**  **Control group:**  N=25 (total)  N=12 (participants who completed interviews only)  Age: 65.7±8.6 (total)  Gender: 56% (total)  **Intervention group:**  N=25 (total)  N=11 (participants who completed interviews only)  Age: 66.7±9.9 (total)  Gender: 36% (total)  (One additional participant was randomised but did not meet the inclusion criteria so their data were excluded from the analyses) | Three main themes were identified from the qualitative data:   1. Experiences of being in the study: most participants understood the rationale for the study and randomization and the information provided. 2. Participants' views of the outcome measures: most participants felt the outcome measures were appropriate but some participants raised concerns about aspects such as the number and timing of questionnaires. 3. Treatment experiences of participants in the intervention group: participants were mostly positive about the intervention and understood the thoughts-mood-pain interaction and its link with TKR. However, some participants perceived pain as physical and did not believe in the thoughts-mood-pain interaction. Some, but not all, participants found the intervention beneficial. Reported benefits included reassurance, relaxation, calmness, positive thoughts, thinking differently and developing more realistic expectations. Participants who reported benefits of the intervention felt these were due to a range of factors, such as specific intervention techniques, personal tailoring of the intervention and the psychologists’. Participants’ views of the optimal setting and delivery mode varied, with pros/cons of hospital versus home and group versus individual sessions being noted.   The authors concluded a definitive RCT is feasible with changes to the intervention and study procedures. |
| Drew et al., 2019 (11), Judge et al., 2020 (12) | ***Part 1***  To understand organizational processes that facilitate or impede the implementation of hip and knee replacement ERAS programmes  ***Part 2***  To explore patients' experiences of hip and knee replacement ERAS programmes using the ethnographer Mol's work | **Qualitative: Ethnography**  The study consisted of two parts. It was part of a larger project investigating the impact of hospital organization, surgical factors and ERAS programmes on hip and knee replacement patient outcomes and NHS costs.  ***Part 1***  Four hospitals were selected as study sites using maximum variation sampling. Data were collected using observations/job shadowing (including informal interviews) and semi-structured interviews. The observations explored the clinical setting, activities occurring, treatment protocols and barriers/facilitators to service implementation. The data collected during the observations were used to develop a topic guide for the semi-structured interviews, which explored participants' perspectives and experiences of delivering ERAS programmes and barriers/facilitators to their implementation.  The first phase of the data analysis involved inductive thematic analysis of the interview transcripts and field notes. 20% of transcripts were double coded. An abductive approach was then used to transpose the inductively coded themes onto the 31 constructs of the CFIR, grouped into the five CFIR domains (intervention characteristics, outer setting, inner setting, characteristics of individuals and process). Interpretive accounts of the data were developed.  ***Part 2***  Participants were recruited from the four study sites.  Data were collected through semi-structured interviews. The interviews explored patients’ perspectives and experiences of having a TKR/THR across the care pathway.  Data were analysed using inductive thematic analysis. 10% of transcripts were double coded. Descriptive accounts of the data were developed. | ***Part 1***  **Physiotherapists**  N=7  **Occupational therapists**  N=3  **Nurses**  N=18  **Orthopaedic surgeons**  N=5  **Anesthetist**  N=1  **Matron**  N=1  **Therapy technician assistants**  N=2  **Theatre manager**  N=1  Sex: 73.7% (all health professionals)  ***Part 2***  **Patients who had undergone TKR at one of the study sites**  N=13  Age: 71.8±10.1  Sex: 46%  (Patients who had undergone THR at one of the study sites N=23)  (Data from Table 13 rather than the text) | ***Part 1***  Seventeen CFIR constructs were considered to influence ERAS program implementation processes. These covered all five domains of the CFIR framework. The findings highlighted that implementation of hip and knee replacement ERAS programmes is influenced by multiple factors. Key findings related to pre-op education included:   - The 'joint clinics' at one site were run by nurses, physiotherapists and occupational therapists, which was felt to encourage multidisciplinary collaboration. - Written information was considered useful for reinforcing information from patients' consultations and giving them something to refer back to. - Staff at one site felt that the patient information booklets provided pre-op are key to effective rehabilitation, but were concerned that they would not be able to provide them due to funding cuts. - Participants felt the 'group dynamic' of pre-op education classes helped create a safe environment for patients to ask questions and discuss their experiences. The face-to-face format of classes was considered useful for clarifying information. - Informal communication between staff at the pre-op education classes was seen as providing an opportunity to review outcomes data/'brainstorm' approaches for improving services.   ***Part 2***  The findings were grouped into participants' perspectives and experiences of the following areas: referral process, pre-op education, pre-op preparation, waiting for the operation, anesthesia, pain management, inconsistencies in information, early post-op mobilization, discharge, post-operative (experiences), physiotherapy exercises, pain relief, post-discharge support, family and the future. Key findings related to pre-op education included:   - Participants found information about the following topics particularly helpful: how to use crutches, post-op exercises, reorganizing the home, obtaining assistive devices and arranging social support ^c^. - Participants reported wanting additional information about their recovery/expected progress to enable them to look after themselves ^c^. - A few younger patients reported wanting additional information on joint replacements in younger people, particularly regarding recovery expectations ^c^. - Participants found the pre-op education classes helpful and valued the opportunity to talk to other patients ^c^. - Participants awaiting their first joint replacement valued hearing from patients who had previously undergone surgery^c^. - Issues with the timing of pre-op education classes were highlighted, as some patients missed the class due to receiving a quick referral, whilst others forgot information from the class due to attending it too far in advance of their surgery^c^. |
| Eschalier et al., 2013 (13), France | To validate a pre-op TKR educational booklet based on feedback from health professionals and patients | **Quantitative descriptive embedded within an intervention validation study: Survey**  Participants were recruited from public or private hospitals and private practices.  Data were collected through a postal questionnaire. The questionnaire asked participants to rate each of the 10 chapters included in a pre-op TKR education booklet on a 1-10 scale for content, didactic style and illustrations. Participants could also add comments about each chapter. The scores for each chapter were summed. Total scores of 7-10 were considered an indicator that no modifications to the chapter were required, unless specific and relevant comments about the chapter were also made.  The study also involved providing the booklet to patients and asking them to complete knowledge and beliefs questionnaires before and 2 days after receiving the booklet. However, patients’ views of the booklet were not reported. | **Rehabilitation physicians**  N=5  **Orthopaedic surgeons**  N=6  **Rheumatologists**  N=2  **Primary-care physicians**  N=4  **Anesthesiologist**  N=1  **Physical therapists**  N=3  **Occupational therapists**  N=3  **Nurse**  N=1  **Psychologist**  N=1  **Social worker**  N=1 | Participants’ scores for each of the 10 chapters were generally high for content, didactic style and illustrations. However, some criticisms and suggestions were made, including:   - The diagram of knee anatomy lacked a figure legend - The explanation of knee biomechanics was too detailed - The topic ‘Treatment options for knee osteoarthritis’ might not be appropriate because the booklet was aimed at patients who had already been listed for TKR - Information about thromboembolism prophylaxis should be included in the chapter on ‘Your surgery’ rather than ‘What can you do before your surgery?’ - Extra information on contraindications should be included in the chapter on returning to sports activities |
| Eschalier et al., 2017 (14), France | To investigate the effects of an information booklet on TKR-focused knowledge amongst patients undergoing TKR | **Quantitative: Two-arm RCT**  Participants were recruited from one teaching Hospital.  **Control group:**  Received standard oral pre-op information from their surgeon.  **Intervention group:**  Received standard oral pre-op information from their surgeon.  At the end of their pre-anesthesia appointment, also received an information booklet and were asked to read it multiple times. The booklet included 10 chapters covering numerous topics related to TKR. The booklet was developed and validated through a previous research study (13).  One of the outcomes investigated was patients' satisfaction with the information received for four specific items (hospital stay, TKR and TKR-related risks, possibilities of making home modifications and availability of human and financial help). Participants were asked to rate their satisfaction for each item on a 4-point Likert scale at 3-6 weeks post-op. The data were analysed using descriptive and inferential statistics. | **Patients aged 55-75 years old with knee OA listed for primary TKR**  **Control group:**  N=20  Age: 66.8±5.8  Gender: 50%  BMI: 31.6±5.4  **Intervention group:**  N=22  Age: 68.1±4.7  Sex: 45%  BMI: 31.2±5.1 | There were no significant between group differences in participants' satisfaction ratings for any of the four items.  In general participants’ satisfaction with information on the hospital stay and TKR and TKR-related risks was high. However, participants’ satisfaction with information on the possibility of making home modifications and the availability of human and financial help was generally lower and more varied between participants.  In the discussion, the authors report some participants commented that the possibility of making home modifications and the availability of financial and home help were not mentioned by the surgeon. |
| Goldsmith et al., 2017 (15), Canada | To explore patients’ experience and satisfaction post-TKR | **Mixed methods: Qualitative descriptive component of a mixed methods prospective cohort study**  Participants were purposively selected from a cohort study involving participants from six different sites across British Columbia.  The authors were particularly interested in exploring dissatisfaction, so recruited as many participants as possible who reported dissatisfaction with their TKR on the 6-month post-op questionnaire.  Data were collected using semi-structured interviews. The interviews focused on participants’ experiences of TKR and their satisfaction with the outcomes.  Data were analysed using a multi-step thematic coding process involving four coders and wider team discussions. | **Patients 8 months post-TKR**  N=45  Age: 65 (SD not reported)  Sex: 67% | Participants' views of their experiences of TKR were mainly focused on the aid/ assistance they received, which the authors described as 'support'. Participants' support expectations were formed across the care pathway. Participants whose support expectations were not met tended to report a negative experience of TKR. Participants' experiences of support were focused on three interacting areas: informational, clinical and personal.  Participants felt information about preparing for TKR and post-op recovery was key. Although participants felt the pre-op education sessions and their surgeon were important sources of informational support, many participants felt the information they provided was inadequate. Additionally, some participants felt it was difficult to understand and retain the instructions provided at the pre-op education session, particularly because the session was delivered to patients undergoing TKR and patients undergoing THR together.  Some participants reported that their surgeon did not have/make time to answer their questions and/or did not make an effort to treat them like an individual.  Key areas in which participants wanted more information included: pain expectations, pain management and recovery trajectories.  Participants felt gaining informational and emotional support from patients who had previously undergone TKR could be helpful and suggested providing ‘recovery stories’ of previous patients as part of the pre-op education. |
| Høvik et al., 2018 (16), Norway | To explore patients’ experiences of the first 2 weeks post-op following fast-track TKR | **Qualitative: Qualitative descriptive**  Participants were purposively selected from two different units of a University hospital.  Data were collected through three focus groups. Each focus group consisted of 3-5 participants.  The focus groups primarily aimed to explore patients’ experiences of pain, exercise and daily activities once they had returned home.  Data were analysed using systematic text condensation involving all three authors. An experienced qualitative researcher was also consulted to increase rigor. | **Patients 2 weeks post-TKR**  N=13  Age: 64.2 (SD not reported)  Gender: 62% | The key finding was that participants were resolute to cope at home. Four areas linked to this were identified:   1. Participants valued returning home. 2. Specific factors, including comprehensive education, helped participants prepare for early discharge and feel secure after returning home 3. Participants found sharing their experiences empowering. 4. Participants’ post-op pain experiences varied but they generally felt equipped to manage their pain.   Participants felt the pamphlet of written information was the most important source of information they received.  Participants appreciated meeting others at the pre-op education class. However, some participants heard other patients’ stories of serious complications during the class, which was frightening. |
| Huber et al., 2015b (17), Switzerland | To develop an instrument to assess the validity of a pre-op education program and to explore the instrument’s psychometric properties | **Quantitative descriptive embedded within an RCT: Questionnaire development and psychometric testing**  Participants were recruited from two hospitals. The study was embedded in an RCT that involved all participants attending a pre-op education program known as the KOPEI.  Data were collected through an instrument developed during the first stages of the study known as the KOPEQ. The KOPEQ was developed to assess the validity of the KOPEI and includes 16 items, each scored on a 5-point Likert scale.  Data were analysed using descriptive statistics, internal consistency was evaluated using Cronbach's alpha and an exploratory factor analysis was performed. | **Patients with knee OA listed for primary TKR**  N=35  Age:69.5±7.9  Gender/sex: 48.6%  BMI: 30.5 ± 5.5 | Participants’ responses to all the KOPEQ items were high, with the median for each item being 4 or 5. The KOPEQ covered participants’ overall impressions of the KOPEI and their views of intervention components and delivery approaches including: handouts, PowerPoint presentations, relation between theory and active participation, how well questions were answered, division of the education into three sessions and specific education sessions (‘Anatomy and function’, ‘Recommended activities’ and ‘Rehabilitation phases after surgery’).  The internal consistency of the KOPEQ was good. 61% of the variance was explained by a model with 4 factors, which the authors named 'didactics', 'addressability', 'empowerment' and 'theory'. |
| Lucas et al., 2013a, 2013b (18, 19), UK | To develop, implement and investigate the impact of a pre-op preparation program for patients undergoing TKR, including exploring the change processes involved using the Social Cognitive Theory | **Qualitative: Action research study**  Health professional and patient participants for the Project Management group were purposively selected from one acute NHS Trust. Additional patient participants from the same Trust were also recruited to explore the impact of the changes.  Data were collected through four action cycles focused on developing a patient education booklet and a multidisciplinary pre-op assessment/education clinic. The Social Cognitive Theory was used to frame the interventions.  The lead researcher was an ‘insider’ (nurse practitioner) at the NHS Trust where the study was conducted.  Data collection methods included observations, questionnaires, self-reporting, physiotherapist assessments, focus groups and semi-structured interviews.  Qualitative data were analysed using the approach described by Burnard^[[5]](#footnote-5)^. Quantitative data were analysed using descriptive statistics. | ***Project Management Group members***  **NHS Trust staff members**  N=17  N=12 (interviewed)  **Patients post-TKR**  N=5  N=4 (interviewed)  Age: 61 (total, SD not reported)  Gender/sex: 60% (total)  ***Participants recruited to explore the impact of the changes***  **Patients listed for TKR**  N=23 (total)  N=9 (participated in focus groups after their surgery) | The following action cycles were undertaken:   1. Development of an information booklet 2. Physical assessment and intervention in the pre-op clinic 3. Social assessment and intervention in the pre-op clinic 4. Service user involvement in the pre-op clinic   Various changes were implemented through the action cycles, including patients being given the information booklet, a home circumstances assessment form and an invitation to attend the pre-op clinic on the day they were listed for TKR. The pre-op clinic was delivered by a nurse practitioner, physiotherapist, occupational therapist and service users. The clinic included crutch and leg length measurements, education, teaching of post-op exercises, assessment of patients' home circumstances and service users sharing their experiences.  Patients felt the booklet and pre-op clinic increased their knowledge and self-efficacy. This helped them prepare for surgery and develop realistic expectations of the outcomes. Patients highlighted knowing how to use crutches and carry out post-op exercises as particularly helpful.  Some patients felt the exercises and advice provided at the pre-op clinic helped them reduce their pain and improve their function.  Various personal, environmental and behavioral factors that influenced the change process were identified. These included staff and services users’ self-efficacy beliefs, resource limitations and effective teamwork. Physiotherapists’, occupational therapists’ and nurse practitioners’ self-efficacy beliefs about their clinical skills in the pre-op clinic were high.  Service users felt they benefitted from volunteering at the pre-op clinic because it provided an opportunity to discuss their post-op recovery. |
| Plenge et al., 2018 (20), South Africa | To gain multi-disciplinary consensus on pre-op risk factors for poor outcomes, perioperative interventions for improving outcomes and important post-op outcomes for patients undergoing TKR and THR | **Quantitative descriptive: Delphi study**  Participants were recruited from public sector regional and central hospitals. The study focused on the following categories related to the care of patients undergoing primary elective unilateral TKR or THR in South Africa:   1. Risk factors for poor outcomes 2. Pre-op, intraoperative and post-op interventions for improving post-op outcomes 3. Patient and clinical outcomes for benchmarking care   The Delphi study consisted of four rounds. During the first round, participants were asked to make suggestions for each of the above categories. The suggestions were then grouped into statements. During the second and third rounds, participants were asked to rank the top 10 statements in each category and could also add further comments/references. During the fourth round, participants were offered the opportunity to participate in a Skype teleconference to discuss any disagreements with the rankings from the third round. A reverse scoring system was used for the rankings and respondents’ scores were summed to develop the ranked lists of priorities. | **Orthopaedic surgeons**  N=13  **Anesthetists**  N=12  **Physiotherapists**  N=8 | The number of suggestions in the first round for risk factors, pre-op interventions, intraoperative interventions, post-op interventions and outcomes were 247, 166, 144, 181 and 164 respectively. The pre-op intervention suggestions were grouped into 14 statements for the second round and 11 statements for the third round. The prioritized list of pre-op interventions did not change after the second round and was as follows:   1. A patient optimization clinic 2. Multidisciplinary planning 3. Patient education 4. Infection prevention 5. Establishing high-volume units 6. Smoking cessation 7. Optimization of pre-op analgesia regimen 8. Minimize pre-op fasting 9. Establish a patient blood management program 10. Alcohol cessation |
| Sharif et al., 2020 (21), UK | To gather an array of opinions on virtual healthcare technologies from key stakeholders within the NHS | **Qualitative: Qualitative descriptive**  Participants were selected from one NHS hospital trust using a 'process map' to ensure consistent representation across the elective hip and knee surgery pre-op pathway.  Data were collected using semi-structured interviews. The interviews explored participants' perspectives of the uses, benefits and problems with six key virtual health technologies identified in a systematic literature review.  Data were analysed using thematic analysis, with cross-checking at each stage by other team members. | **GPs**  N=2  **Orthopaedic surgeons**  N=2  **Anesthetists**  N=2  **Orthogeriatricians**  N=3  **Nurses**  N=3  **Occupational therapists**  N=2  **Physiotherapists**  N=2 | A wide range of uses, benefits and problems were identified for the virtual health technologies.  Pre-op education was perceived as a potential use for teleconsultations, web-based online videos, virtual reality, web-based written information/websites and m-health.  Pre-op exercise provision/encouragement/ monitoring were perceived as potential uses for web-based written information/websites, m-health and remote patient monitoring.  E-forms were also discussed, but their perceived uses focused on assessments/obtaining consent rather than pre-op interventions.  Accessibility issues related to digital illiteracy were identified as a potential problem with most technologies. Perceived benefits/ problems for specific technologies included:   - Teleconsultations: benefits – reduced patient travel and improved clinic efficiency; problems – hearing/ comprehension issues and lack of visual information - Web-based online videos: benefits – greater patient engagement and information retention; problems – lack of opportunity for patients to ask questions and difficulties providing individualised care - Virtual reality: main benefit – providing a visual aid; problems – risk of increasing patients’ anxiety and being disorientating for older individuals - Web-based written information/websites: benefits – easy access for patients and their families and being ‘more user-friendly’; main problem – limited accessibility due to digital illiteracy - M-health: benefits – being accessible to patients at all times and facilitating personalized care; problems – accessibility and compliance issues and ‘lack of human touch’ - Remote patient monitoring: benefits – providing more information to health professionals to facilitate management and streamlining the pathway; problems – possibility of abnormal readings alarming patients and accessibility issues due to digital illiteracy |
| Smith et al., 2018 (22), USA | To explore pain management and expectations pre- and post-TKR and THR surgery and identify barriers to post-op opioid tapering | **Qualitative: Qualitative descriptive**  Health professional participants were purposively selected from one ‘large, integrated care-delivery system’. Patient participants were also recruited through the same care system.  Data were collected through structured interviews with open-ended questions.  The interviews focused on participants’ views of opioids, pain management and associated educational materials.  If participants were willing, they were re-interviewed to help verify the interview findings and refine the educational materials developed.  Data were analysed using content analysis by a qualitative researcher. | **Patients 6-12 months post-TKR who were in the top 1/3 of opioid users during the first 90 days post-op**  N=4  Gender/sex: 100%  **Surgeons**  N=2  **Physician assistants**  N=2  **Nurses**  N=2  **Physical therapist**  N=1  (Patients post-THR: N=7, one of whom had a TKR prior to their THR) | The findings were reported in 4 main topic areas:   1. Pre-op pain management expectations and education 2. Post-op pain management experience 3. Challenges related to post-op pain management 4. Recommendations and suggestions for educational materials   Most patients felt the booklet and video they received pre-op did not cover pain management sufficiently but information from their surgeon visit was clear and useful^c^. However, three patients did not remember discussing pain management with their surgeon^c^.  Professionals reported that there is not often enough time to educate patients about opioids and highlighted patients receive extensive verbal and written information, which could result in ‘information fatigue.’  Endorsed recommendations highlight the importance of education on pain expectations and pain management (including opioids and non-opioid approaches). |
| Snowden et al., 2020 (23), UK | To investigate the feasibility of conducting a definitive trial of a brief behavioral intervention aimed at reducing pre-op alcohol consumption amongst patients listed for elective orthopaedic surgery | **Mixed methods: Non-randomized feasibility study followed by a two-arm pilot RCT**  ***Defining the intervention and treatment as usual***  As part of the intervention development, healthcare professionals employed in the pre-assessment clinic of the primary study site completed an adapted version of the COM-B self-evaluation questionnaire focused on delivering alcohol screening and a behavioral intervention. The questionnaire responses were summarized descriptively.  Treatment as usual in the pre-assessment clinic was characterized using focus groups with healthcare professionals from the three centers involved in the pilot RCT and a UK-wide electronic survey. Focus group data were analysed using framework analysis. Survey data were analysed descriptively. The survey data focused solely on describing service provision rather than exploring participants’ views.  ***Feasibility study***  Participants were recruited from one secondary care teaching hospital and screened for eligibility using the AUDIT-C. After consenting, they were asked to complete the full AUDIT. Participants who scored ≥8 on the AUDIT and/or consumed ≥6 units in one session weekly were eligible to receive the brief behavioral intervention. The intervention aimed to support participants to reduce their pre-op alcohol consumption. It was delivered by healthcare professionals working in the pre-assessment clinic in one face-to-face session at the clinic and involved:   - ~5 min of structured advice on alcohol consumption aimed at increasing the participant’s motivation, guided by the participant’s AUDIT score and a brief advice tool. - ~25 min of brief behaviour change counselling aimed at increasing the participant’s volition, guided by a brief intervention tool.   Participants received copies of the brief advice and brief intervention tools and a patient leaflet. An optional booster session was delivered approximately one week pre-op via telephone or face-to-face in the pre-assessment clinic. The  booster session involved completion of the AUDIT tool, goal review, feedback on performance and discussion of self-monitoring.  Data were collected through study statistics, intervention delivery fidelity assessments and qualitative interviews with patient participants and health professionals. Quantitative data were analysed descriptively. Qualitative data were analysed using framework analysis.  ***Pilot RCT***  Participants were recruited from three secondary care hospitals and randomized with 1:1 allocation to a control group or intervention group.  **Control group:**  Received treatment as usual and completed the AUDIT questionnaire.  **Intervention group:**  Received treatment as usual, completed the AUDIT questionnaire and received the brief behavioral intervention described above.  Data were collected through study statistics, questionnaires/tools, intervention delivery fidelity assessments and qualitative interviews with patient participants and health professionals.  Qualitative data were analysed using framework analysis. Quantitative data were analysed descriptively. The quantitative results were not reported separately for participants undergoing knee replacement. Two criteria for progression to a definitive trial were pre-specified. | ***Adapted COM-B self-evaluation questionnaire***  **Healthcare professionals**  N=12  ***Healthcare professional focus groups***  **Nurses**  N=14  **Student nurse**  N=1  **Substance use lead**  N=1  **Pre-assessment lead/team leader**  N=2  **Consultant anesthetist**  N=1  Gender/sex (all healthcare professionals who participated in the focus groups): 100%  ***Feasibility study***  **Adults listed for elective primary knee replacement who met the criteria for increased risk drinking**  N=12  (Adults listed for elective primary hip replacement who met the criteria for increased risk drinking N=3)  (13 participants completed an interview but details of these participants are not provided separately)  **Health professionals involved in the feasibility study**  N=3  ***Pilot RCT***  **Adults listed for elective primary knee replacement who met the criteria for increased risk drinking**  **Control group:**  N=25 (total)  N=6 (interviewed)  (Adults listed for elective primary hip replacement who met the criteria for increased risk drinking N=10 (total), N=1 (interviewed))  **Intervention group**  N=20 (total)  N=5 (interviewed)  (Adults listed for elective primary hip replacement who met the criteria for increased risk drinking N=13 (total), N=2 (interviewed))  **Healthcare professionals involved in the pilot RCT**  N=5  Gender/sex: 100%  (Demographic details are not provided separately for patients listed for knee replacement) | ***Adapted COM-B self-evaluation questionnaire***  Health professionals felt that they needed support to increase all three domains of capability, opportunity and motivation in order to deliver the screening and intervention effectively. Gaining more knowledge about the importance of pre-op alcohol cessation/reduction and having additional ‘protected’ time in the pre-assessment clinic were identified as particularly important.  ***Healthcare professional focus groups***  Key findings from the focus groups included:   - Patients are provided with a large volume of information in the pre-assessment clinic, which could be difficult for patients to process. - Some health professionals were not aware of the available alcohol specialist services. Professionals who were aware of the services reported that patients usually declined them and some professionals were concerned that patients referred to the services may not receive additional treatment/support.   ***Feasibility study and pilot RCT***  In the feasibility study, amendments to facilitate recruitment were made to the inclusion criteria, study title (including removal of the term ‘risky drinking’) and time-period for patients to consider their participation.  Key findings from the qualitative interviews conducted during the feasibility study and pilot RCT included:   - Patients and health professionals felt that the intervention was acceptable, but its impact on patients’ alcohol consumption varied widely. Health professionals identified potential benefits of the intervention at personal, system and society levels. - Some patients reported changing their drinking behaviour due to information about the consequences of alcohol consumption on post-operative recovery. Health professionals also felt that informing patients about the impact of alcohol consumption on recovery encouraged patients to engage with the intervention. - Patients were more familiar with the term ‘unit’ than ‘standard drink’ but their understanding of the term ‘unit’ varied. They were most comfortable discussing alcohol quantities in terms of pints or glasses. Patients’ views of the terms ‘harmful’, ‘hazardous’ and ‘risky’ drinking varied. - Patients’ opinions about whether patients would feel comfortable discussing their alcohol consumption varied. Health professionals felt that the discussions were a key part of the trial. - Health professionals felt that the brief advice tool and brief intervention tool were both useful and facilitated their communication with patients. - Some patients reported changing their drinking behaviour due to completing the AUDIT screening tool, which prompted them to think about how much they drink^c^. Health professionals also felt that the screening increased patients’ awareness of their alcohol consumption and helped motivate them to change. - Two healthcare professionals felt that pre-op assessment nurses are best placed to deliver the screening and intervention - Health professionals felt that the infographic explaining standard drinks was especially helpful. - Patients and health professionals felt that delivering the screening and intervention at the pre-assessment clinic was acceptable and highlighted that it was helpful to deliver the intervention around patients’ existing appointments so that patients do not have to make a separate trip. - Health professionals highlighted the importance of having allocated time to deliver the intervention. - Patients’ views about the possible benefits of the booster session varied. Some felt that it would be helpful whilst others felt that it would not make any difference. - Health professionals reported that tailoring the screening and interventions to patients' individual needs was important to keep their interactions positive.   In the pilot RCT, only 12% of intervention group participants received a booster session. One of pre-specified criteria for proceeding to a definitive trial was met and the other was not. Not meeting the recruitment target was mitigated by the high retention and data completion rates. The authors concluded that a definitive trial of the intervention is feasible and identified various modifications that would be beneficial, including removing the booster session from the intervention. |
| SooHoo et al., 2011 (24), USA | To develop evidence-based QIs on care components that can be addressed to reduce differences in complication rates amongst surgeons performing TKR and THR | **Quantitative descriptive: Modified Delphi study**  Participants were purposively selected through professional organizations related to total joint replacement.  Data were collected using the RAND/UCLA Appropriateness Method. An initial list of 101 QIs was developed based on semi-structured interviews with leaders in orthopaedic surgery and relevant literature.  The rating process involved two rounds in which participants anonymously rated the candidate QIs on a 1-9 validity scale, with a face-to-face discussion between the rounds. Data were analysed using descriptive statistics. The final list of QIs consisted of QIs with a median rating of ≥7 with agreement in the second round. | **Orthopaedic surgeons**  N=10 | All or part of 68 of the 101 candidate QIs were rated as valid with agreement by the panel and consisted of:   - 18 pre-op process QIs - 9 intraoperative process QIs - 10 post-op process QIs - 8 structural QIs linked to implant selection and technology use - 13 structural QIs linked to privileging of providers - 10 outcome and comorbidity-related QIs   Three QIs (16, 18 and 30) relate to pre-op education topics (treatment options, risks of surgery, functional outcomes, home modifications), delivery formats (written materials, electronic materials, videos) and/or providers (surgeon, nurse, case manager). |
| Specht et al., 2016 (25), Denmark | To explore the lived experience of patients undergoing fast-track primary TKR or THR between their first outpatient visit and discharge | **Qualitative: Phenomenological-hermeneutic**  Participants were recruited from one hospital. Selection was stratified according to surgery and sex, but was otherwise random.  Data were collected through observations and interviews. Participants were observed at various stages of the care pathway, including the pre-op outpatient clinic appointment and pre-op information session. Each participant took part in a semi-structured interview prior to discharge (other than one participant who was interviewed via telephone post-discharge). The interviews focused on important aspects of the fast-track program. Data analysis was guided by Paul Ricoeur’s theory of narrative and interpretation^[[6]](#footnote-6)^. The data analysis was performed by one researcher but the findings were discussed with other researchers. | **Patients undergoing primary TKR**  N=4  Age: 52.5±10.8  Sex: 50%  (Patients undergoing THR: N=4) | Three themes were identified:   1. Dealing with pain 2. Feelings of confidence or uncertainty – the meaning of information 3. Readiness for discharge   The information participants received created feelings of both reassurance and uncertainty.  Participants valued meeting care providers and talking to other patients at the pre-op information session.  Participants did however feel private and personal matters should be discussed individually rather than in group settings^c^.  Participants reporting having relatives present during the pre-op information session was helpful^c^.  Participants felt it was difficult to take on board information during the nurse conversation immediately after the decision to undergo surgery had been made, so having a leaflet to refer to was useful. One participant specifically highlighted that a large volume of information is provided during the nurse conversation and it was *‘quite mechanical’.* |
| Westby et al., 2018 (26), Canada | To develop QIs on pre-op and post-op rehabilitation for patients undergoing TKR or THR due to OA | **Quantitative descriptive: Modified Delphi study**  Participants were recruited through a networks of contacts, patient/professional organizations and leading clinical/research centers.  Data were collected using modified RAND/UCLA methodology. An initial list of 42 TKR QIs was developed from existing clinical practice guidelines, QIs, quality measures, systematic reviews, RCTs and cohort studies.  A total of three online rounds were used. During Rounds 1 and 3 participants rated each QI for importance and validity on a 1-9 Likert scale, during Round 2 participants took part in an online anonymous moderated discussion forum.  Data were analysed using descriptive statistics. The final set of QIs included all QIs with a median rating of ≥7 for importance and validity and no disagreement in Round 3. | **Orthopaedic surgeons**  N=5  **Family physician**  N=1  **Physiotherapists**  N=7  **Other allied health professionals**  N=2  **Methodological expert (health professional background not provided)**  N=1  **Patient who had undergone THR and TKR**  N=1  (Patient who had undergone THR: N=1 – the results include this participant)  Gender: 53% (for the 15 participants who completed the study) | No new TKR QIs were recommended during the rating process but wording alterations were made to the original QIs. Thirty six of the initial 42 TKR QIs were included in the final set of QIs and consisted of:   - 16 pre-op QIs - 10 acute care QIs - 8 post-acute care QIs - 2 across continuum QIs   All 16 QIs addressing pre-op TKR care from Round 1 were included in the final set of recommendations. Of the 16 pre-op TKR care QIs, 13 focused on screening/assessment and 3 focused on interventions. The QIs focused on pre-op TKR care addressed:   - Education (including education topics, providers, delivery modes and tailoring) - Exercise (including exercise types, schedule intensity and tailoring) - Lifestyle intervention (weight management program) |

*ADL* activities of daily living, *AUDIT* Alcohol Use Disorders Identification Test, *AUDIT-C* Alcohol Use Disorders Identification Test Consumption, *CBT* cognitive behavioral therapy, *CFIR* Consolidated Framework for Implementation Research, *COM-B* Capability, Opportunity and Motivation to perform a particular Behavior, *ERAS* Enhanced Recovery After Surgery, *GP* General Practitioner, *HADS* Hospital Anxiety and Depression Scale, *KOPEI* Knee Osteoarthritis Patient Education Intervention, *KOPEQ* Knee Osteoarthritis Patient Education Questionnaire, *NHS* National Health Service, *OA* osteoarthritis, *post-op* post-operative, *pre-op* pre-operative, *QI* quality indicator, *RCT* randomised controlled trial, *SD* standard deviation, *THR* total hip replacement, *TKR* total knee replacement, *UCLA* University of California Los Angeles, *UK* United Kingdom, *USA* United States of America

^a^ For studies with mixed populations, details of the participants and findings are only provided for participants who met the review eligibility criteria unless otherwise indicated. Where appropriate, details of participants who did not meet the review eligibility criteria are provided in brackets.

^b^ N indicates the number of participants who consented to participate. When reported in the primary source, age and BMI are presented as mean ± standard deviation in years and kg/m² respectively and gender/sex is presented as the percentage of females. Gender/sex is specified as either gender or sex if clearly reported in the primary source.

^c^ Finding is from a study with a mixed population and is not supported with evidence specifically for participants who met the review eligibility criteria.

**References**

1. Aunger JA, Greaves CJ, Davis ET, Asamane EA, Whittaker AC, Greig CA. A novel behavioural INTErvention to REduce Sitting Time in older adults undergoing orthopaedic surgery (INTEREST): results of a randomised-controlled feasibility study. Aging Clin Exp Res. 2020;32(12):2565-85.

2. Bardgett M, Lally J, Malviya A, Kleim B, Deehan D. Patient-reported factors influencing return to work after joint replacement. Occup Med (Lond). 2016;66(3):215-21.

3. Barnes RY, Bodenstein K, Human N, Raubenheimer J, Dawkins J, Seesink C, et al. Preoperative education in hip and knee arthroplasty patients in Bloemfontein. S Afr J Physiother. 2018;74(1):a436.

4. Berg U, Berg M, Rolfson O, Erichsen-Andersson A. Fast-track program of elective joint replacement in hip and knee-patients' experiences of the clinical pathway and care process. J Orthop Surg Res. 2019;14(1):186.

5. Bin Sheeha B, Williams A, Johnson DS, Granat M, Jones R. Patients' experiences and satisfaction at one year following primary total knee arthroplasty: A focus-group discussion. Musculoskeletal Care. 2020;18(4):434-49.

6. Causey-Upton R, Howell DM. Patient Experiences When Preparing for Discharge Home after Total Knee Replacement. The Internet Journal of Allied Health Sciences and Practice. 2017;15(1).

7. Causey-Upton R, Howell DM, Kitzman PH, Custer M, Dressler EV. Preoperative Education for Total Knee Replacement: a Pilot Survey. Internet Journal of Allied Health Sciences & Practice. 2018;16(4):1-12.

8. Causey-Upton R, Howell DM, Kitzman PH, Custer MG, Dressler EV. Preoperative Education for Total Knee Replacement: A National Survey of Orthopaedic Nurses. Orthop Nurs. 2020a;39(1):23-34.

9. Causey-Upton R, Howell DM, Kitzman PH, Custer MG, Dressler EV. Orthopaedic Nurses' Perceptions of Preoperative Education for Total Knee Replacement. Orthop Nurs. 2020b;39(4):227-37.

10. das Nair R, Mhizha-Murira JR, Anderson P, Carpenter H, Clarke S, Groves S, et al. Home-based pre-surgical psychological intervention for knee osteoarthritis (HAPPiKNEES): a feasibility randomized controlled trial. Clin Rehabil. 2018;32(6):777-89.

11. Drew S, Judge A, Cohen R, Fitzpatrick R, Barker K, Gooberman-Hill R. Enhanced Recovery After Surgery implementation in practice: an ethnographic study of services for hip and knee replacement. BMJ Open. 2019;9(3):e024431.

12. Judge A, Carr A, Price A, Garriga C, Cooper C, Prieto-Alhambra D, et al. The impact of the enhanced recovery pathway and other factors on outcomes and costs following hip and knee replacement: routine data study. Health Services and Delivery Research. 2020;No 8.4.

13. Eschalier B, Descamps S, Boisgard S, Pereira B, Lefevre-Colau MM, Claus D, et al. Validation of an educational booklet targeted to patients candidate for total knee arthroplasty. Orthop Traumatol Surg Res. 2013;99(3):313-9.

14. Eschalier B, Descamps S, Pereira B, Vaillant-Roussel H, Girard G, Boisgard S, et al. Randomized blinded trial of standardized written patient information before total knee arthroplasty. PLoS One. 2017;12(7):e0178358.

15. Goldsmith LJ, Suryaprakash N, Randall E, Shum J, MacDonald V, Sawatzky R, et al. The importance of informational, clinical and personal support in patient experience with total knee replacement: a qualitative investigation. BMC Musculoskelet Disord. 2017;18(1):127.

16. Høvik LH, Aglen B, Husby VS. Patient experience with early discharge after total knee arthroplasty: a focus group study. Scand J Caring Sci. 2018;32(2):833-42.

17. Huber EO, Bastiaenen CH, Bischoff-Ferrari HA, Meichtry A, de Bie RA. Development of the knee osteoarthritis patient education questionnaire: a new measure for evaluating preoperative patient education programmes for patients undergoing total knee replacement. Swiss Med Wkly. 2015;145:w14210.

18. Lucas B, Cox C, Perry L, Bridges J. Pre-operative preparation of patients for total knee replacement: An action research study. Int J Orthop Trauma Nurs. 2013a;17(2):79-90.

19. Lucas B, Cox C, Perry L, Bridges J. Changing clinical team practices in preparation of patients for Total Knee Replacement: Using Social Cognitive Theory to examine outcomes of an action research study. Int J Orthop Trauma Nurs. 2013b;17(3):140-50.

20. Plenge U, Nortje MB, Marais LC, Jordaan JD, Parker R, van der Westhuizen N, et al. Optimising perioperative care for hip and knee arthroplasty in South Africa: a Delphi consensus study. BMC Musculoskelet Disord. 2018;19(1).

21. Sharif F, Rahman A, Tonner E, Ahmed H, Haq I, Abbass R, et al. Can technology optimise the pre-operative pathway for elective hip and knee replacement surgery: a qualitative study. Perioper Med (Lond). 2020;9(1):33.

22. Smith DH, Kuntz J, DeBar L, Mesa J, Yang X, Boardman D, et al. A qualitative study to develop materials educating patients about opioid use before and after total hip or total knee arthroplasty. J Opioid Manag. 2018;14(3):183-90.

23. Snowden C, Lynch E, Avery L, Haighton C, Howel D, Mamasoula V, et al. Preoperative behavioural intervention to reduce drinking before elective orthopaedic surgery: the PRE-OP BIRDS feasibility RCT. Health Technol Assess. 2020;24(12):1-176.

24. SooHoo NF, Lieberman JR, Farng E, Park S, Jain S, Ko CY. Development of quality of care indicators for patients undergoing total hip or total knee replacement. BMJ Qual Saf. 2011;20(2):153-7.

25. Specht K, Kjaersgaard‐Andersen P, Pedersen BD. Patient experience in fast-track hip and knee arthroplasty - a qualitative study. J Clin Nurs. 2016;25(5-6):836-45.

26. Westby MD, Marshall DA, Jones CA. Development of quality indicators for hip and knee arthroplasty rehabilitation. Osteoarthritis Cartilage. 2018;26(3):370-82.

1. Bubela N, Galloway S, McCay E, McKibbon A, Nagle L, Pringle D et al. The Patient Learning Needs Scale: reliability and validity. J Adv Nurs.1990;15(10);1181-1187. [↑](#footnote-ref-1)
2. Soever LJ, Mackay C, Saryeddine T, Davis AM, Flannery JF, Jaglal SB et al. Educational needs of patients undergoing total joint arthroplasty. Physiotherapy Canada. Physiotherapie Canada. 2010;62(3);206-214. [↑](#footnote-ref-2)
3. Moustakas C. Phenomenological Research Methods. Thousand Oaks (CA): SAGE Publications, Inc.;1994. [↑](#footnote-ref-3)
4. Moustakas C. Phenomenological Research Methods. Thousand Oaks (CA): SAGE Publications, Inc.;1994. [↑](#footnote-ref-4)
5. Burnard P. A method of analysing interview transcripts in qualitative research. Nurse Educ Today. 1991,11(6):461-466. [↑](#footnote-ref-5)
6. Ricoeur P. Interpretation Theory: Discourse and the Surplus of Meaning. Texas: Texas Christian University Press; 1976. [↑](#footnote-ref-6)
